# Arsenic-sensing domain controls ACR3 transporter trafficking in *Marchantia polymorpha*

**DOI:** 10.1093/plcell/koag306

**Published:** 2026-10-06

**Authors:** Ignacy Bonter, Katarzyna Mizio, Alicja Dolzblasz, Paulina Tomaszewska, Kacper Zbieralski, Jacek Staszewski, Donata Wawrzycka, Anna Reymer, Wojciech Bialek, Verena Kriechbaumer, Jim Haseloff, Robert Wysocki, Ewa Maciaszczyk-Dziubinska

**Affiliations:** Department of Genetics and Cell Physiology, Faculty of Biological Sciences, University of Wroclaw, Kanonia 6/8, 50-328 Wroclaw, Poland; Department of Plant Sciences, University of Cambridge, Downing Street, Cambridge CB2 3EA, United Kingdom; Department of Genetics and Cell Physiology, Faculty of Biological Sciences, University of Wroclaw, Kanonia 6/8, 50-328 Wroclaw, Poland; Department of Plant Developmental Biology, Faculty of Biological Sciences, University of Wroclaw, Kanonia 6/8, 50-328 Wroclaw, Poland; Department of Genetics and Cell Physiology, Faculty of Biological Sciences, University of Wroclaw, Kanonia 6/8, 50-328 Wroclaw, Poland; Genetics and Genome Biology, University of Leicester, Leicester LE1 7RH, United Kingdom; Department of Genetics and Cell Physiology, Faculty of Biological Sciences, University of Wroclaw, Kanonia 6/8, 50-328 Wroclaw, Poland; Department of Genetics and Cell Physiology, Faculty of Biological Sciences, University of Wroclaw, Kanonia 6/8, 50-328 Wroclaw, Poland; Department of Genetics and Cell Physiology, Faculty of Biological Sciences, University of Wroclaw, Kanonia 6/8, 50-328 Wroclaw, Poland; Department of Chemistry and Molecular Biology, University of Gothenburg, Göteborg 405 30, Sweden; Université Grenoble Alpes, Inserm U 1209, CNRS UMR 5309, ReTraD, Institute for Advanced Biosciences, Grenoble 38000, France; Department of Biophysics, Faculty of Biotechnology, University of Wroclaw, F. Joliot-Curie 14a, 50-383 Wroclaw, Poland; School of Biological and Medical Sciences and Centre for Bioimaging, Oxford Brookes University, Gipsy Lane, Headington, Oxford OX3 0BP, United Kingdom; Department of Plant Sciences, University of Cambridge, Downing Street, Cambridge CB2 3EA, United Kingdom; Department of Genetics and Cell Physiology, Faculty of Biological Sciences, University of Wroclaw, Kanonia 6/8, 50-328 Wroclaw, Poland; Department of Genetics and Cell Physiology, Faculty of Biological Sciences, University of Wroclaw, Kanonia 6/8, 50-328 Wroclaw, Poland

## Abstract

Arsenic is a toxic, carcinogenic metalloid that threatens human health through contaminated water and food. Arsenite [As(III)] efflux mediated by ACR3 transporters is an ancient detoxification mechanism conserved across prokaryotes, fungi, algae, and non-angiosperm land plants, yet plant ACR3 proteins remain poorly characterized. Here, we show that MpACR3, the ACR3 ortholog from the model liverwort *Marchantia polymorpha*, contains an arsenic-responsive N-terminal domain that regulates intracellular trafficking. Under nonstress conditions, MpACR3 is detected in Golgi bodies as well as at the plasma membrane (PM) in plant cells, whereas As(III) promotes its relocalization to the PM. As(III) interacts with a cysteine-rich N-terminal region and stabilizes this domain, as shown by arsenite-biotin competition and increased protease resistance. Together with molecular dynamics simulations, which predict As(III)-dependent changes in secondary structure and surface properties, these findings support a model in which ligand-induced remodeling of the N-terminus modulates MpACR3 retention and trafficking. We further identify a di-arginine-containing LRCRF motif in which leucine and arginine residues are required for intracellular retention, whereas cysteine and phenylalanine residues are required for As(III)-induced relocalization to the PM. The broad conservation of related N-terminal domains in plant ACR3 transporters suggests that arsenic-regulated trafficking is a conserved plant adaptation to arsenic toxicity.

## Introduction

Arsenic is a toxic element that occurs naturally in the environment and, through both geogenic processes and anthropogenic activities, can accumulate to dangerously high concentrations in certain regions (Oremland and Stolz 2003; Wysocki et al. 2023). Arsenic contamination of groundwater has been reported in more than 100 countries, posing a potential health risk to up to 220 million people worldwide (Rahaman et al. 2021). The main routes of human exposure are drinking water (Podgorski and Berg 2020) and food products, including cereals, particularly rice and wheat-based products, as well as fruits and fruit products, vegetables, seafood, and animal-derived products (Zhao et al. 2024). Epidemiological studies have consistently linked chronic arsenic exposure to multiple adverse health outcomes, including increased risks of coronary heart disease and several cancers (Naujokas et al. 2013; Oberoi et al. 2019). Accordingly, the International Agency for Research on Cancer, the specialized cancer agency of the World Health Organization, has classified arsenic and inorganic arsenic compounds as carcinogenic to humans. Arsenic also ranks first on the U.S. Agency for Toxic Substances and Disease Registry priority list of substances posing the greatest potential risk to human health.

Arsenite [As(OH)_3_ or As(III)] and arsenate [ $H_{2}{\mathrm{AsO}}_{4}^{-}/{\mathrm{HAsO}}_{4}^{2-}$ or As(V)] are the predominant inorganic arsenic species in water and soil (Jain and Ali 2000). Owing to molecular mimicry, As(III) and As(V) are readily taken up by cells across diverse organisms through aquaglyceroporin channels and phosphate transporters, respectively (Maciaszczyk-Dziubinska et al. 2012; Garbinski et al. 2019). ACR3-family As(III) efflux transporters, which confer high-level resistance to both As(III) and As(V), are widely distributed across bacteria, archaea, and most eukaryotic lineages, highlighting their importance as an evolutionary adaptation to environmental arsenic exposure (Indriolo et al. 2010; Maciaszczyk-Dziubinska et al. 2012; Garbinski et al. 2019; Chen et al. 2020). Notably, however, ACR3 genes are absent from angiosperms (Indriolo et al. 2010). ACR3 transporters have been well characterized in bacteria and budding yeast, where they localize to the plasma membrane (PM) and mediate As(III) extrusion (Bobrowicz et al. 1997; Wysocki et al. 1997; Sato and Kobayashi 1998; Ghosh et al. 1999; Fu et al. 2009; Maciaszczyk-Dziubinska et al. 2010, 2011; Villadangos et al. 2012). ACR3 proteins characterized to date share key structural and functional features, including 10 transmembrane regions with both the N- and C-termini facing the cytoplasm, As(III)/H^+^ antiporter activity, and highly conserved cysteine and glutamic acid residues required for transport function (Maciaszczyk-Dziubinska et al. 2011; Villadangos et al. 2012; Wawrzycka et al. 2017).

Expression of the *Saccharomyces cerevisiae ACR3* (*ScACR3*) gene in *Arabidopsis thaliana* (hereafter Arabidopsis) and *Oryza sativa*, both naturally lacking *ACR3*, enhanced tolerance to As(III) and As(V) while promoting increased arsenic efflux from roots to the external environment (Ali et al. 2012; Duan et al. 2012). However, while Arabidopsis plants expressing *ScACR3* did not show reduced arsenic levels in their shoots (Ali et al. 2012), another study reported that *ScACR3* expression significantly lowered arsenic accumulation in rice straw and grains (Duan et al. 2012). Beyond *ScACR3*, the only other eukaryotic orthologs analyzed to date are from the fern genus *Pteris* (Indriolo et al. 2010; Chen et al. 2021; Popov et al. 2021; Sun et al. 2023), and, more recently, from the model bryophyte *Marchantia polymorpha* (hereafter Marchantia) (Dutta et al. 2024; Li et al. 2024). Notably, the model green alga *Chlamydomonas reinhardtii*, which lacks an *ACR3* ortholog, is 10 times more sensitive to As(V) than the acidophilic green alga *C. eustigma*, which expresses *ACR3* (Hirooka et al. 2017). This suggests that the *C. eustigma ACR3* gene (*CeACR3*) encodes a functional arsenic transporter.

*Pteris vittata* is a well-characterized arsenic hyperaccumulator that expresses up to 5 *ACR3* paralogs: *PvACR3*, *PvACR3;1*, *PvACR3;2*, *PvACR3;3*, and *PvACR3;4* (Indriolo et al. 2010; Chen et al. 2017, 2021; Sun et al. 2023). Heterologous overexpression of *P*. *vittata ACR3* genes in a *S*. *cerevisiae acr3*Δ strain lacking the endogenous *ACR3* gene, as well as in Arabidopsis or *Nicotiana tabacum* (hereafter tobacco) has shown that these transporters enhance tolerance to both As(III) and As(V) (Indriolo et al. 2010; Chen et al. 2013, 2017, 2021; Wang et al. 2018). Moreover, silencing of the *PvACR3* gene, but not the *PvACR3;1* gene in *P. vittata* gametophytes resulted in increased sensitivity to As(III) (Indriolo et al. 2010). Interestingly, *P. vittata* ACR3 proteins exhibit a distinct subcellular localization pattern that likely contributes to arsenic hyperaccumulation in this species. PvACR3 was reported to localize to the vacuolar membrane in *P*. *vittata* gametophytes (Indriolo et al. 2010). Similarly, when expressed in Arabidopsis and tobacco, PvACR3;1 and PvACR3;3 localized to the vacuolar membrane, facilitating As(III) sequestration in the vacuole and its retention in root cells (Chen et al. 2017; Chen et al. 2021). By contrast, PvACR3 and PvACR3;2 were targeted to the PM in transgenic plants, where they not only mediated As(III) extrusion from root cells into the external environment but also promoted As(III) translocation from roots to shoots (Chen et al. 2013; Wang et al. 2018; Chen et al. 2021).

In Marchantia, the MpACR3 ortholog plays a key role in arsenic detoxification, as loss-of-function mutants exhibit hypersensitivity to As(III) and As(V), together with elevated arsenic accumulation. Conversely, Mp*ACR3*-overexpressing transgenic lines show enhanced arsenic tolerance and lower arsenic levels in plant tissues compared with wild-type plants (Li et al. 2024). Consistent with this function, the MpACR3-Citrine fusion protein is localized to the PM in Marchantia cells. Interestingly, MpACR3-Citrine signals were also detected in unidentified intracellular bodies, possibly reflecting folding or sorting defects associated with overexpression of the fusion protein, as suggested by Li et al. (2024).

In this study, we show that, under control conditions, MpACR3 is retained in intracellular compartments, localizing predominantly to the endoplasmic reticulum (ER) in yeast and to Golgi bodies in plants, including Marchantia, Arabidopsis, and tobacco, whereas arsenic exposure promotes its accumulation at the PM. Intracellular localization of MpACR3 requires an arginine-based ER/Golgi retention motif conserved in several plant ACR3 proteins, whereas arsenic-induced relocalization to the PM depends on a triad of cysteine residues; both determinants are located within the N-terminal domain. We provide evidence that arsenic binding triggers a conformational change in this domain, which may facilitate MpACR3 release from intracellular retention. We propose that the N-terminal region of MpACR3 functions as a metalloid-sensing module that couples arsenic binding to PM trafficking of the transporter. This discovery reveals a previously unrecognized evolutionary adaptation of plant ACR3 transporters and provides new insights into the cellular mechanisms underlying arsenic sensing and detoxification in plants.

## Results

### Predicted structure of MpACR3

The *M. polymorpha* genome encodes a single *ACR3* gene (*MpACR3*, Mp5g06690), which is predicted to produce a 457-amino-acid protein with 10 transmembrane helices (TM1–TM10) and both the N- and C-termini facing the cytoplasm (Figures S1 and S2). MpACR3 shares 44.3%, 40.5%, and 66.3% sequence identity with previously characterized ACR3 transporters from the bacterium *Corynebacterium glutamicum* (CgAcr3), the yeast *S. cerevisiae* (ScAcr3), and the fern *P. vittata* (PvACR3), respectively (Figure S1).

Consistent with other ACR3-family members (Lv et al. 2022; Mizio et al. 2023), the AlphaFold-predicted structure of MpACR3 (Entry ID A0A176WR38) (Jumper et al. 2021) comprises 2 distinct domains: a panel domain, formed by α-helices TM1-TM2 and TM6-TM7, and a transport domain, formed by α-helices TM3-TM5 and TM8-TM10. The transport domain contains a crossover region formed by the discontinuous α-helices TM4 and TM9 (Figure S2). This predicted architecture is consistent with the highly conserved Na^+^/H^+^ fold (Hunte et al. 2005), which is also shared by members of the bile acid/sodium symporter (BASS) family. Together with ACR3 transporters, BASS proteins belong to the bile/arsenite/riboflavin transporter superfamily (Mansour et al. 2007). Crystal structures of bacterial homologs of the human apical sodium-dependent bile acid transporter from *Yersinia frederiksenii* and *Neisseria meningitidis* revealed a 10-transmembrane topology with both termini located in the cytoplasm, as well as a conserved crossover region (Hu et al. 2011; Zhou et al. 2014), consistent with predictions for ACR3 proteins. Although crystal structures of ACR3-family transporters are not yet available, a 10-transmembrane topology with cytoplasmic N- and C-termini has been experimentally confirmed for bacterial and yeast ACR3 transporters (Aaltonen and Silow 2008; Villadangos et al. 2012; Wawrzycka et al. 2017).

A notable feature of MpACR3 is its unusually long N-terminal tail of 123 amino acids (Figure S1), which is predicted to form a cytosolic domain containing a β-meander motif (Figure S2). Interestingly, N-terminal extensions of this type, ranging from 104 to 163 residues and enriched in cysteine residues, are a common feature of plant ACR3 orthologs (Figure S3). Only a few plant ACR3 sequences, primarily from ferns and certain algae, contain N-terminal tails shorter than 100 residues (Figure S4).

### Arsenic and antimony induce MpACR3 relocalization from the ER to the PM in yeast

To investigate the function and subcellular localization of MpACR3, we first used heterologous expression in *S. cerevisiae acr3*Δ mutant cells, which are sensitive to arsenic because they lack the endogenous *ACR3* gene (Wysocki et al. 1997; Maciaszczyk-Dziubinska et al. 2010, 2011). Mp*ACR3* cDNA, isolated from *M. polymorpha* Cam-1 gametophytes, was cloned under the control of the strong *MET17* promoter and fused to a C-terminal GFP reporter. The *acr3*Δ strain was then transformed with the resulting plasmid pMpACR3. Western blot analysis confirmed detectable expression of MpACR3-GFP in the transformed yeast strain (Fig. 1a). As(III) or As(V) exposure did not affect the level of MpACR3-GFP protein. Interestingly, treatment with As(III) induced the appearance of a slower-migrating form of MpACR3-GFP, the origin of which remains unclear (Fig. 1a). Consistent with previous findings (Dutta et al. 2024), functional assays showed that expression of MpACR3-GFP complemented the arsenic sensitivity of *acr3*Δ yeast cells (Fig. 1b).

**Figure 1:**
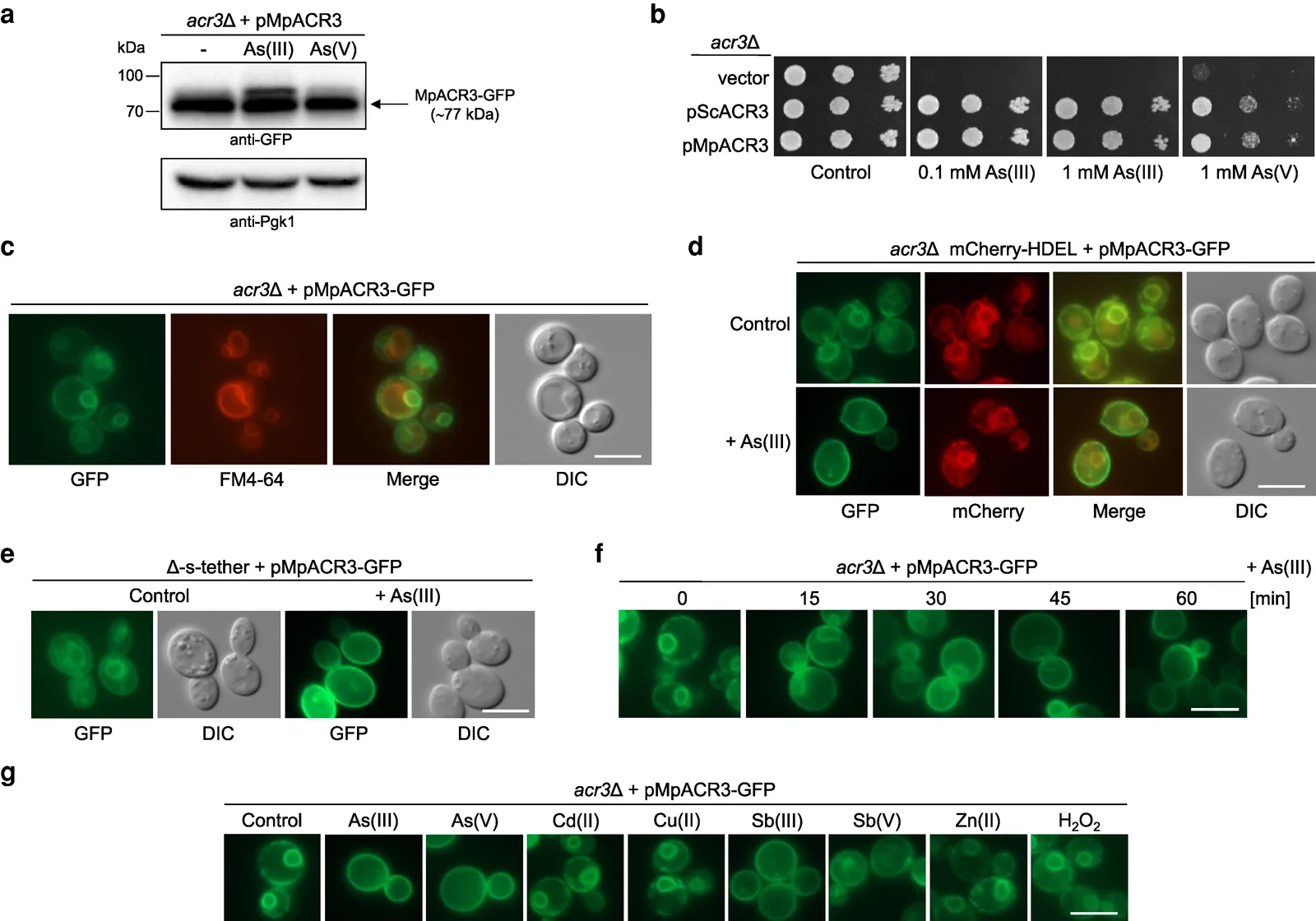
Functional characterization of MpACR3 in budding yeast. a) Western blot analysis of MpACR3-GFP fusion protein. Cultures were treated with 0.1 mM As(III), 0.5 mM As(V), or left untreated for 2 h prior to protein extraction. Pgk1 (3-phosphoglycerate kinase) served as a loading control. b) Growth assays showing that MpACR3-GFP expression rescues the arsenic-sensitive phenotype of *acr3*Δ cells. The *S. cerevisiae acr3*Δ strain was transformed with the following plasmids: pUG35, an empty-vector negative control, pScACR3, a positive control expressing the *S. cerevisiae ACR3-GFP* fusion gene under the *MET17* promoter, or pMpACR3, expressing *proMET17:*Mp*ACR3-GFP*. c) Under control conditions, MpACR3-GFP displays intracellular localization distinct from the vacuolar membrane. Yeast vacuolar membranes were visualized by FM4-64 staining. d) MpACR3-GFP colocalized with the mCherry-HDEL ER marker and relocalized to the PM in response to As(III). Yeast cells were treated with 0.1 mM As(III) for 2 h or left untreated prior to fluorescence microscopy. e) In the Δ-s-tether strain lacking ER-PM contact sites, MpACR3-GFP displayed clear ER- and PM-associated signals in the absence of As(III). After exposure to 0.1 mM As(III) for 2 h, MpACR3-GFP accumulated predominantly at the PM. f) Kinetics of MpACR3-GFP relocalization from the ER to the PM following the addition of 0.1 mM As(III). g) Loss of MpACR3-GFP localization in the ER was specifically triggered by arsenic and antimony compounds. Yeast cultures were exposed for 2 h to 0.1 mM As(III) (sodium arsenite), 0.5 mM As(V) (sodium arsenate), 0.01 mM Cd(II) (cadmium chloride), 0.05 mM Cu(II) (copper sulfate), 1 mM Sb(III) (potassium antimonyl tartrate), 0.5 mM Sb(V) (sodium antimonate), 0.1 mM Zn(II) (zinc sulfate), or 0.25 mM H_2_O_2_. DIC, differential interference contrast. Scale bars = 5 μm.

To investigate the subcellular localization of the MpACR3-GFP fusion protein in greater detail, we examined the distribution of the GFP signal in yeast cells by fluorescence microscopy. Under control conditions, the MpACR3-GFP signal appeared as patches at the cell periphery and as a ring-shaped intracellular structure, likely corresponding to the cortical and perinuclear ER, respectively (Fig. 1c) (Young et al. 2013). Moreover, the MpACR3-GFP signal did not align with the vacuolar periphery, which is morphologically distinct in differential interference contrast (DIC) microscopy, nor did it colocalize with FM4-64-labeled vacuolar membranes (Fig. 1c). To confirm the ER localization of MpACR3-GFP, we next used the *acr3*Δ strain expressing mCherry-HDEL, a red fluorescent ER marker carrying the HDEL retention signal (Zhu et al. 2019). Fluorescence microscopy showed that MpACR3-GFP largely colocalized with mCherry-HDEL, confirming its presence in the ER (Fig. 1d). Interestingly, after 2 h of exposure to 0.1 mM As(III), the ER-associated MpACR3-GFP signal largely disappeared, whereas the GFP signal at the cell surface became more uniform (Fig. 1d). These results strongly suggest that, under control conditions, MpACR3-GFP is retained in the ER but relocalizes to the PM in response to As(III).

Because the cortical ER is closely associated with the PM in yeast cells, the ER-associated MpACR3-GFP signal may overlap with the PM signal. To resolve these signals, we expressed MpACR3-GFP in a Δ-s-tether yeast mutant, which lacks 7 ER-PM tethering proteins and therefore shows a near absence of PM-associated ER (Quon et al. 2018). In the Δ-s-tether strain, MpACR3-GFP remained localized to the ER, but a distinct signal was also observed at the cell surface (Fig. 1e), suggesting that, under nonstress conditions, a subset of MpACR3-GFP is also present at the PM. Upon As(III) exposure, the ER-associated MpACR3-GFP signal largely disappeared, and the protein accumulated predominantly at the PM (Fig. 1e). Interestingly, MpACR3-GFP remained stable during the cycloheximide chase assay, arguing against ER-associated degradation under these conditions (Figure S5). We also examined the kinetics of MpACR3-GFP relocalization from the ER to the PM. Upon addition of 0.1 mM As(III), MpACR3-GFP gradually accumulated at the cell periphery, with the ER-associated GFP signal nearly disappearing within 45–60 min (Fig. 1f).

To determine whether this intracellular redistribution represented a specific response to arsenic or reflected a broader cellular response to oxidative stress or metal(loid)-mediated interactions with cysteine thiol groups in proteins, we exposed cells expressing MpACR3-GFP to hydrogen peroxide and a range of metal(loid)s, including As(V), Cd(II), Cu(II), Sb(III), Sb(V), and Zn(II) (Fig. 1g). MpACR3-GFP remained retained in the ER under all tested conditions, except for arsenic and antimony species, which triggered its redistribution to the PM. The observation that both trivalent and pentavalent forms of arsenic and antimony induced MpACR3 accumulation at the PM may reflect intracellular reduction of As(V) and Sb(V) to their trivalent forms, as reported for arsenate reduction by ACR2-like enzymes (Mukhopadhyay et al. 2000). Together, these findings indicate that MpACR3 relocalization from the ER to the PM is a specific response to arsenic and antimony species rather than a general stress-induced cellular reaction.

GFP has been widely used as a topology reporter for membrane proteins because it generally folds and matures efficiently in the cytosol but not in extracytoplasmic compartments (Drew et al. 2002). Thus, fluorescence of the C-terminal GFP fusion is consistent with the predicted cytosolic orientation of the MpACR3 C-terminus (Fig. 1d–g). Together with the predicted 10-transmembrane topology of MpACR3, this finding strongly supports a cytosolic orientation of both the N- and C-termini (Figures S1 and S2).

### MpACR3 enhances arsenic tolerance in Arabidopsis

To investigate the properties of MpACR3 in plants, we first transformed wild-type Arabidopsis, which lacks an ACR3 ortholog, with MpACR3-GFP under the control of the constitutive CaMV 35S promoter. Two independent homozygous lines expressing Mp*ACR3-GFP*, T21 and T27, were selected for further analysis. Under control conditions, wild-type and *_pro_35S:*Mp*ACR3-GFP* plants grown in soil showed indistinguishable phenotypes (Figure S6). MpACR3-GFP was detected exclusively in extracts from transgenic plants as an ∼77-kDa band, consistent with the expected molecular mass of the fusion protein (Fig. 2a). Interestingly, exposure to either As(III) (5 μM) or As(V) (100 μM) for 2 weeks increased MpACR3-GFP protein levels compared with untreated controls, without the appearance of additional slow-migrating forms (Fig. 2a), in contrast to its expression in yeast (Fig. 1a). The mechanism underlying this increase, which may involve transcriptional or posttranscriptional regulation, was not investigated further.

**Figure 2:**
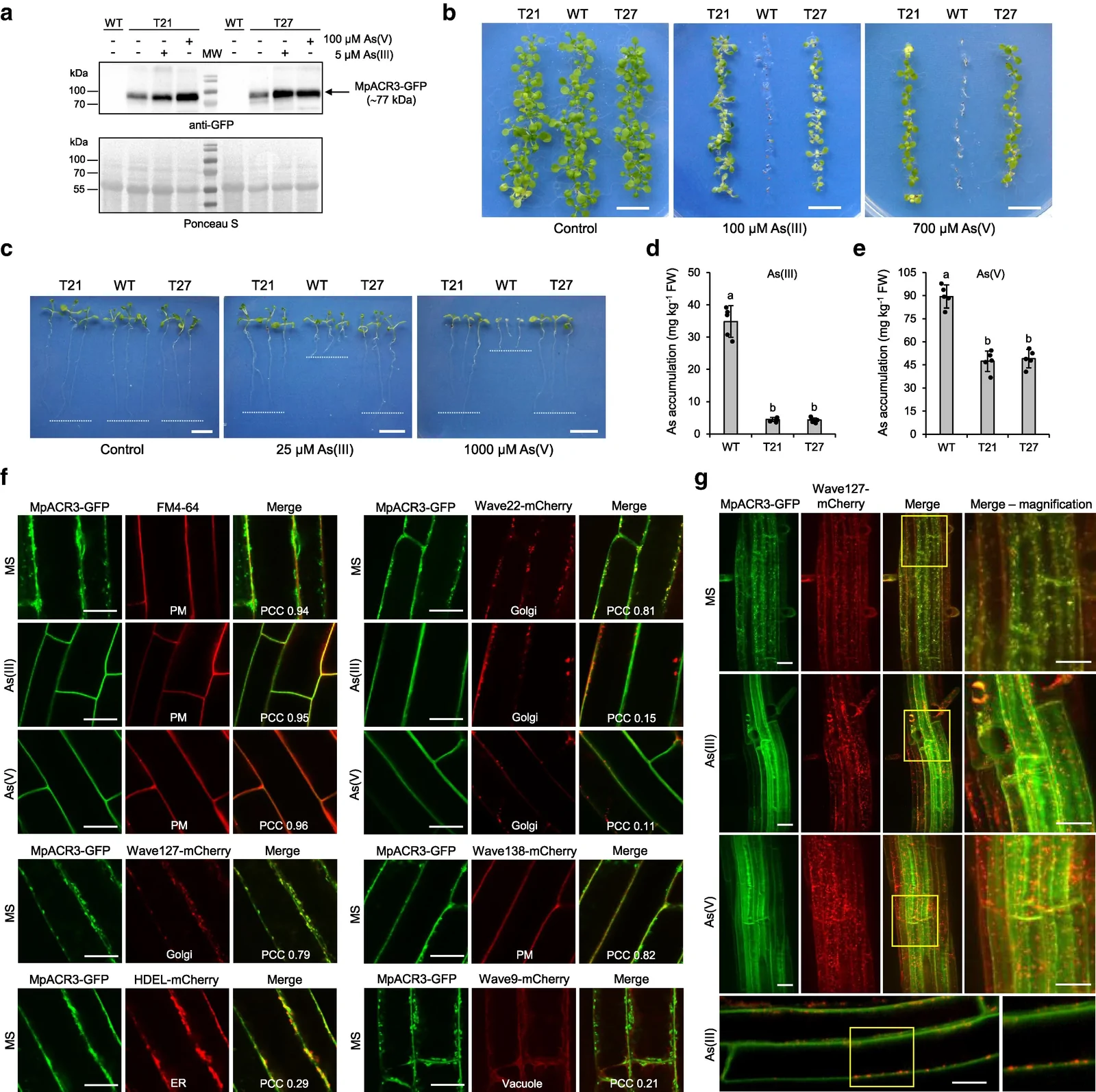
Functional characterization of MpACR3-GFP in Arabidopsis. a) Western blot analysis of MpACR3-GFP protein levels in Arabidopsis *_pro_35S:*Mp*ACR3-GFP* transgenic lines, T21 and T27, grown for 2 weeks in the presence of 5 μM As(III), 100 μM As(V), or under control conditions, MS medium. Protein extracts from wild-type plants (WT, Col-0) were used as a negative control. Ponceau S staining of the membrane served as a protein loading control. MW, molecular weight marker. b) Seed germination and early seedling growth of WT and transgenic lines, T21 and T27, in the absence or presence of As(III) and As(V). Plates were photographed after 2 weeks of growth. Scale bars = 1 cm. c) Growth phenotype of WT and transgenic seedlings, T21 and T27, grown for 4 d on MS medium followed by 7 d in the absence or presence of As(III) and As(V). Scale bars = 1 cm. d) Arsenic accumulation in WT and transgenic (T21 and T27) seedlings grown in the presence of 10 μM As(III) for 2 weeks. e) Arsenic accumulation in WT and transgenic T21 and T27 seedlings grown in the presence of 200 μM As(V) for 2 weeks. For (d) and (e), data are presented as mean ± SD from 5 biological replicates; each replicate consisted of pooled seedlings from an independent experiment. Statistical significance was assessed separately for each arsenic species using Welch's ANOVA followed by Games–Howell post hoc test for As(III), and one-way ANOVA followed by Tukey's HSD test for As(V). Different letters indicate statistically significant differences at *P*-value < 0.05. FW, fresh weight. f) Subcellular localization of MpACR3-GFP in Arabidopsis root cells visualized by confocal microscopy. Four-day-old seedlings of transgenic Arabidopsis lines expressing MpACR3-GFP, either alone or together with compartment-specific markers, were transferred to MS medium without or supplemented with 25 µM As(III) or 1,000 µM As(V), followed by an additional 1 d incubation before confocal microscopy analysis. The plasma membrane (PM) was visualized by short-pulse FM4-64 staining followed by immediate confocal imaging to minimize dye internalization through the endocytic pathway. Transgenic Arabidopsis lines are described in the Materials and Methods section. Pearson's correlation coefficient (PCC) was used to quantify colocalization of MpACR3-GFP with the indicated compartment markers or FM4-64-stained PM. Scale bars = 16 μm. g) Imaging of transgenic Arabidopsis roots expressing MpACR3-GFP and the Golgi marker (Wave127-mCherry) using a ZEISS LLS7 microscope. Four-day-old seedlings were transferred to either control MS medium or MS medium supplemented with 25 µM As(III) or 1,000 µM As(V), followed by an additional 1 d incubation prior to microscopy. Root images are shown either as 3D reconstructions or as 2D single optical sections (bottom panel). Yellow boxes indicate regions shown at higher magnification. Scale bars = 50 µm (3D reconstructions) and 15 µm (2D optical sections).

To assess the effect of *_pro_35S:*Mp*ACR3-GFP* expression on Arabidopsis growth under arsenic stress, we examined seed germination and seedling growth on MS medium supplemented with As(III) or As(V). In the absence of arsenic, no differences were observed between wild-type and *_pro_35S:*Mp*ACR3-GFP* plants in any assay (Fig. 2b and c). However, in the presence of 100 μM As(III) or 700 μM As(V), germination of *_pro_35S:*Mp*ACR3-GFP* seeds was largely maintained, whereas wild-type germination was strongly inhibited (Fig. 2b). Furthermore, when seeds were germinated on standard MS medium for 4 d and then transferred to medium containing 25 μM As(III) or 1,000 μM As(V), wild-type plants exhibited more pronounced growth retardation than the transgenic lines (Fig. 2c). In addition, after 2 weeks of growth on plates supplemented with 10 µM As(III) or 200 μM As(V), concentrations that did not cause severe growth inhibition, transgenic seedlings accumulated significantly less total arsenic than wild-type plants (Fig. 2d and e). These findings indicate that heterologous expression of MpACR3 enhances arsenic tolerance in Arabidopsis.

### Delayed Golgi-to-PM trafficking of MpACR3 in Arabidopsis under arsenic-free conditions

The subcellular localization of MpACR3-GFP in Arabidopsis was analyzed in root rhizodermal cells. MpACR3-GFP fluorescence was detected at the cell periphery, where it overlapped with FM4-64 signal (Fig. 2f). FM4-64 is commonly used to trace the endocytic pathway in root cells and can also serve as a PM marker under short-pulse staining conditions before substantial dye internalization (Rigal et al. 2015). Additionally, MpACR3-GFP was observed in distinct cytoplasmic compartments of varying sizes (Fig. 2f). Notably, exposure to 25 μM As(III) or 1,000 μM As(V) led to the near disappearance of these intracellular signals, with MpACR3-GFP colocalizing predominantly to the FM4-64-stained PM (Fig. 2f).

To further investigate the intracellular localization of MpACR3, *_pro_35S:*Mp*ACR3-GFP* transgenic plants were crossed with well-characterized WAVE-mCherry reporter lines (Geldner et al. 2009) and an HDEL-mCherry ER marker line (Nelson et al. 2007). Confocal microscopy revealed that MpACR3-GFP colocalized with Golgi markers, WAVE line 22R and WAVE line 127R, and with the PM marker WAVE line 138R, and partially overlapped with the ER marker HDEL-mCherry (Fig. 2f). By contrast, MpACR3-GFP did not colocalize with the vacuolar marker WAVE line 9R (Fig. 2f). We therefore conclude that MpACR3-GFP localizes predominantly to Golgi bodies and the PM under control conditions, although a minor ER-associated pool cannot be excluded. Such a pool may represent newly synthesized MpACR3 entering the secretory pathway or a transient trafficking intermediate rather than stable ER localization.

Upon exposure to 25 μM As(III) or 1,000 μM As(V), MpACR3-GFP no longer colocalized with the Golgi compartments in root cells (Fig. 2f). This indicates that, under control conditions, a substantial fraction of MpACR3-GFP accumulates in Golgi bodies rather than at the PM. In the presence of As(III) or As(V), however, Golgi-associated signal decreases and MpACR3-GFP accumulates predominantly at the PM. This redistribution was further confirmed by Lattice Lightsheet microscopy (Fig. 2g; Figure S7).

To refine the suborganellar localization of MpACR3-GFP within Golgi bodies, we transiently coexpressed MpACR3-GFP with markers for different Golgi compartments in tobacco leaf epidermal cells and analyzed their localization using high-resolution confocal microscopy (McGinness et al. 2022, 2025; Hawes et al. 2024). The intracellular MpACR3-GFP signal was associated with Golgi bodies and colocalized with the trans-Golgi marker ST-mRFP (Fig. 3a and c). By contrast, it did not overlap with the cis-Golgi marker MNS1-mRFP (Fig. 3a and c) or the trans-Golgi network (TGN) marker mRFP-SYP61 (Fig. 3d). Importantly, previous studies have shown that Golgi/TGN localization patterns are generally conserved between tobacco and Arabidopsis systems (Johansen et al. 2009; Berson et al. 2014). In addition, the ST marker used here is a well-established trans-Golgi marker that has been extensively validated in both tobacco transient assays and Arabidopsis cells (Brandizzi et al. 2002; Saint-Jore-Dupas et al. 2006). Together, these findings suggest that, under control conditions, MpACR3 is primarily retained in the trans-Golgi compartment in tobacco leaf epidermal cells. The Golgi-associated MpACR3-GFP signal observed in Arabidopsis may likewise correspond predominantly to the trans-Golgi, although direct confirmation in Arabidopsis root cells would be required.

**Figure 3:**
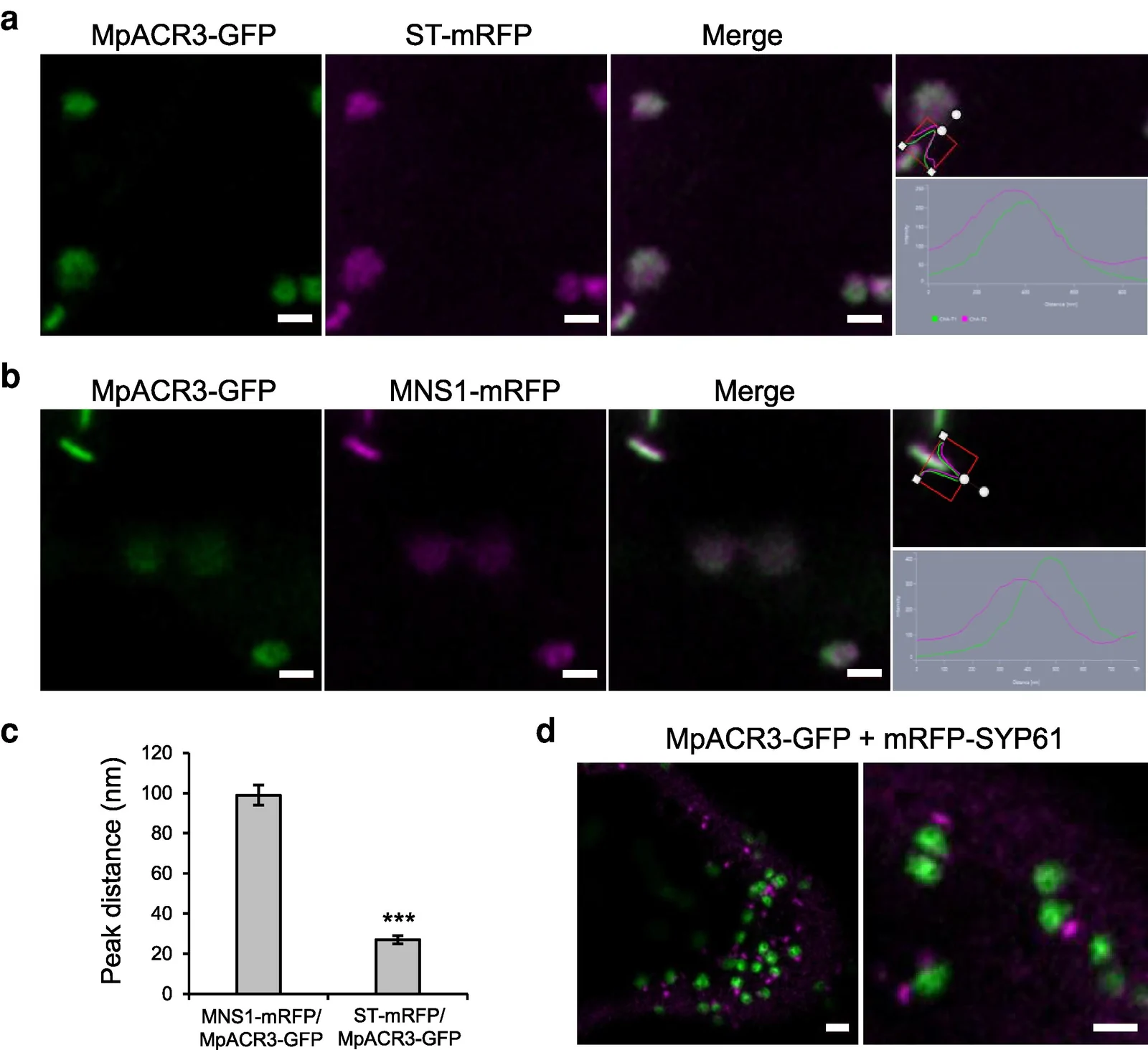
MpACR3-GFP associates predominantly with the trans-Golgi in tobacco leaf epidermal cells. a) Example images and line profile for MpACR3-GFP with the trans-Golgi body marker ST-mRFP and b) with the cis-Golgi body marker MNS1-mRFP. Line profile applied and line profile output are shown. Scale bars = 1 µm. c) Peak distance analysis graphs showing the markers MpACR3-GFP with MNS1-mRFP and ST-mRFP, respectively. Significance was analyzed by Kruskal–Wallis (***, *P*-value < 0.001). Data are from n = 3 biological replicates, each with at least 6 technical repeats. For comparison, the peak distance difference between MNS1-GFP with MNS1-mRFP is 18 ± 8 nm and MNS1-GFP with ST-mRFP is 122 ± 12 nm (McGinness et al. 2025). d) MpACR3-GFP was also coexpressed with the TGN marker mRFP-SYP61. Tobacco leaf epidermal cells transiently coexpressing the indicated proteins with fluorescent tags were visualized by Airyscan high-resolution confocal microscopy 3 d after infiltration. Example images are shown. Scale bars = 2 µm.

### Overexpression of MpACR3 increases arsenic tolerance in Marchantia

To investigate the function and subcellular localization of MpACR3 protein in its native organism, we generated 3 independent Marchantia transgenic lines expressing the *_pro_2* × *35S:*Mp*ACR3-mVenus* transgene, MpACR3-1, MpACR3-2, and MpACR3-3, in the wild-type Cam-1 background. We first examined MpACR3-mVenus protein levels in plants grown with and without metalloids. Similar to MpACR3-GFP in Arabidopsis, MpACR3-mVenus protein levels increased upon exposure to As(III) and As(V) (Fig. 4a; Figure S8a). To determine whether the increase in MpACR3-mVenus protein abundance was accompanied by an increase in transcript levels, we quantified Mp*ACR3-mVenus* mRNA levels by qPCR using primers specific to the *mVenus* region. Transcript levels were higher in arsenic-treated plants than in controls (Fig. 4b), indicating that the increase in MpACR3-mVenus protein abundance was accompanied by increased transcript accumulation. Whether this resulted from altered CaMV 35S promoter activity or transcript stability was not determined. These lines were used primarily as localization reporters to assess the subcellular distribution of MpACR3 and its response to As(III), rather than to infer transcriptional regulation from changes in protein abundance.

**Figure 4:**
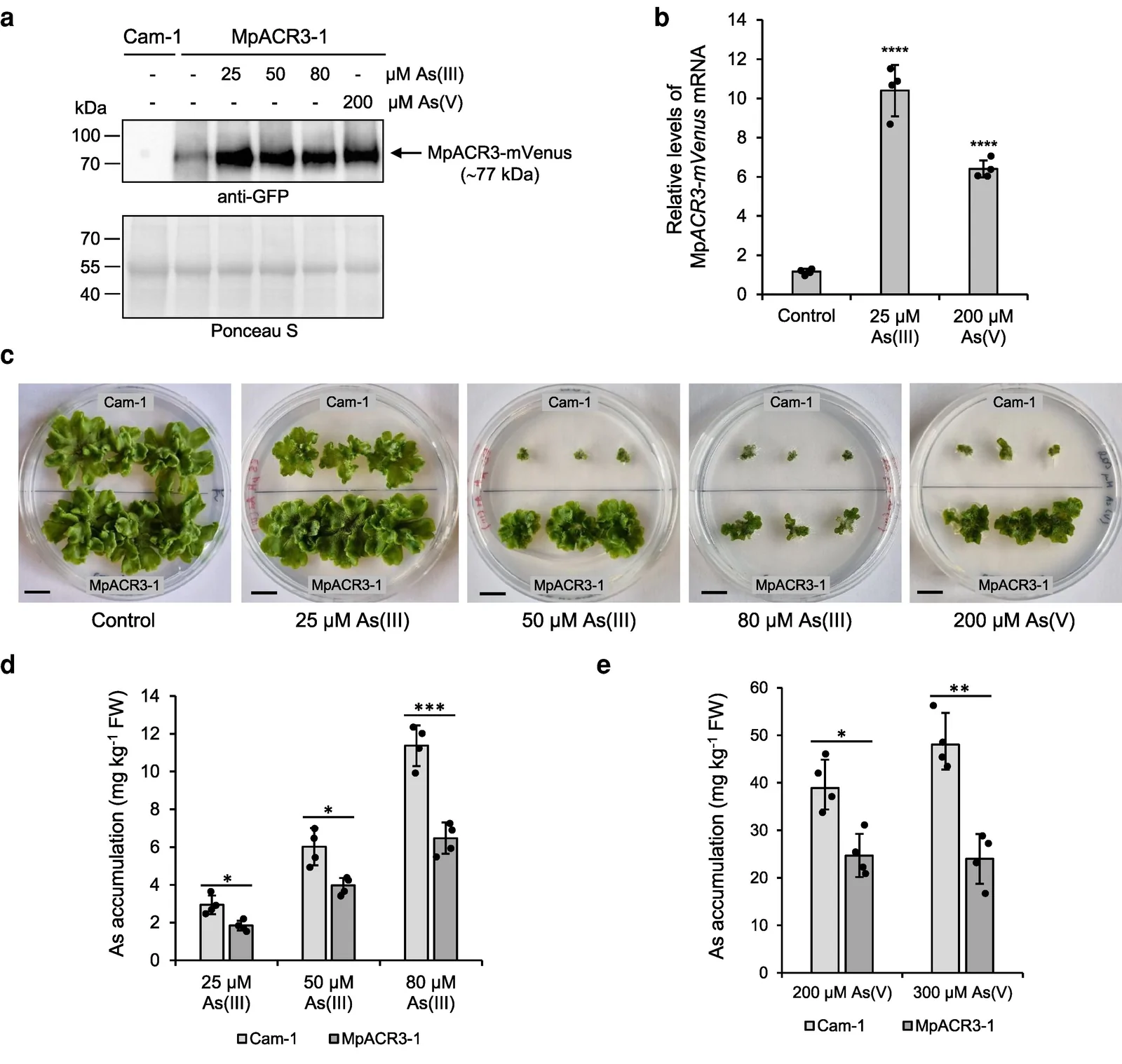
Overexpression of Mp*ACR3-mVenus* confers increased tolerance to arsenic in Marchantia. a) Western blot analysis of MpACR3-mVenus protein levels in Marchantia transgenic line MpACR3-1 expressing the *_pro_2* × *35S:*Mp*ACR3-mVenus* transgene grown in the presence of indicated concentrations of As(III) and As(V) for 3 weeks or under control conditions. Protein extract from wild-type Marchantia (Cam-1) was used as a negative control. Ponceau S staining served as a protein loading control. b) *_pro_2* × *35S:*Mp*ACR3-mVenus* transcript abundance in the transgenic MpACR3-1 line grown on solid medium in the absence (control) or presence of indicated concentrations of As(III) and As(V) for 2 weeks. Mp*APT* (adenine phosphoribosyltransferase) was used as a reference gene to normalize expression data of Mp*ACR3-mVenus*. Data are presented as mean ± SD from 4 independent experiments; each dot represents one biological replicate consisting of pooled thallus tissue from an independent experiment. Statistical significance was assessed using one-way ANOVA followed by Dunnett's multiple comparison test against the untreated control (****, *P*-value < 0.0001). c) Comparative growth of the wild-type Cam-1 and the transgenic line MpACR3-1 grown in the absence or presence of the indicated concentrations of As(III) and As(V) for 3 weeks. Scale bars = 1 cm. d, e) Arsenic accumulation in Cam-1 and MpACR3-1 gametophytes grown in the presence of indicated concentrations of As(III) or As(V) for 3 weeks. For (d) and (e), data are presented as mean ± SD from 4 independent experiments; each dot represents one biological replicate consisting of pooled thallus tissue from an independent experiment. Statistical significance was assessed using two-way ANOVA followed by planned pairwise comparisons between Cam-1 and MpACR3-1 at each As(III)/As(V) concentration using Welch's *t*-test with Holm correction. Asterisks indicate significant differences between genotypes within each treatment: * *P*-value < 0.05, **P-value < 0.01, ***P-value < 0.001.

Furthermore, Marchantia lines overexpressing Mp*ACR3-mVenus* exhibited increased tolerance to As(III) and As(V) (Fig. 4c; Figure S8b) and accumulated less arsenic than wild-type plants (Fig. 4d and e; Figure S8c and d). These findings show that MpACR3 overexpression enhances arsenic tolerance and reduces arsenic accumulation in Marchantia, consistent with its proposed role in As(III) efflux.

### Arsenic induces relocalization of MpACR3 from Golgi bodies to the PM in Marchantia

To further investigate MpACR3 localization in Marchantia, we examined MpACR3-mVenus in gemmae by confocal microscopy in the absence or presence of As(III). Under control conditions, MpACR3-mVenus fluorescence was detected both at the cell periphery and in intracellular punctate structures. Following As(III) exposure, the intracellular signal was largely lost, and MpACR3-mVenus accumulated predominantly at the cell periphery, consistent with PM localization (Fig. 5a). Using 100 μM As(III) and time-lapse confocal microscopy, we found that arsenic-driven relocalization of MpACR3-mVenus from Golgi bodies to the PM is rapid, with Golgi retention disappearing within 10 min of As(III) addition (Figure S9 and Video S1).

**Figure 5:**
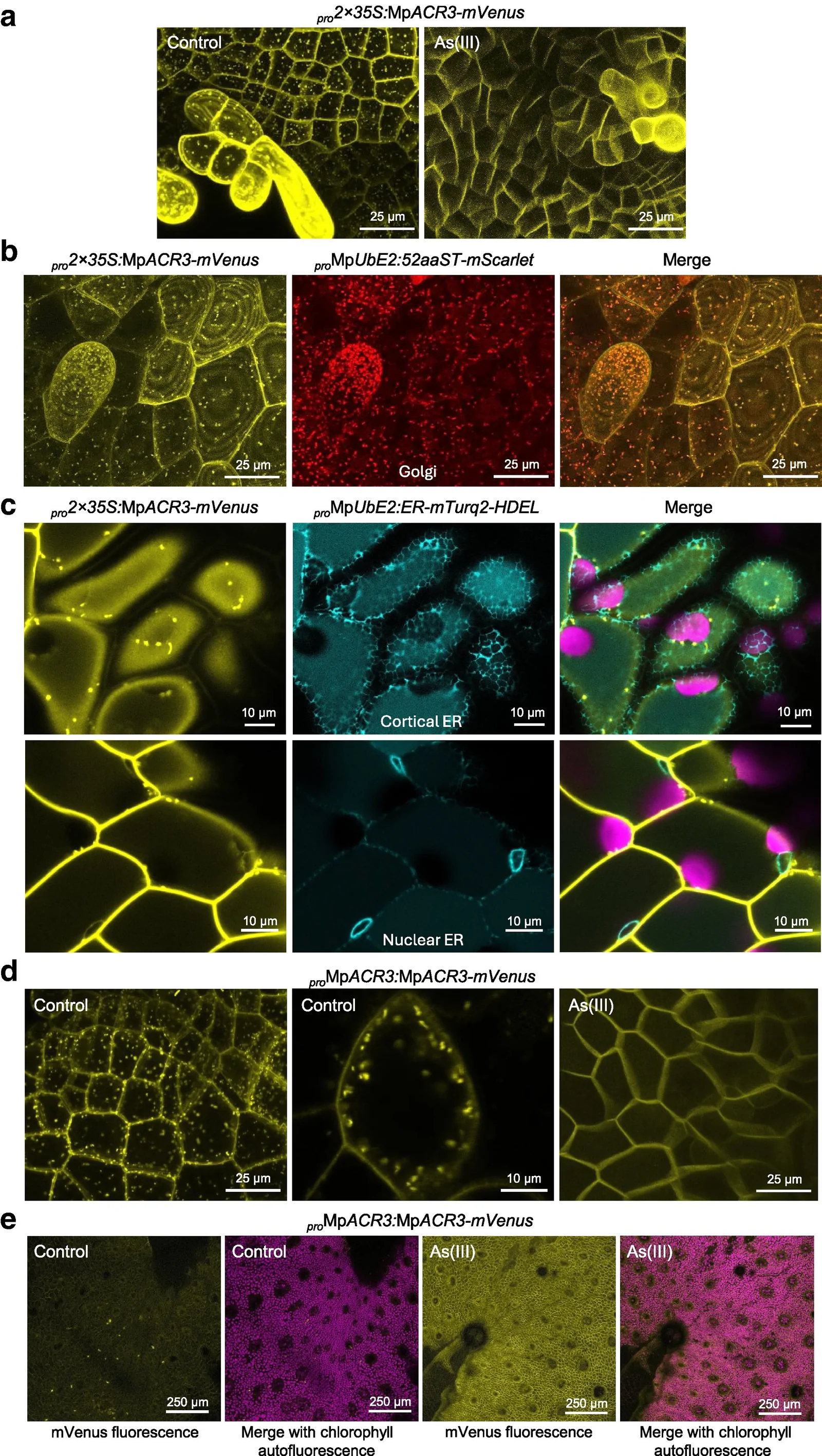
Arsenic induces MpACR3 redistribution from intracellular compartments to the plasma membrane in Marchantia. a) The MpACR3-mVenus yellow fluorescence was detected at the cell periphery and in intracellular punctate structures under control conditions. After exposure to 25 μM As(III) for 24 h, the punctate signal was largely lost and MpACR3-mVenus accumulated predominantly at the cell periphery, consistent with PM localization. The Marchantia gemmae of the MpACR3-1 line expressing *_pro_2* × *35S:*Mp*ACR3-mVenus* were plated on solid 0.5× Gamborg B5 medium with or without 25 μM As(III), and imaged by confocal microscopy 24 h after treatment. b) MpACR3-mVenus colocalizes with Golgi bodies under control conditions. Colocalization of MpACR3-mVenus (yellow) with Golgi bodies was analyzed in transgenic Marchantia sporelings expressing *_pro_*Mp*UbE2:52aaST-mScarlet*, which encodes a Golgi marker consisting of the transmembrane domain of rat sialyltransferase (ST) fused to the red fluorescent protein mScarlet (ST-mScarlet). c) MpACR3-mVenus does not colocalize with the cortical ER network but shows limited spatial overlap with the perinuclear ER signal under control conditions. Colocalization of MpACR3-mVenus with the ER (cyan) was analyzed in the transgenic Marchantia gemmae expressing *_pro_*Mp*UbE2:ER-mTurq2-HDEL*, which encodes mTurquoise2 fused to an N-terminal ER-targeting signal peptide and a C-terminal ER retention signal, HDEL. Merged images also show chlorophyll autofluorescence in magenta. d) MpACR3-mVenus (yellow) expressed from its native *_pro_*Mp*ACR3:*Mp*ACR3-mVenus* line also showed peripheral and intracellular punctate localization under control conditions and accumulated predominantly at the PM in gemmae in response to As(III) treatment. The punctate structures are consistent with the Golgi localization identified in (b). A higher-magnification view of a single cell is shown in the middle image. e) Gemmae expressing *_pro_*Mp*ACR3:*Mp*ACR3-mVenus* showed a marked increase in the mVenus fluorescence signal in response to As(III) exposure. For (d) and (e), the *_pro_*Mp*ACR3:*Mp*ACR3-mVenus* Marchantia line was plated on solid 0.5× Gamborg B5 medium with or without 25 μM As(III), and sporelings (d) or gemmae (e) were imaged by confocal microscopy 24 h after treatment. Images compared within each experiment were acquired using identical microscope settings. Images are representative of 2 independent experiments; at least 3 gemmae or sporelings and 2 fields of view were examined per condition. Scale bars are indicated in the images.

Because MpACR3 localizes to Golgi bodies in Arabidopsis and tobacco cells (Figs. 2 and 3), we hypothesized that the punctate intracellular structures observed in Marchantia also correspond to Golgi bodies. To test this, we generated a transgenic line expressing *_pro_2* × *35S:*Mp*ACR3-mVenus* and the Golgi marker *_pro_*Mp*UbE2:52aaST-mScarlet*. The intracellular MpACR3-mVenus signal colocalized with ST-mScarlet-labeled Golgi bodies (Fig. 5b), confirming its Golgi localization in Marchantia.

Because MpACR3 was detected in the ER in yeast cells (Fig. 1d and e) and partially colocalized with the ER marker HDEL-mCherry in Arabidopsis root cells (Fig. 2f), we next asked whether MpACR3 is also associated with the ER in Marchantia. To this end, we generated a transgenic line expressing *_pro_2* × *35S:*Mp*ACR3-mVenus* together with the ER marker *_pro_*Mp*UbE2:ER-mTurq2-HDEL*. We did not detect colocalization of MpACR3-mVenus with the cortical ER network, although a weak overlap with the nuclear ER signal was observed (Fig. 5c). These observations support the conclusion that MpACR3 is not predominantly retained in the ER in Marchantia under control conditions, although a small ER-associated fraction may be present.

In the experiments described above, MpACR3-mVenus was expressed from the strong constitutive 2 × 35S promoter, raising the possibility that intracellular retention of MpACR3 might result from protein overproduction. In addition, CaMV 35S-driven expression may vary depending on plant species, tissue, developmental stage, and environmental conditions (Kiselev et al. 2021; Tse et al. 2024). To address this, we expressed Mp*ACR3-mVenus* under the control of its native promoter. In the resulting transgenic line, MpACR3-mVenus still showed dual Golgi/PM localization under control conditions and accumulated predominantly at the PM after As(III) treatment (Fig. 5d). We also observed a marked increase in MpACR3-mVenus fluorescence signal in response to As(III) (Fig. 5e). Consistent with this observation, MpACR3 transcript levels were strongly upregulated in wild-type Marchantia exposed to arsenic (Figure S10), suggesting that increased MpACR3 expression provides an additional layer of regulation contributing to arsenic detoxification.

Together, these results indicate that, in plant cells, MpACR3 localizes to Golgi bodies and the PM under control conditions, with a minor fraction associated with the ER. In response to arsenic stress, MpACR3 is released from intracellular retention and accumulates predominantly at the PM. This arsenic-induced relocalization, together with transcriptional upregulation of MpACR3, likely enhances the arsenic detoxification capacity in Marchantia.

### MpACR3 accumulation at the PM is triggered by environmentally relevant As(III) concentrations

Arsenic concentrations in uncontaminated freshwater are typically below 10 µg L^−1^ (<0.133 µM) and often below 1 µg L^−1^ (<0.013 µM), whereas contaminated groundwaters may exceed 50 µg L^−1^ (>0.667 µM) and can reach >5,000 µg L^−1^ (>66.7 µM) in severely affected areas (Smedley and Kinniburgh 2002). Wenzel et al. (2002) measured arsenic concentrations in soil solutions from contaminated soils and reported 0–171 µg L^−1^, equivalent to 0–2.28 µM. A recent paddy-soil study reported porewater arsenic concentrations of 2.97–14.85 µg L^−1^ during rice growth, equivalent to 0.040–0.198 µM (Kong et al. 2024).

To test whether relocalization of MpACR3 from intracellular compartments to the PM is triggered by environmentally relevant arsenic concentrations, we examined MpACR3 localization in Arabidopsis and Marchantia treated with 0.01–5 µM As(III) for 24 h (Fig. 6a and b). The lowest As(III) concentrations that induced MpACR3 accumulation at the PM were 0.1 µM in Arabidopsis (Fig. 6a) and 0.5 µM in Marchantia (Fig. 6b). Notably, residual Golgi-associated MpACR3 signal was still observed in some regions of the Arabidopsis root, likely reflecting uneven As(III) distribution within the root (Fig. 6a; bottom images). These results indicate that MpACR3 trafficking responds to environmentally relevant As(III) concentrations, supporting the conclusion that As(III)-induced relocalization from Golgi bodies to the PM is not an artifact of exposure to excessively high arsenic concentrations.

**Figure 6:**
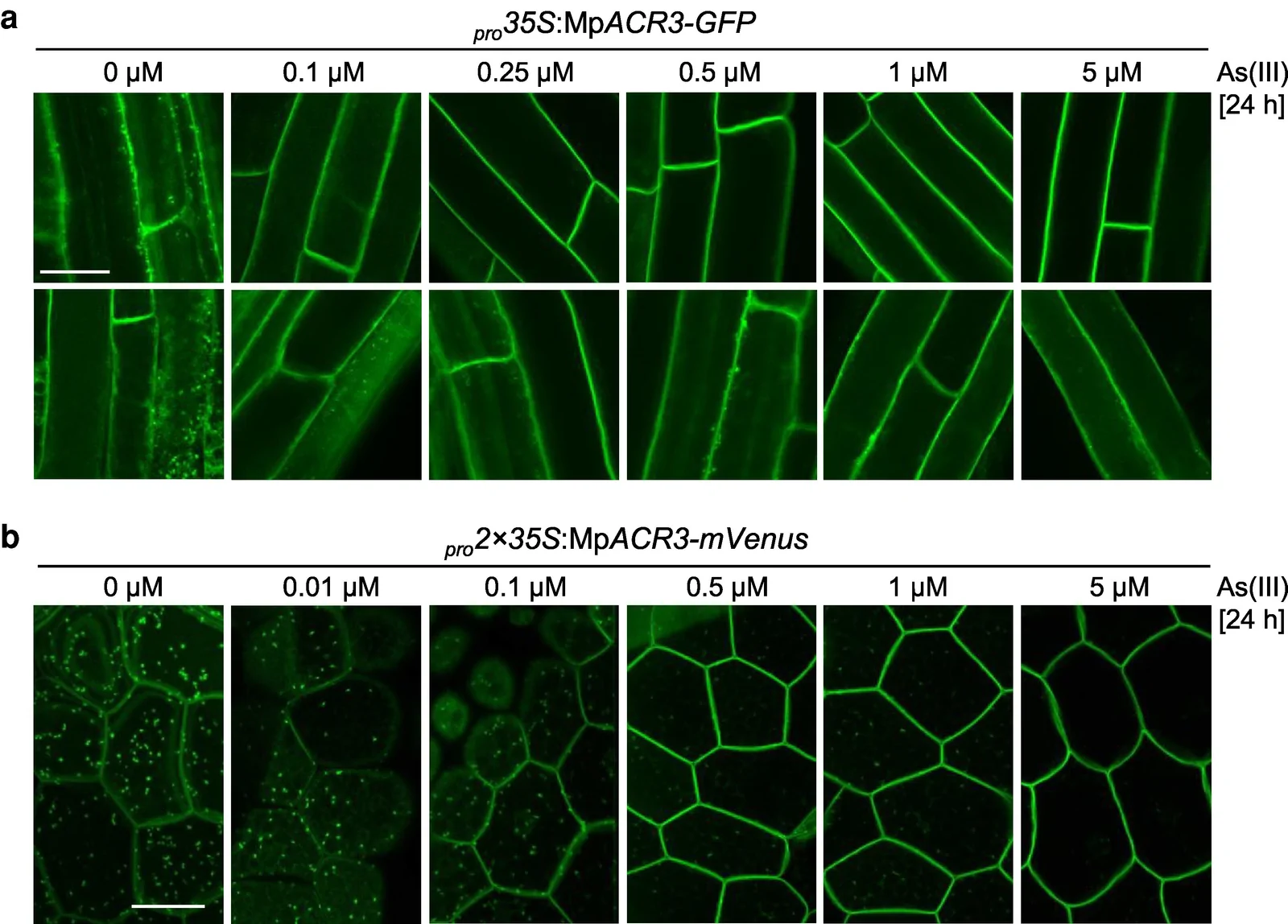
Relocalization of MpACR3 from intracellular membranes to the PM is triggered by environmentally relevant concentrations of As(III). a) Arabidopsis transgenic seedlings expressing *_pro_35S:*Mp*ACR3-GFP*, line T27, were incubated in the presence of the indicated As(III) concentrations for 24 h and then root cells were analyzed with confocal microscopy. b) Marchantia gemmae expressing the *_pro_2* × *35S:*Mp*ACR3-mVenus* transgene (MpACR3-1 line) were exposed to the indicated concentrations of As(III) for 24 h and then analyzed by confocal microscopy. Images are representative of 3 gemmae; 2 fields of view were examined per condition. Scale bars = 5 µm.

### Regulation of MpACR3 trafficking by its N-terminal cysteine-rich domain

The predicted structure of MpACR3 features a large cytosolic N-terminal domain comprising 123 amino acid residues (Figures S2 and S3), including 5 cysteine residues (Fig. 7a). Importantly, C29, C47, and C71 are highly conserved in plant ACR3 transporters with extended N-terminal tails of 104–163 residues (Fig. 7a; Figure S3). By contrast, ACR3 sequences from bacteria, fungi, and certain plants, primarily chlorophytes and ferns, have much shorter N-terminal tails, typically fewer than 100 residues, that are often not enriched in cysteine residues (Fig. 7a; Figures S1 and S4). Proteins involved in arsenic detoxification are frequently regulated through the coordination of As(III) by 3 closely spaced cysteine residues (Shi et al. 1996; Kumar et al. 2015; Lee and Levin 2022). We therefore hypothesized that the N-terminal cysteine-rich domain modulates MpACR3 trafficking by sensing cytoplasmic As(III).

**Figure 7:**
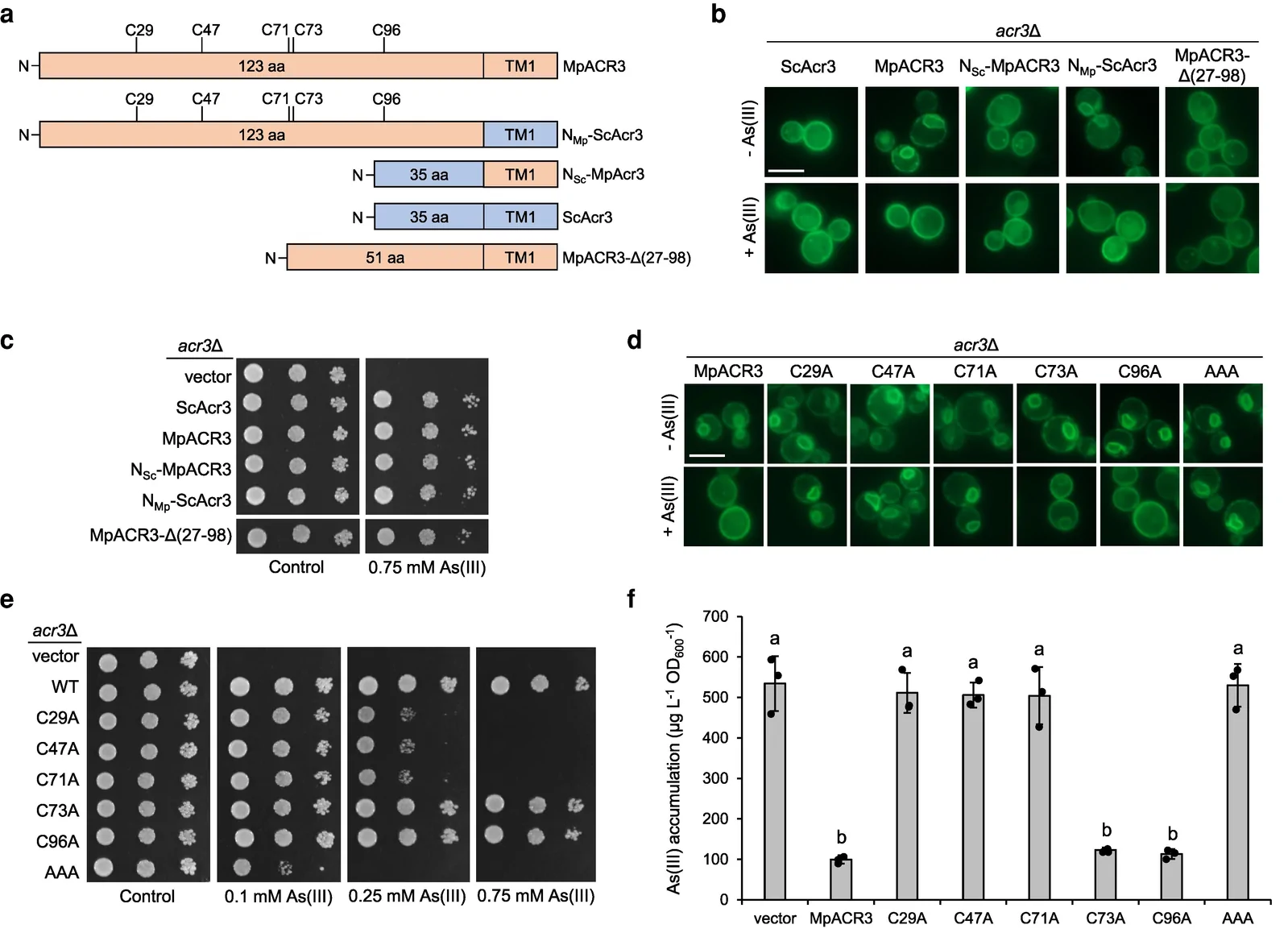
The N-terminal cysteine-rich domain regulates As(III)-induced relocalization of MpACR3 from the ER to the PM in yeast cells. a) Schematic representation of the N-terminal tails of ScAcr3, MpACR3, the N-terminal tail-swap variants, N_Sc_-MpACR3 and N_Mp_-ScAcr3, and the MpACR3-Δ27-98 variant lacking the cysteine-rich fragment within the N-terminal tail. The positions of cysteine residues and the first transmembrane span (TM1) are indicated. b) The N-terminal cysteine-rich region determines ER retention of MpACR3 in the absence of As(III) in yeast cells. The *S. cerevisiae acr3*Δ strain was transformed with plasmids expressing GFP-tagged ScACR3, MpACR3, and the indicated variants. Localization of GFP-tagged proteins was analyzed under control conditions or after exposure to 0.1 mM As(III) for 2 h by fluorescence microscopy. Scale bars = 5 μm. c) Tail-swap variants and the MpACR3-Δ27-98 variant confer As(III) resistance comparable to wild-type MpACR3. d) C29, C47, and C71 are required for As(III)-induced relocalization of MpACR3 from the ER to the PM. Subcellular localization of the indicated variants of MpACR3, including single cysteine-to-alanine variants and the triple-alanine variant C29A C47A C71A (AAA), was analyzed in *acr3*Δ cells by fluorescence microscopy. The AAA variant was also analyzed in the Δ-s-tether strain. Yeast transformants were left untreated or exposed to 0.1 mM As(III) for 2 h. Scale bars = 5 μm. e) Transformants expressing the indicated cysteine-to-alanine MpACR3 variants, which are defective in As(III)-induced relocalization from the ER to the PM, showed reduced tolerance to As(III) compared with wild-type MpACR3 (WT). f) The C29A, C47A, C71A, and AAA variants show reduced ability to limit intracellular As(III) accumulation. Intracellular accumulation of As(III) in the indicated *acr3*Δ transformants was measured after exposure to 1 mM As(III) for 2 h using atomic absorption spectrometry. Data are presented as mean ± SD from 3 independent experiments for each transformant. Statistical significance was assessed using Welch's one-way ANOVA followed by Games-Howell post hoc comparisons. Different letters indicate statistically significant differences (*P*-value < 0.05).

To investigate the role of the N-terminal cysteine-rich domain in MpACR3 localization, we engineered plasmids encoding N-terminal swap variants: MpACR3-GFP carrying the yeast ACR3 N-terminal region (N_Sc_-MpACR3-GFP) and ScACR3-GFP carrying the Marchantia ACR3 N-terminal region (N_Mp_-ScACR3-GFP) (Fig. 7a). We also generated a truncated MpACR3 variant lacking the cysteine-rich region (MpACR3-Δ27-98-GFP). These constructs were heterologously expressed in yeast *acr3*Δ cells. In the absence of As(III), both N_Sc_-MpACR3-GFP and MpACR3-Δ27-98-GFP localized exclusively to the PM, similar to ScACR3, and were no longer retained in the ER. Conversely, N_Mp_-ScACR3-GFP was retained in the ER under control conditions but relocalized to the PM upon As(III) exposure (Fig. 7b). Despite these differences in localization, all tested variants (N_Sc_-MpACR3-GFP, N_Mp_-ScACR3-GFP, and MpACR3-Δ27-98-GFP) conferred As(III) tolerance comparable to full-length MpACR3-GFP, confirming that they remained functional (Fig. 7c). Collectively, these findings demonstrate that the N-terminal cysteine-rich domain controls intracellular retention and As(III)-responsive trafficking in yeast.

To determine which cysteine residues in the N-terminal domain are required for MpACR3 trafficking, potentially through direct As(III) binding, we individually substituted each cysteine with alanine, generating the C29A, C47A, C71A, C73A, and C96A variants. Under control conditions, all MpACR3 variants localized to the ER and PM, similar to wild-type MpACR3 (Fig. 7d). Upon As(III) exposure, however, the C29A, C47A, and C71A variants failed to relocalize from the ER to the PM (Fig. 7d). These variants also showed significantly reduced As(III) resistance compared with wild-type MpACR3 (Fig. 7e) and failed to decrease As(III) accumulation in yeast cells (Fig. 7f). The C73A variant showed a mild defect in As(III)-induced relocalization (Fig. 7d) but retained wild-type As(III) tolerance (Fig. 7e and f). By contrast, the C96A variant showed no detectable abnormalities in any assay.

To further investigate the combined contribution of multiple cysteine residues, we generated a triple cysteine-to-alanine mutant, C29A C47A C71A, hereafter referred to as AAA, and expressed it in *acr3*Δ cells as well as in the Δ-s-tether strain, which shows ER-PM separation. Similar to the single-cysteine variants, the AAA mutant localized primarily to the ER, with only a fraction reaching the PM, irrespective of As(III) exposure (Fig. 7d). Notably, *acr3*Δ cells expressing the AAA variant were more sensitive to As(III) than those expressing the single-cysteine mutants (Fig. 7e), suggesting that combined substitution of these cysteine residues further compromises MpACR3-dependent arsenic detoxification. Overall, these findings strongly indicate that C29, C47, and C71 are required for efficient As(III)-induced MpACR3 trafficking to the PM.

### The N-terminal domain of MpACR3 functions as an intracellular arsenic sensor

To determine whether As(III) directly binds to the N-terminal domain of MpACR3, we expressed a 1-123-amino-acid N-terminal fragment fused to a 7×HA tag (7HA-MpACR3-N-tail) in *acr3*Δ cells, together with the AAA (C29A C47A C71A) and Cys-null (C29A C47A C71A C73A C96A) variants. Protein extracts from these cells were either treated with As(III) for 60 min or left untreated, followed by incubation with an arsenite-biotin conjugate probe. Samples were then subjected to pulldown using streptavidin-agarose resins and analyzed by Western blotting with anti-HA antibodies. The arsenite-biotin conjugate bound specifically to the N-terminal domain of wild-type MpACR3 but not detectably to the AAA or Cys-null variants (Fig. 8a). Furthermore, pretreatment of protein extracts with As(III) abolished arsenite-biotin binding to the MpACR3 N-terminus (Fig. 8a). These findings strongly suggest that As(III) directly interacts with the N-terminal domain of MpACR3 in a manner dependent on C29, C47, and C71, consistent with the proposed role of this domain as an intracellular arsenic sensor.

**Figure 8:**
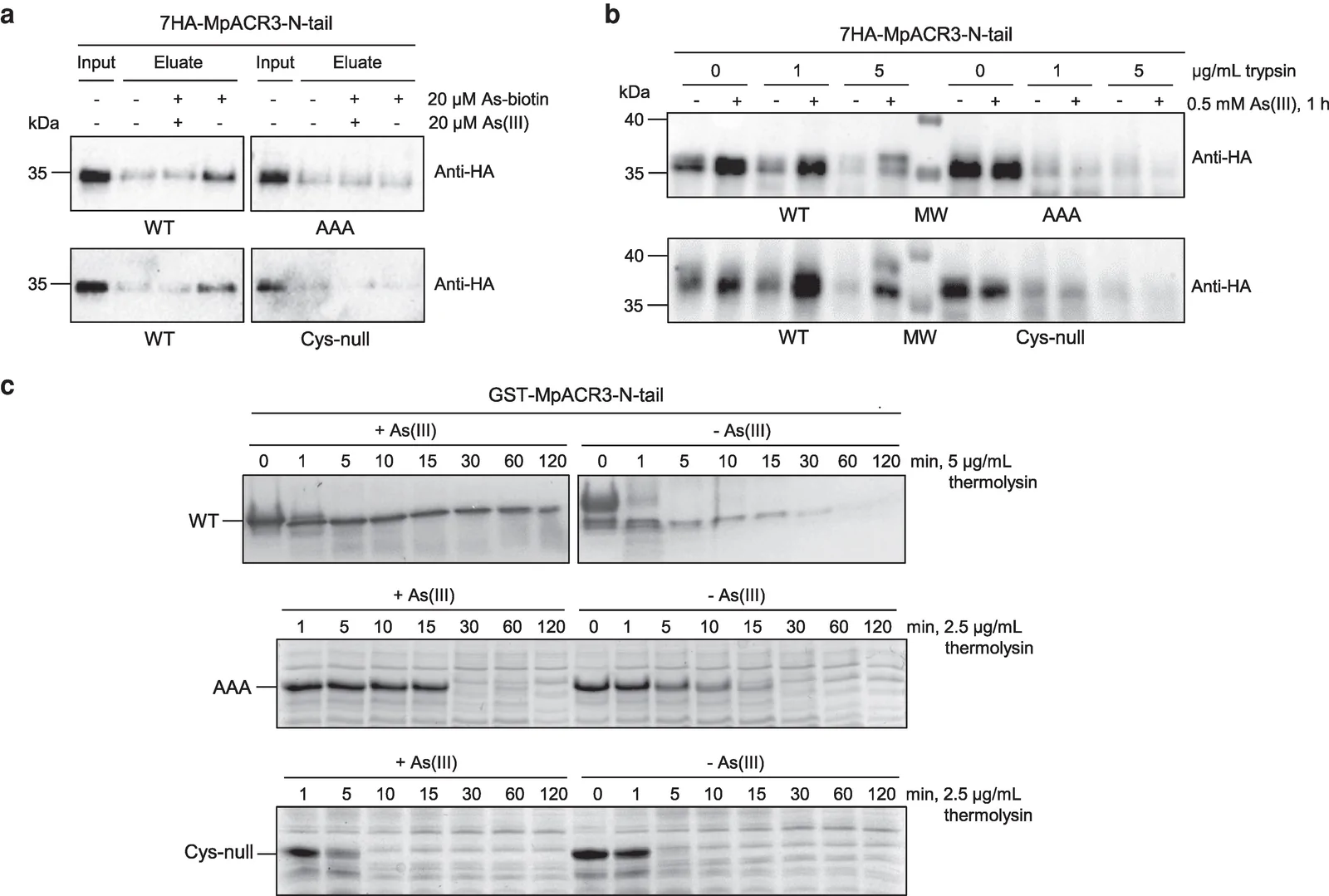
The N-terminal cysteine-rich domain of MpACR3 functions as an arsenic-sensing module. a) The As-biotin conjugate binds to the N-terminal cysteine-rich domain of MpACR3. Protein lysates were prepared from *acr3*Δ cells expressing the indicated HA-tagged MpACR3 N-terminal domain variants: wild-type, WT; C29A C47A C71A, AAA; or C29A C47A C71A C73A C96A, Cys-null. Protein lysates were first pre-incubated with 20 μM As(III) or left untreated and then incubated with 20 μM As-biotin conjugate. Proteins bound to the As-biotin conjugate were recovered using streptavidin-agarose resin and analyzed by Western blotting with anti-HA antibodies. b, c) As(III) binding to the MpACR3 N-terminal cysteine-rich domain induces conformational changes both in vivo and in vitro. (b) As(III) exposure alters the trypsin susceptibility of the MpACR3 N-terminal domain. Lysates were prepared from *acr3*Δ cells expressing the indicated variants of the MpACR3 N-terminal domain after treatment with 0.5 mM As(III) for 60 min or under control conditions. Lysates were then exposed to increasing concentrations of trypsin, followed by Western blot analysis with anti-HA antibodies. Quantification from independent experiments is shown in Figure S11. MW, molecular weight marker. c) As(III) alters the thermolysin susceptibility of purified GST-MpACR3 N-terminal-domain proteins. GST-fused protein variants of the MpACR3 N-terminal domain were purified from *Escherichia coli* lysates by affinity purification using glutathione-Sepharose columns. Purified GST fusion proteins were treated with 20 μM As(III) or left untreated and then subjected to a time course of limited thermolysin digestion. Because the cysteine-deficient variants were more susceptible to proteolysis, WT was analyzed using 5 μg/mL thermolysin, whereas AAA and Cys-null were analyzed using 2.5 μg/mL thermolysin. Proteolysis patterns were visualized by SDS-PAGE followed by Coomassie staining.

### As(III) binding induces a conformational change in the MpACR3 N-terminal domain

Ligand binding can markedly influence the susceptibility of a protein segment to limited proteolysis. For example, As(III) binding induces a conformational change in the yeast transcription factor ScYap8, reducing its susceptibility to trypsin digestion and enabling activation of *ScACR3* transcription (Kumar et al. 2015). Based on this, we hypothesized that As(III) binding to the MpACR3 N-terminal domain induces a structural change that influences its intracellular retention.

To test this, we prepared protein extracts from yeast cells expressing wild-type 7HA-MpACR3-N-tail or its cysteine-deficient variants. These extracts were either treated with As(III) or left untreated before incubation with increasing concentrations of trypsin. Proteolysis patterns were analyzed by anti-HA immunoblotting (Fig. 8b; Figure S11). As(III) exposure increased the resistance of wild-type 7HA-MpACR3-N-tail to trypsin digestion compared with untreated samples. By contrast, both the AAA and Cys-null variants remained highly susceptible to proteolysis, irrespective of As(III) treatment. Notably, even in the absence of As(III), wild-type 7HA-MpACR3-N-tail exhibited slightly greater resistance to trypsin digestion than its cysteine-deficient counterparts. These findings suggest that As(III) binding stabilizes the MpACR3 N-terminal domain, triggering a conformational change that may regulate its intracellular localization.

To further investigate the structural effects of As(III) binding, we performed a protease resistance-based protein stability assay using recombinantly expressed and purified GST-MpACR3-N-tail and its cysteine-deficient variants. These proteins were incubated with thermolysin, a nonspecific endoproteinase, in the presence or absence of As(III). In the ligand-free state, GST-MpACR3-N-tail was highly susceptible to thermolysin digestion. Upon As(III) binding, however, it became markedly resistant to proteolysis, even after extended incubation (Fig. 8c). Because substrate binding in the protease active site requires an extended conformation (Tyndall et al. 2005), these findings suggest that As(III) binding induces a stable, highly ordered fold in the MpACR3 N-terminal domain.

As expected, the 2 cysteine-deficient variants, GST-MpACR3-AAA-N-tail and GST-MpACR3-Cys-null-N-tail, were more readily degraded than wild-type GST-MpACR3-N-tail. However, in the presence of As(III), GST-MpACR3-AAA-N-tail showed slightly increased resistance to thermolysin digestion compared with its ligand-free state, suggesting that, at least in vitro, As(III) may also interact with C73 and C96. Despite the use of a lower protease concentration, the Cys-null variant remained highly susceptible to rapid and complete degradation, irrespective of As(III) treatment (Fig. 8c). This accelerated proteolysis suggests that the cysteine residues required for As(III) interaction may not only mediate As(III) binding but also contribute to the structural stability of the MpACR3 N-terminal domain. Taken together, these results support a role for the MpACR3 N-terminal domain as an intracellular arsenic sensor and are consistent with As(III)-dependent conformational remodeling involving C29, C47, and C71, which may influence MpACR3 intracellular retention.

### Identification of an arginine-based sorting signal in the MpACR3 N-terminal domain

To elucidate the molecular mechanisms governing MpACR3 intracellular localization, we analyzed its N-terminal domain for known sequence motifs critical for ER or Golgi retention (Michelsen et al. 2005; Gao et al. 2014; Lujan and Campelo 2021). This analysis revealed an RXR-type arginine-based sorting signal, where X represents any amino acid, within the highly conserved 45-LRCRF-49 sequence, with the As(III)-responsive C47 residue positioned centrally (Fig. 9a; Figure S3). Arginine-based sorting signals play key roles in the retention of ER and Golgi membrane proteins and are widely conserved among eukaryotes (Michelsen et al. 2005; Banfield 2011; Lujan and Campelo 2021). In addition, functional sorting signals often contain a bulky hydrophobic residue, typically leucine, preceding an arginine cluster such as RR, RXR, or RRXR.

**Figure 9:**
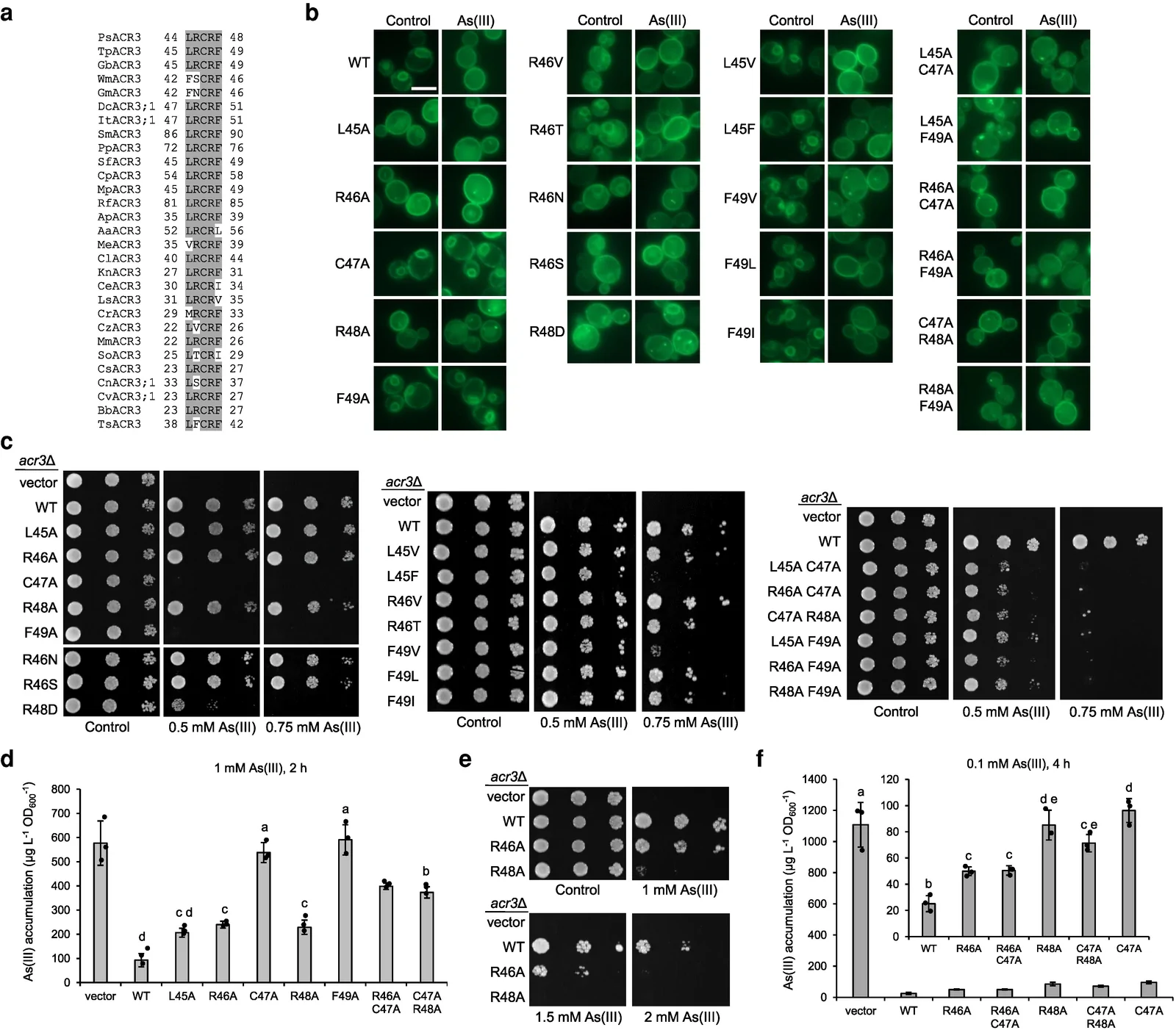
Functional analysis of the LRCRF motif in MpACR3 intracellular retention and arsenic detoxification. a) Multiple sequence alignment of the LRCRF motif in plant ACR3 proteins. Sequence details are provided in Table S1. b) Localization of MpACR3-GFP (WT) and its variants carrying substitutions in the LRCRF motif in *S. cerevisiae acr3*Δ cells under control conditions or after exposure to 0.1 mM As(III) for 2 h, visualized by fluorescence microscopy. Scale bar = 5 μm. c) As(III) sensitivity of *acr3*Δ transformants expressing MpACR3-GFP or LRCRF motif variants. d) Intracellular As(III) accumulation in *acr3*Δ cells expressing MpACR3-GFP or LRCRF motif variants after exposure to 1 mM As(III) for 2 h. Arsenic content was measured by atomic absorption spectrometry. e) Growth of *acr3*Δ cells expressing MpACR3-GFP or the R46A and R48A variants in the presence of the indicated As(III) concentrations. f) Intracellular As(III) accumulation in *acr3*Δ cells expressing MpACR3-GFP or LRCRF motif variants after exposure to 0.1 mM As(III). The inset shows the same data on an expanded y-axis to visualize differences among MpACR3 variants. For (d) and (f), bars represent mean ± SD from 3 independent experiments; individual data points are shown. Statistical significance was assessed using one-way ANOVA followed by Tukey's HSD post hoc test. Different letters indicate statistically significant differences among transformants at *P*-value < 0.05.

To determine the role of the LRCRF motif in MpACR3 intracellular retention, we performed alanine-scanning mutagenesis and analyzed the subcellular localization of the resulting variants in yeast cells. Microscopy revealed that the L45A, R46A, and R48A variants failed to localize to the ER in the absence of As(III), confirming that these residues are essential for MpACR3 retention under control conditions (Fig. 9b). By contrast, similar to C47A, the F49A variant remained ER-localized irrespective of As(III) presence. This suggests that F49 may play a structural role in maintaining the conformation required for As(III)-responsive trafficking, consistent with the f relocalize to the PM upon As(III) exposure (Fig. 9a). Accordingly, MpACR3 variants defective in ER retention retained the ability to confer As(III) tolerance. However, expression of the F49A variant failed to complement the As(III) sensitivity of the *acr3*Δ mutant (Fig. 9c), supporting a role for F49 in the As(III)-induced release of MpACR3 from intracellular retention.

Sequence alignment of plant ACR3 proteins revealed variation in the LRCRF region (Fig. 9a; Figure S3). To define the sequence requirements for plant ACR3 sorting, we generated a series of MpACR3 variants: L45F, L45 V, R46N, R46S, R46T, R46 V, F49I, F49L, and F49 V. Fluorescence microscopy showed that L45F, L45 V, F49I, and F49L variants retained strong GFP signals in the ER under control conditions, which disappeared upon As(III) exposure, resembling the behavior of wild-type MpACR3-GFP (Fig. 9b). By contrast, R46N, R46S, R46T, and R46 V variants showed impaired ER retention, as indicated by increased PM localization, manifested by a more uniform signal at the cell periphery rather than the patchy pattern typical of the cortical ER, the presence of punctate cytoplasmic structures, and GFP accumulation inside the vacuole, suggesting endocytic degradation of MpACR3-GFP (Fig. 9b). Importantly, despite these differences in localization, all tested variants remained functional, as confirmed by their ability to complement the As(III) sensitivity of the *acr3*Δ mutant (Fig. 9c). These findings indicate that L45 and F49 can be substituted with other bulky hydrophobic residues without affecting MpACR3-GFP trafficking. In contrast, replacement of R46 with asparagine, serine, threonine, or valine substantially weakened the ER retention signal, consistent with the key role for this arginine residue in retention motif function.

Given the high conservation of the second arginine residue within the LRCRF motif (Fig. 9a; Figure S3), we generated the R48D variant to investigate the functional importance of its positive charge. This substitution disrupted ER localization, resulting in a predominant GFP signal at the PM under both control and As(III)-treated conditions. Nonetheless, a fraction of MpACR3-R48D-GFP remained detectable in the ER and in punctate cytoplasmic structures, likely corresponding to Golgi bodies or endosomes (Fig. 9b). Functionally, the R48D variant conferred markedly reduced As(III) tolerance compared with wild-type MpACR3 and the R48A mutant (Fig. 9c). These findings indicate that the R48D substitution impairs not only ER retention in the absence of As(III) but also overall MpACR3 function, potentially due to protein misfolding.

To determine whether substitutions at L45, R46, or R48 could overcome the intracellular retention of the C47A and F49A variants and restore their accumulation at the PM, we generated double variants. All tested double mutants, including R46A C47A and R46A F49A, predominantly localized to the PM, irrespective of As(III) exposure (Fig. 9b). Despite their PM localization, these double variants showed only partial restoration of As(III) tolerance and failed to reach wild-type levels (Fig. 9c). The impaired function of R46A C47A and C47A R48A was further confirmed by As(III) accumulation assays, in which yeast expressing these variants showed reduced arsenic transport activity at 1 mM As(III) (Fig. 9d).

Interestingly, yeast cells expressing the R46A or R48A single variants accumulated more As(III) than those expressing wild-type MpACR3 (Fig. 9d). Moreover, these variants failed to confer resistance at high As(III) concentrations (Fig. 9e). However, consistent with their ability to support yeast growth at low As(III) concentrations (Fig. 9c and e), expression of R46A, C47A, R48A, and their double variants prevented excessive arsenic accumulation in yeast exposed to 0.1 mM As(III) (Fig. 9f). Nevertheless, clear differences in the ability to reduce intracellular arsenic accumulation remained between the variants and wild-type MpACR3, suggesting that possible changes in the conformation of the N-terminal arsenic sensor may negatively affect MpACR3 function at the PM.

In summary, our results define a Φ-R-X_1_-R-X_2_ motif required for MpACR3 intracellular retention, where Φ represents a bulky hydrophobic residue. Efficient As(III)-induced relocalization additionally requires a cysteine residue at X_1_ and a bulky hydrophobic residue at X_2_, forming a Φ-R-C-R-Φ motif. Further studies of other plant ACR3-family members will be required to define this motif conclusively.

### The N-terminal di-arginine motif and arsenic-sensing cysteine residues control MpACR3 retention and relocalization in plant cells

To determine whether MpACR3 sorting and function follow the same mechanism in plant cells, we transiently expressed wild-type MpACR3-mVenus and its R46A, C47A, R46A C47A, AAA, and AAA R46A variants in tobacco leaf epidermal cells. Under control conditions, wild-type MpACR3 and the C47A and AAA variants localized to the Golgi and PM, whereas R46A, R46A C47A, and AAA R46A variants were found predominantly at the PM (Fig. 10a). Upon As(III) exposure, wild-type MpACR3, but not the C47A and AAA variants, relocalized from the Golgi to the PM (Fig. 10a).

**Figure 10:**
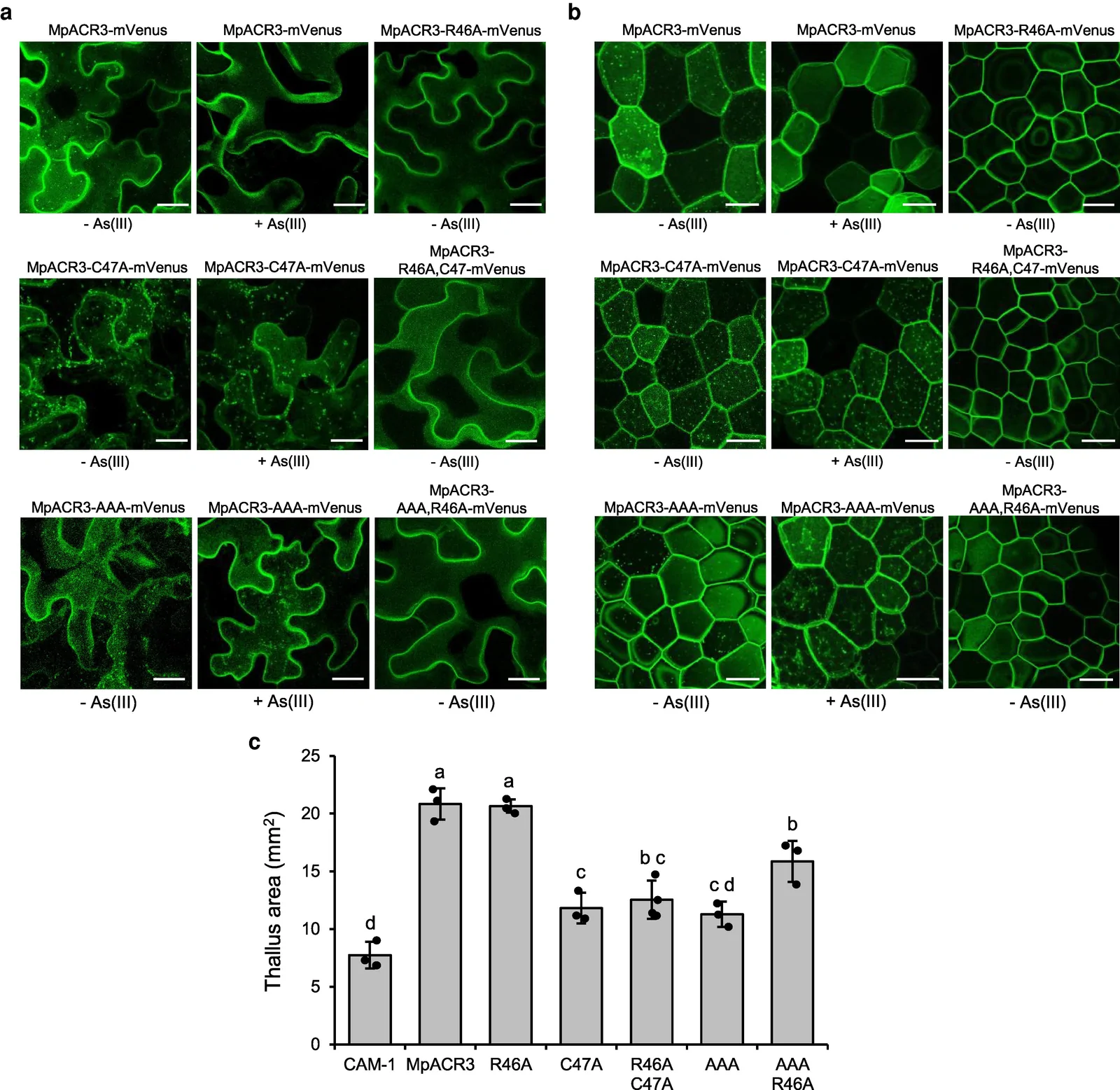
Roles of As(III)-sensing cysteines and the di-arginine motif in orchestrating MpACR3 trafficking to the plasma membrane in plant cells. a) Tobacco leaf epidermal cells transiently expressing wild-type MpACR3 and the indicated variants tagged with mVenus were visualized by confocal microscopy 3 d after infiltration and then exposed to 100 μM As(III) for 24 h or mock-treated. Images are maximum-intensity projections generated from Z-stacks. Scale bars = 30 μm. Images are representative of 3 independent transient-expression experiments. In each experiment, 2 leaves were infiltrated per construct and treatment, and 2 fragments from each leaf were analyzed. At least 10 images were acquired for each construct and treatment. b) Subcellular localization of MpACR3-mVenus and the indicated variants in Marchantia gemmae in the absence or presence of 25 μM As(III) for 24 h, visualized by confocal microscopy. Images are representative of 3 gemmae per genotype, and 2 fields of view were examined for each condition. Scale bars = 25 μm. c) Total thallus area of the indicated Marchantia lines grown for 10 d on 0.5× Gamborg's B5 medium supplemented with 50 μM As(III). The projected area of thallus tissue, excluding rhizoids, was measured using Fiji. Three independent lines per genotype, each with 9 thalli, were analyzed. The mean value of each independent line was used as the biological replicate. Error bars represent mean ± SD. Statistical significance was assessed using one-way ANOVA followed by Tukey's HSD test. Different letters indicate statistically significant differences (*P*-value < 0.05).

Next, we generated Marchantia transgenic lines overexpressing the same set of MpACR3-mVenus variants. Microscopic analysis of Marchantia gemmae showed that wild-type MpACR3 exhibited As(III)-driven relocalization from Golgi bodies to the PM, whereas all tested variants carrying the R46A substitution localized to the PM under all conditions tested (Fig. 10b). By contrast, the cysteine variants, C47A and AAA, failed to relocalize from the Golgi to the PM after As(III) treatment (Fig. 10b). Consequently, both cysteine variants failed to significantly increase As(III) resistance compared with wild-type MpACR3, whereas R46A remained functional (Fig. 10c; Figure S12). Interestingly, the R46A substitution did not restore the function of cysteine-to-alanine variants (Fig. 10c; Figure S12), despite their localization at the PM (Fig. 10b). We hypothesize that these mutations differentially affect the conformation of the N-terminal domain and, in some cases, may compromise MpACR3-dependent arsenic detoxification even when the protein reaches the PM. Whether the wild-type N-terminal domain additionally influences MpACR3 function at the PM in an As(III)-dependent manner requires further investigation.

Based on these results, we conclude that the di-arginine motif is functional in plant cells and plays a key role in MpACR3 intracellular retention in the absence of As(III), whereas the triad of cysteine residues is involved in As(III) sensing and promotes rapid accumulation of MpACR3 at the PM.

### Molecular modeling predicts As(III)-dependent remodeling of the MpACR3 N-terminal sensor

The biochemical data indicated that As(III) interacts with the MpACR3 N-terminal domain and increases its resistance to proteolysis, suggesting ligand-induced structural stabilization (Fig. 8). To explore how As(III) binding could affect the conformation of this domain, we performed molecular dynamics simulations of the MpACR3 N-terminal region encompassing the conserved cysteine-rich segment and the conserved 45-LRCRF-49 motif. We compared 3 models: the wild-type N-terminal domain (WT), the C29A/C47A/C71A variant (hereafter referred to as Triple Ala), and a restrained wild-type (WT with restraints) model in which C29, C47 and C71 were positioned in an As(III)-compatible geometry (Fig. 11; Figures S13–S19).

**Figure 11:**
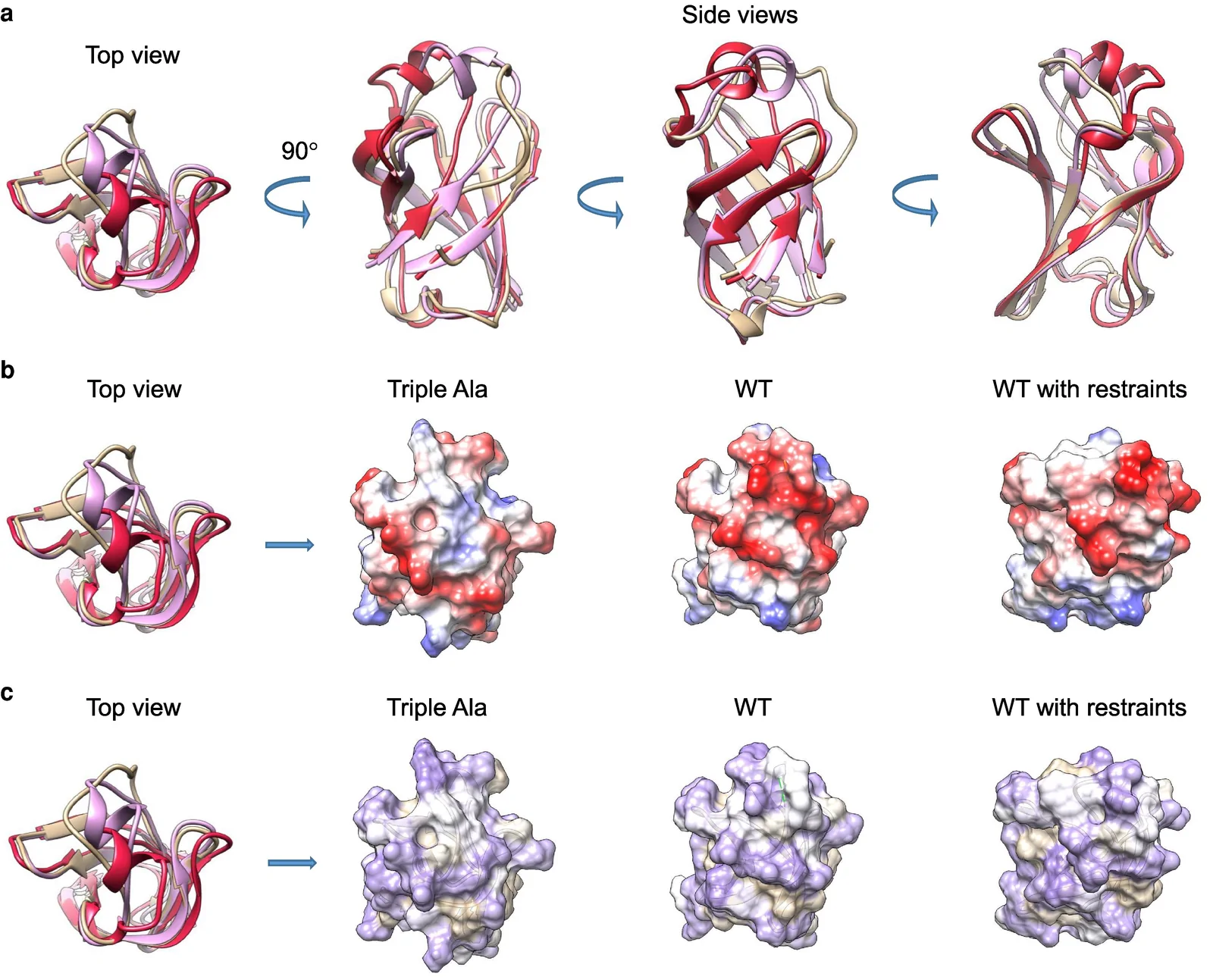
Structural modeling of the MpACR3 arsenic-sensing domain. a) Comparison of molecular dynamics trajectory-averaged structures of the MpACR3 N-terminal domain: the Triple Ala model C29A C47A C71A (tan), wild-type MpACR3 (WT; pink), and WT modeled with Cys-Cys distance restraints (dark red). The models are shown in 4 successive orientations, with arrows indicating the direction of rotation. The most prominent structural difference is the formation of a helical region between residues 32 and 45. b) Electrostatic surface potentials of the 3 models viewed from the top, highlighting the structural differences in the same region. Red indicates negative electrostatic potential, white indicates neutral potential, and blue indicates positive electrostatic potential. c) Molecular surface comparison based on amino acid hydrophobicity, shown in the same orientation for all 3 models. Purple indicates the most hydrophilic surface regions, and tan indicates the most hydrophobic surface regions. For (b) and (c), the same color scheme was applied to all 3 models.

The simulations predicted that the C29/C47/C71-containing region is structurally sensitive to cysteine positioning. In the WT model, residues 35–40 showed a tendency to adopt an α-helical conformation. This conformational behavior may be explained by 2 hydrogen bonds present in the WT structure: the C71 HG-F69 O bond, which appears to promote formation of the C29 HG-N65 OD1 bond (Figure S19a). When the distance restraints were applied, mimicking an As(III)-compatible arrangement of C29, C47, and C71, the α-helical region expanded further to residues 32–45. By contrast, the corresponding region remained more disordered in the Triple Ala model throughout the entire trajectory (Fig. 11a; Figures S13, S15, and S17–S19). Thus, positioning of C29, C47, and C71 in an As(III)-compatible arrangement is predicted to stabilize secondary structure immediately preceding the conserved LRCRF motif. Furthermore, comparison of the root mean square fluctuation (RMSF) plots for the WT and restrained-WT models revealed increased conformational flexibility in the region spanning residues 85–95 (Figure S15). This increased flexibility resulted from the loss of 2 hydrogen bonds, K28 H–E97 O and R46 H–L87 O, when C29, C47, and C71 shifted into a geometry compatible with coordination of an As(III) atom (Figure S18a). We hypothesize that this increased flexibility may facilitate protein–protein interactions.

The restrained-WT model also showed a more compact structure as well as changes in molecular surface properties near this region (Fig. 11). In particular, the formation of the α-helical segment was associated with altered electrostatic potential (Fig. 11b) and hydrophobicity around the LRCRF-containing surface (Fig. 11c). Because the LRCRF motif functions as an arginine-based intracellular retention signal, these changes suggest that As(III)-dependent remodeling of the N-terminal sensor could alter the accessibility or recognition of this motif by trafficking factors.

Importantly, this model also provides a structural rationale for the dual role of the LRCRF motif in retention and arsenic-dependent release. Within this motif, L45, R46 and R48 contribute to basal intracellular retention, whereas C47 and F49 are required for As(III)-induced release from retention. The simulations place C47 at the interface between the cysteine-based sensor and the LRCRF sorting motif, suggesting that C47 is not merely one residue within a linear retention sequence but a coupling residue that links As(III)-dependent cysteine rearrangement to remodeling of the retention motif. Similarly, the requirement for F49 may reflect a structural role in stabilizing or transmitting the As(III)-induced conformational change within the LRCRF region. Thus, the LRCRF motif appears to integrate 2 functions: arginine-dependent intracellular retention and cysteine/phenylalanine-dependent release in response to As(III).

Together, the simulations support a model in which As(III) binding to the conserved C29/C47/C71 cysteine triad remodels the structural environment of the LRCRF motif. This provides a mechanistic explanation for the experimental observation that C29, C47, C71, and F49 are required for As(III)-induced release of MpACR3 from intracellular retention and its accumulation at the PM, whereas mutation of retention residues such as R46 or R48 shifts MpACR3 toward constitutive PM localization. Because the simulations approximate the As(III)-coordinated state using cysteine-distance restraints rather than explicit As(III)-thiolate parameterization, they should be interpreted as a structural prediction that is consistent with the biochemical and trafficking data, rather than as direct proof of the bound-state structure.

## Discussion

Adaptation of plants to arsenic-contaminated environments has required the evolution of specialized mechanisms for arsenic transport, sequestration, and detoxification. These mechanisms enable plants to survive and grow despite exposure to arsenic in soil and water. In this study, we functionally characterized the arsenite transporter ACR3 from *M. polymorpha* (MpACR3) and identified a previously unrecognized regulatory strategy employed by this membrane protein. We show that MpACR3 uses its N-terminal cysteine-rich domain as an arsenic-sensing module that dynamically responds to arsenic exposure to regulate MpACR3 intracellular trafficking. Our findings reveal a mechanism by which plants fine-tune arsenic efflux at the molecular level to limit intracellular As(III) accumulation.

Two recent studies have provided preliminary insights into MpACR3 function, suggesting that it plays a key role in arsenic tolerance by mediating As(III) efflux from the cytoplasm to the extracellular space (Dutta et al. 2024; Li et al. 2024). We showed that Mp*ACR3* overexpression in budding yeast, Arabidopsis, and Marchantia confers tolerance to both As(III) and As(V) and reduces arsenic accumulation in yeast cells and plant tissues (Figs. 1b, 2b–e, 4, and 7; Figure S8). These findings are consistent with Dutta et al. (2024), who demonstrated that MpACR3 overexpression in the yeast *acr3*Δ strain enhanced As(III) tolerance while reducing intracellular arsenic accumulation. Furthermore, they support the results of Li et al. (2024), who reported that Mp*ACR3* knockout in Marchantia increased sensitivity to both As(III) and As(V), accompanied by higher arsenic accumulation in transgenic plants exposed to As(V).

The ability to detect toxic metal(loid)s or excessive levels of essential metals and rapidly activate or upregulate efflux transporters and downregulate uptake pathways is vital for the survival of all organisms. This is especially critical for land plants, which are immobile and must adapt to their environment. The regulation of membrane transporters includes transcriptional control, trafficking and subcellular localization, membrane potential and pH modulation, ligand-mediated regulation, and post-translational modifications (Zbieralski et al. 2024; Niño-González and Duque 2025).

In bacteria, expression of As(III) efflux transporter genes located in the *ars* operon is repressed by the ArsR repressor under nonstress conditions (Wu and Rosen 1993). Binding of As(III) to 3 vicinal cysteine residues in ArsR triggers a conformational change in the ArsR dimer and its release from the promoter, leading to activation of the *ars* operon (Shi et al. 1996). In *S. cerevisiae*, the Yap8 transcription factor, which activates expression of the *ScACR3* gene (Wysocki et al. 2004), also acts as the As(III) sensor (Kumar et al. 2015). Binding of As(III) to 3 cysteine residues in Yap8 leads to its stabilization and activation (Kumar et al. 2015). In contrast to bacteria and yeast, Mp*ACR3* is expressed under control conditions, and its expression is further increased in the presence of arsenic compounds (Li et al. 2024) (Fig. 5e; Figure S10). This upregulation may be driven by an arsenic-responsive transcription factor that directly senses arsenic or may result from activation of a broader general or oxidative stress response. Because this study focuses on the sorting and intracellular trafficking of MpACR3 as a membrane transporter, the regulatory basis of arsenic-dependent changes in MpACR3 abundance remains to be explored in future work.

Beyond transcriptional regulation, our data indicate that MpACR3 undergoes As(III)-dependent redistribution from intracellular secretory compartments to the PM in multiple systems. However, the steady-state intracellular compartment differs between yeast and plant cells: MpACR3 is retained predominantly in the ER in yeast (Fig. 1d and e), whereas in plant cells it localizes mainly to Golgi bodies and the PM, with only weak overlap with ER markers (Figs. 2f and g and 5). High-resolution microscopy further pinpointed its specific localization to trans-Golgi cisternae when transiently expressed in tobacco leaves (Fig. 3). Consistently, Li et al. (2024) reported that the MpACR3-Citrine fusion protein predominantly localizes to the PM, with additional presence in unidentified cytosolic structures. Upon exposure to arsenic or antimony species, MpACR3 accumulates at the PM, with reduced retention in the ER or/and Golgi (Figs. 1g, 2f and g and 5; Figures S7 and S9 and Video S1). Thus, MpACR3 trafficking is conserved as an arsenic-responsive process, although the precise intracellular retention compartment appears to be system- and cell-type-dependent.

Moreover, we demonstrate that the large plant-specific N-terminal domain governs intracellular retention of MpACR3 (Fig. 7a and b; Figures S1 and S3). Mutational and biochemical analyses of the MpACR3 N-terminal domain strongly support its role as an As(III) sensor (Figs. 7d–f, 8, 9, and 10a and b; Figure S11).

We propose that As(III) interaction involves 3 conserved cysteine residues, C29, C47, and C71, triggering a conformational remodeling that may favor MpACR3 trafficking to the PM. MpACR3 variants lacking any of these cysteines, or the conserved F49 residue that may help maintain the responsive conformation, failed to relocalize efficiently to the PM in response to As(III) treatment (Figs. 7d–f, 9b, and 10a and b). Although As(III) sensing via cysteine residues is a known mechanism, as observed in bacterial ArsR and yeast Yap8, where As(III) binding triggers conformational changes that regulate gene expression (Shi et al. 1996; Wysocki et al. 2004; Kumar et al. 2015), our findings reveal a novel role for this process in the direct control of membrane protein trafficking. MpACR3 uses its N-terminal domain as an As(III) sensor to modulate its own subcellular localization, representing a regulatory strategy not previously described in eukaryotic arsenic detoxification systems.

We also investigated how the N-terminal As(III) sensor regulates intracellular retention and identified a highly conserved 45-LRCRF-49 motif that includes C47 and F49, both of which are required for efficient As(III)-responsive relocalization of MpACR3 to the PM (Figs. 9–11). The combined mutational and modeling data suggest that the LRCRF motif is not a simple linear retention signal. Instead, it appears to act as an integrated regulatory module in which L45, R46, and R48 mediate intracellular retention, while C47 and F49 couple arsenic sensing to release from retention. This arrangement may allow a single short motif to function both as a sorting signal under nonstress conditions and as a ligand-responsive switch after As(III) binding.

Notably, the sequence of the LRCRF motif resembles a known arginine-based sorting signal (LRXR), which plays a crucial role in the retention of membrane proteins in the ER and Golgi (Michelsen et al. 2005; Banfield 2011; Lujan and Campelo 2021). This motif has been shown to interact with COPI to facilitate retrograde transport within the Golgi and from the Golgi to the ER (Zerangue et al. 2001; Michelsen et al. 2005; Beck et al. 2009). Consistent with this role, the single substitution MpACR3 variants L45A, R46A, and R48A, as well as the double variants L45A C47A, R46A C47A, and C47A R48A, were predominantly localized to the PM when expressed in yeast (Fig. 9). Likewise, the R46A, R46A C47A, and R46A AAA (C29A C47A C71A) MpACR3 variants localized predominantly to the PM in Marchantia and tobacco leaves, consistent with a role for the LRXR motif in MpACR3 intracellular retention in the absence of As(III) (Fig. 10). This interpretation is supported by the previously defined consensus for arginine-based retention/retrieval signals, Φ/Ψ/R-R-X-R, in which Φ/Ψ denotes an aromatic or bulky hydrophobic residue and X represents any amino acid (Zerangue et al. 2001). In agreement with this consensus, replacing leucine with another bulky hydrophobic residue, valine or phenylalanine, did not impair the function of this motif as a retention signal (Fig. 9). However, whether the MpACR3 N-terminal domain physically interacts with COPI in the absence or presence of As(III), and whether the LRXR motif is required for such an interaction, remains to be determined.

We speculate that As(III)-dependent remodeling of the MpACR3 N-terminal sensor reduces the effectiveness of the LRXR-based retention signal, thereby shifting the balance toward forward trafficking. This could occur through several nonmutually exclusive mechanisms, including altered binding to COPI or other trafficking/regulatory factors, post-translational modifications, or recruitment of accessory proteins. Consistent with a ligand-dependent structural switch required for efficient As(III)-responsive relocalization of MpACR3, As(III) binding increases protease resistance of the N-terminal domain (Fig. 8b and c), while restrained molecular dynamics simulations suggest compaction and changes in secondary structure and surface properties near the 45-LRCRF-49 region (Figs. 9 and 11). Importantly, these data do not define the trafficking mechanism or interaction partners, which remain to be identified experimentally.

Intriguingly, although the N-terminal As(III) sensor plays a similar role in MpACR3 intracellular retention in both yeast and plant cells, we observed a key difference in its localization: MpACR3 is retained in the ER in yeast (Fig. 1d and e) but localizes predominantly to the Golgi in Arabidopsis and Marchantia cells, with a smaller fraction associated with the ER (Figs. 2f–g and 5). This discrepancy may reflect differences between yeast and plants in membrane lipid and protein composition, Golgi apparatus organization, the number of COPI subclasses, and the subcellular localization of enzymes that modify newly synthesized membrane proteins (Donohoe et al. 2007; Suda and Nakano 2012; Gao et al. 2014).

Our findings raise 2 broader evolutionary questions: why did plants evolve such elaborate mechanisms for regulating arsenic detoxification, and why do fern ACR3 proteins appear to lack the arsenic-sensing domain found in many other plants and algal ACR3 homologs? The dual localization of MpACR3 in Golgi bodies and at the PM under nonstress conditions may provide an adaptive balance between preparedness and regulation. Basal PM localization could allow low-level As(III) efflux when cells encounter background arsenic, whereas the Golgi-localized pool may serve as a rapidly mobilizable reserve that can be redirected to the PM upon arsenic exposure. Such a mechanism would provide a faster response than transcriptional induction alone. Arsenic-induced expression of MpACR3 may therefore reinforce detoxification during sustained stress, whereas arsenic-triggered relocalization of pre-existing protein provides an early response to sudden As(III) exposure. This mechanism differs from that of fern ACR3 proteins, which appear to lack related N-terminal domains, suggesting that distinct plant lineages may have evolved alternative strategies for ACR3 regulation. In Marchantia and possibly in other green algae and nonvascular plant lineages with related N-terminal domains, arsenic-regulated trafficking may have been advantageous in fluctuating environments, allowing rapid activation of detoxification while avoiding constitutive accumulation of the transporter at the PM in the absence of stress. By contrast, fern ACR3 proteins may rely more strongly on high basal activity, achieved through constitutive localization at the PM and vacuolar membrane, increased ACR3 gene copy number, or other regulatory mechanisms (Indriolo et al. 2010; Chen et al. 2017, 2021; Sun et al. 2023). Such strategies could be advantageous in species adapted to persistent arsenic exposure or arsenic hyperaccumulation. Thus, the absence of the N-terminal sensing domain in fern ACR3 s does not necessarily indicate a less effective system, but rather a different evolutionary solution to arsenic tolerance.

In conclusion, our study reveals an arsenic-responsive trafficking mechanism that regulates the subcellular distribution of the Marchantia ACR3 transporter. By identifying a conserved N-terminal regulatory domain that links As(III) sensing to MpACR3 relocalization from intracellular compartments to the PM, we uncover a previously unrecognized layer of control in plant arsenic detoxification. The cysteine-rich region and the LRCRF retention motif defined here provide a molecular framework for future work on how ligand binding, conformational remodeling, and intracellular retention are coordinated to control transporter deployment under metalloid stress. Given the broad conservation of related N-terminal domains among plant ACR3 proteins, this mechanism may represent an ancient adaptation that allowed early land plants to respond rapidly to arsenic exposure. Beyond its evolutionary significance, this knowledge may inform future strategies to engineer arsenic-responsive transport systems, modulate arsenic accumulation in plants, and develop new tools for biotechnology, environmental monitoring, or phytoremediation.

## Materials and methods

### Yeast material, transformation, and growth conditions

The *S. cerevisiae* strains used in this study include RW104 (*acr3*Δ*::kanMX*) (Wysocki et al. 2001) in the W303-1a background (*MAT*a *leu2-3 112 trp1-1 can1-100 ura3-1 ade2-1 his3-11,15*), as well as the Δ-s-tether strain (*MAT*a *ice2*Δ*::natMX4 ist2*Δ*::hisMX6 scs2*Δ*::TRP1 scs22*Δ*::hisMX6 tcb1*Δ*::kanMX6 tcb2*Δ*::kanMX6 tcb3*Δ*::hisMX6*), provided by Christopher T. Beh (Simon Fraser University, British Columbia, Canada), in the SEY6210 background (*MAT*α *leu2-3,112 ura3-52 his3*Δ*200 trp1*Δ*901 lys2-801 suc2*Δ*9*) (Quon et al. 2018). The *acr3*Δ*::kanMX* strain expressing the ER marker *proTPI1-SS-mCherry-HDEL* at the *TRP1* locus was generated via transformation with *Pac*I-linearized pZJOM90 plasmid (Addgene plasmid #133647; http://n2t.net/addgene:133647) (Zhu et al. 2019).

Yeast cells were grown at 30 °C in selective synthetic dextrose (SD) medium supplemented with adenine at 100 μg/mL and appropriate amino acids required for plasmid selection. To assess metalloid sensitivity, yeast cell cultures were serially diluted and plated on selective SD medium containing varying concentrations of sodium arsenite [As(III)] or sodium arsenate [As(V)] (Sigma-Aldrich). Plates were incubated at 30 °C and photographed after 2 d. To examine the subcellular localization of MpACR3-GFP in budding yeast, cells were cultured in liquid SD medium supplemented with As(III), As(V), Sb(III), Sb(V), Cd(II), Cu(II), Zn(II), or H_2_O_2_ at the concentrations indicated in Fig. 1g for up to 2h before microscopy.

### Plant material and growth conditions

Male gametophytes of *M. polymorpha* Cam-1 and 3 independent transgenic lines generated in this study, MpACR3-1, MpACR3-2, and MpACR3-3 (see Gene cloning section) were cultured on solid 0.5× Gamborg B5 medium (Duchefa Biochemie, pH 5.8) in Petri dishes. Growth conditions included continuous light (150 μmol m^−2^ s^−1^) at 21 ± 1 °C in an MLR-352-PE climatic test chamber (PHCbi). For arsenic sensitivity and accumulation measurements, gemmalings derived from fresh gemmae were cultured on solid 0.5× Gamborg B5 medium with or without the indicated concentrations of As(III) or As(V) for 3 weeks.

All experiments in this study were conducted using the *A. thaliana* Columbia-0 (Col-0) ecotype. The following marker lines, previously characterized by Geldner et al. (2009), were obtained from the Salk Institute: Wave9R (VAMP711-mCherry, vacuole marker), Wave22R (SYP32-mCherry, Golgi marker), Wave127R (MEMB12-mCherry, Golgi marker), and Wave138R (PIP1;4-mCherry, PM marker). The ER marker line mCherry-HDEL (Nelson et al. 2007) was generously provided by Marcela Rojas-Pierce (North Carolina State University, US). The *_pro_35S:*Mp*ACR3-GFP* transgenic line was generated during this study (see Gene cloning section). For colocalization studies, the *_pro_35S:*Mp*ACR3-GFP* T27 line was crossed with the relevant mCherry marker lines described above.

Arabidopsis plants were cultivated under a 16 h light/8 h dark photoperiod at 22 °C, either in soil or in vitro on 0.5× Murashige and Skoog (MS) medium (Sigma-Aldrich) supplemented with 1% sucrose. To compare phenotypic differences between wild-type Arabidopsis and transgenic *_pro_35S:*Mp*ACR3-GFP* lines (T21 and T27), plants were grown in soil. Upon completing their growth cycle, measurements were taken for main stem height, number of flowers on the main stem, total number of branches, and total number of flowers. For the germination assay, sterilized seeds from wild-type and transgenic lines were sown on MS medium with or without 100 µM As(III) or 700 µM As(V) and allowed to grow for 14 d. For the growth assay, wild-type and transgenic seeds were initially sown on MS medium without arsenic. After 4 d, seedlings were transferred to MS medium supplemented with either 25 µM As(III), 1,000 µM As(V), or no arsenic (control) and grown for an additional 7 d.

### Quantitative RT-PCR

The gametophytes were grown on solid 0.5× Gamborg B5 medium for 2 weeks in the presence or absence of As(III) or As(V). Material for total RNA isolation was collected by cutting off the terminal fragments of Marchantia thalli with a sterile scalpel. Total RNA was extracted from 50 mg of plant tissue using the Beadbeat Total RNA Mini kit (A&A Biotechnology, Gdansk, Poland). First-strand cDNA from RNA samples was then synthesized using the High-Capacity cDNA Reverse Transcription Kit (Applied Biosystems TM). Quantitative PCR (qPCR) was performed using cDNA as a template, the 2× PCR Master Mix SYBR kit (A&A Biotechnology, Gdansk, Poland), and the CFX Connect Real-Time PCR Detection System (Bio-Rad, Hercules, CA, US) in a total volume of 15 μL. Primers specific for endogenous Mp*ACR3*, the *mVenus* region of the transgene, and the reference gene Mp*APT* (*adenine phosphoribosyltransferase*) used for qPCR are listed in Table S2. Mp*APT* was used for normalization. The amplification conditions were as follows: 1 min at 95 °C, 40 cycles of 10 s at 95 °C, 15 s at 60 °C, and 20 s at 72 °C. Finally, melting-curve analysis was conducted to verify the specificity of the reaction. The comparative threshold cycle (CT) method for relative quantification (ΔΔCT method) was used to analyze the data.

### Gene cloning

The oligonucleotides used for *ACR3* cloning and verification are listed in Table S2, while the plasmids utilized in this study are detailed in Table S3. To clone the Mp*ACR3* gene for heterologous expression in yeast, total RNA was isolated from *M. polymorpha* Cam-1 gametophytes. RNA extraction was performed using the BeadBeat Total RNA Mini Kit (A&A Technology). cDNA was synthesized using PrimeScript RT Reagent Kit (Takara), and the Mp*ACR3* coding sequence was amplified by PCR using CloneAmp HiFi PCR Premix (Takara). The amplified sequences were cloned into the pUG35 vector, fused in frame to a C-terminal GFP tag, and placed under the control of the *MET17* promoter using the In-Fusion HD Cloning Kit (Takara). The resulting pMpACR3 plasmid was verified by DNA sequencing. The pScACR3 plasmid, which overexpresses the *S. cerevisiae _pro_MET17:ScACR3-GFP* fusion gene, was described previously (Maciaszczyk-Dziubinska et al. 2014).

Plasmids encoding the N-terminal swap variants, N_Sc_-MpACR3-GFP and N_Mp_-ScACR3-GFP, were constructed through recombination. This was achieved by combining PCR-generated linear fragments of pMpACR3 or pScACR3, each lacking the respective N-terminal region of ACR3, with fragments encoding the N-terminal tail of ScAcr3 or MpACR3, respectively, using the In-Fusion HD Cloning Kit. Similarly, the plasmid encoding the MpACR3-Δ27-98-GFP variant was generated by fusing a PCR-amplified pMpACR3 linear plasmid excluding the sequence encoding the N-terminal cysteine-rich region (residues 27-98), followed by in-fusion-mediated circularization.

For heterologous expression in yeast, sequences encoding residues 1-123 of the wild-type Mp*ACR3* N-terminal domain (MpACR3-N-tail) and its cysteine-lacking variants (MpACR3-AAA-N-tail and MpACR3-Cys-null-N-tail) were cloned into the *Sal*I-digested pGREG535 vector (EUROSCARF, P30360). These constructs enabled expression under the *GAL1* promoter with an N-terminal 7×HA tag. For bacterial expression and subsequent protein purification, DNA sequences encoding the same wild-type and mutant Mp*ACR3* N-terminal fragments were synthesized and cloned into the pGEX4T-1 plasmid by BioCat GmbH. The resulting plasmids, pGST-MpACR3-N-tail, pGST-MpACR3-AAA-N-tail, and pGST-MpACR3-Cys-null-N-tail, allowed for the inducible expression of N-terminally GST-fused proteins in *E. coli* BL21(DE3) cells.

For plant expression, the synthetic Mp*ACR3* gene sequence was fused in frame to a C-terminal mVenus tag and placed under the control of either the 2 × 35S promoter (*_pro_2* × *35S:*Mp*ACR3-mVenus* and its mutant variants) or the native promoter (*_pro_*Mp*ACR3:*Mp*ACR3-mVenus*). This was achieved using Type IIS Loop assembly and components from the OpenPlant toolkit (Pollak et al. 2019; Sauret-Güeto et al. 2020). The same strategy was used to generate *_pro_2* × *35S:*Mp*ACR3-mVenus* with either *_pro_*Mp*UbE2:52aaST-mScarlet* or *_pro_*Mp*UbE2:ER-mTurq2-HDEL* constructs for the Marchantia colocalization experiment. Additionally, the Mp*ACR3* coding sequence was amplified from the pMpACR3 plasmid using high-fidelity PCR and cloned into the *Xba*I-digested pGFPGUSPlus vector (Vickers et al. 2007; a gift from Claudia Vickers; Addgene plasmid #64401), resulting in the *_pro_35S:*Mp*ACR3-EGFP* construct, referred to throughout as *_pro_35S:*Mp*ACR3-GFP*.

### Mutagenesis

Plasmids carrying the Mp*ACR3* gene or its mutant variants served as templates for site-directed mutagenesis, performed using the QuikChange Lightning Kit (Agilent Technologies) to generate single and multiple substitution variants. The introduced mutations were verified by DNA sequencing. Oligonucleotides used for mutagenesis are listed in Table S2.

### Transformation

Yeast transformation was carried out using the lithium acetate/single-stranded carrier DNA/polyethylene glycol method (Gietz and Woods 2002).

For Marchantia transformation, *Agrobacterium tumefaciens* cells (GV3103) were electroporated with the following constructs: *_pro_2* × *35S:*Mp*ACR3-mVenus* or its mutant variants, *_pro_*Mp*ACR3:*Mp*ACR3-mVenus*, *_pro_2* × *35S:*Mp*ACR3-mVenus _pro_*Mp*UbE2:52aaST-mScarlet*, or *_pro_2* × *35S:*Mp*ACR3-mVenus _pro_*Mp*UbE2:ER-mTurq2-HDEL* using a Bio-Rad Gene Pulser. The transformed bacteria were plated on LB medium containing spectinomycin, rifampicin, and gentamycin, then incubated at 29 °C for 3 d. Marchantia spores (Cam-1×Cam-2) were sterilized and plated on 0.5× Gamborg B5 medium for 5 d, following the protocol described by Sauret-Güeto et al. (2020). The 5-d-old sporelings were then collected using a scalpel blade and resuspended in 4 mL of liquid 0.5× Gamborg B5 medium in sterile 6-well plates. The medium was supplemented with 0.1% N-Z amino A (Sigma-Aldrich, C7290), 0.03% (w/v) L-glutamine (Alpha Aesar, A14201), 2% (w/v) sucrose (Fisher Scientific, 10634932), and 100 μM acetosyringone. A plate containing transformed *A*. *tumefaciens* colonies was streaked with a sterile pipette tip, and ∼0.3 mm diameter sphere of bacterial cells was resuspended in the liquid medium containing Marchantia sporelings. The 6-well plate was shaken at 120 rpm for 2 d with continuous lighting (150 μmol m^−2^ s^−1^). Following cocultivation, the sporelings were transferred onto a 70 μm-pore cell strainer and washed with 10 mL of sterile water. They were then placed on solid 0.5× Gamborg B5 medium supplemented with 100 μg/mL cefotaxime (Apollo Scientific, BIC0111) and the appropriate selection agent: 20 μg/mL Hygromycin B (Invitrogen, 10687010) for *_pro_2* × *35S:*Mp*ACR3-mVenus* constructs or 0.5 μM chlorosulfuron (Thermo Scientific #17959385). For generation of the ER marker lines, sporelings were cotransformed with a 1:1 mixture of 2 *A. tumefaciens* GV3103 cultures carrying *_pro_*2 × 35S:Mp*ACR3(variants)-mVenus* and chlorosulfuron resistance, and *_pro_*Mp*UbE2:ER-mTurq2-HDEL* next to hygromycin resistance. Individual sporelings resistant to appropriate selection agents were counted as independent lines. Transformants emerged within a week and were subsequently transferred to fresh plates for gemma cup formation.

The *_pro_35S:*Mp*ACR3-GFP* construct was electroporated into *A. tumefaciens* cells GV3101 (pMP90) (Koncz and Schell 1986) and used to transform *A*. *thaliana* (Col-0) plants via the floral dip method (Clough and Bent 1998). Seeds from transformed *A*. *thaliana* plants were surface-sterilized and sown on MS medium supplemented with 25 μg/mL Hygromycin B (Sigma-Aldrich). After exposure to light for 6 h, the seeds were kept in the dark for 4 d before being transferred to a 16-h light/8-h dark photoperiod for further growth. Transformants, identified by their elongated hypocotyls and green true leaves, were then transferred to soil. Independent transformants were screened for GFP fluorescence, and 2 homozygous lines, T21 and T27, from the T3-T4 generations were used for the experiments presented in this study.

### Transient expression

Transient expression of MpACR3-GFP in *Nicotiana benthamiana* leaf epidermal cells was performed by *Agrobacterium*-mediated infiltration as described by Hawes et al. (2024). The *_pro_35S:*Mp*ACR3-GFP* was coexpressed with ST-mRFP, MNS1-mRFP, or mRFP-SYP61, and epidermal cells were imaged 3 d after infiltration. For analysis of MpACR3-mVenus variants in *N. benthamiana* leaf epidermal cells, *Agrobacterium*-mediated transient expression was performed using constructs encoding wild-type *_pro_2* × *35S:*Mp*ACR3-mVenus* or the indicated *_pro_2* × *35S:*Mp*ACR3-mVenus*-tagged variants. Leaves were imaged 3 d after infiltration under control conditions or after exposure to 100 μM As(III) for 24 h.

### FM4-64 staining

To label the vacuolar membrane in live yeast cells, 0.5 mL of an overnight yeast culture was washed, resuspended in SD medium containing 40 μM FM4-64 (Molecular Probes), and incubated in the dark for 2 h in a 30 °C water bath. The cells were then analyzed using an epifluorescence microscope.

For PM staining in *_pro_35S:*Mp*ACR3-GFP* Arabidopsis root cells, seedlings were transferred to 8 μM FM4-64 solution (dissolved in 0.5× MS liquid medium), incubated on ice for 2 min, and then washed twice with cold 0.5× MS liquid medium to remove excess FM4-64, followed by immediate confocal imaging to minimize dye internalization through the endocytic pathway (Rigal et al. 2015).

### Microscopy

The subcellular localization of MpACR3-GFP fusion proteins in live yeast cells was analyzed using an upright wide-field epifluorescence microscope (Axio Imager M2, Carl Zeiss, Germany) equipped with a Zeiss HBO 100 illuminator and a 100× oil immersion objective (Zeiss Plan-Neofluar 100×/1.30). Fluorescence signals were detected using either filter set 38 HE (GFP) or filter set 43 HE (mCherry, FM4-64). Images were captured with a Zeiss AxioCam MRc digital camera and processed using Zeiss ZEN 3.7 software. Representative images are shown from 2 independent yeast transformants per genotype, with 10–20 randomly selected cells analyzed for each transformant.

A Leica SP8 confocal microscope was used to image Marchantia sporelings and gemmae. A 460–670 nm WLL (White Light Laser) was used to excite mVenus (515 nm), mScarlet (560 nm), and mTurquoise2 (442 nm). Fluorescence signals were detected using the appropriate HyD detectors. Laser power and detector gain ranged from 5%–15% to 50%–150%, respectively, depending on the fluorophore and experimental series. Imaging was conducted with a Plan-Apochromat 40× water immersion objective.

An Olympus FluoView FV1000 confocal laser scanning microscope was used to image *_pro_35S:*Mp*ACR3-GFP* (excitation: 488 nm) in root cells expressing Wave-mCherry, HDEL-mCherry, or FM4-64 markers (excitation: 559 nm). Imaging was performed using a Plan-Apochromat 60× objective. Images were acquired from the root elongation zone, between the meristematic and root hair zones. Col-0 and previously validated MpACR3-GFP-expressing samples were used as negative and positive controls, respectively.

A Zeiss Lattice Lightsheet 7 (LLS7) fluorescence microscope was employed for visualizing MpACR3-GFP (488 nm laser) in Arabidopsis root cells, also coexpressing the Golgi marker Wave127R-mCherry (565 nm laser). Imaging parameters included: Sinc3 100 × 1,400, z-step 0.3 µm, 488 nm laser power 4%, exposure time 30 ms for MpACR3-GFP (T21 and T27 lines) or Sinc3 30 × 1,000, z-step 0.3 µm, 488 nm laser power 2%, 565 nm laser power 6%, exposure time 50 ms for MpACR3-GFP/Wave127R-mCherry. In total, more than 10 roots per treatment were analyzed for the T27 line, whereas 3 roots per treatment were analyzed for the T21 line. Images were processed using ZEN ZEISS 3.10 (Carl Zeiss Microscopy GmbH), applying medium-strength deconvolution, linear interpolation, deskewing, cover glass transformation, and image subset selection. Final images were displayed in 3D or as 2D single optical section.

A Leica STELLARIS 8 confocal microscope was used to capture MpACR3-mVenus signals in tobacco leaf epidermal cells (Fig. 10a). A 460–670 nm WLL (White Light Laser) was used to excite mVenus, and fluorescence signals were detected using HyD detectors. Laser power and gain were adjusted per sample, ranging from 5%–20% to 50%–100%, respectively. Imaging was performed with a Plan-Apochromat 40× water immersion objective. Three independent transient-expression experiments were performed. In each experiment, 2 leaves were infiltrated per construct and treatment, and 2 fragments from each leaf were analyzed. At least 10 images were acquired for each construct under control conditions and after As(III) treatment.

Colocalization of MpACR3-GFP with the marker proteins of Golgi compartments in tobacco leaf epidermal cells was visualized using Airyscan high-resolution confocal microscopy 3 d post infiltration. The analysis was based on the approach described by McGinness et al. (2022, 2025).

### Colocalization analysis

Colocalization was quantified for individual cells using Pearson's correlation coefficient (PCC) calculated with the colocalization tool in Olympus FV1000 Viewer. A PCC value of 1 indicates perfect correlation, whereas a value of 0 indicates no correlation. PCC values were calculated from 3 independent biological replicates, with a total of 15 cells analyzed.

### Arsenic accumulation measurements

Yeast transformants were grown in selective SD medium to logarithmic phase and then incubated with As(III) at the concentrations and for the durations specified in the corresponding figure legends. Following optical density measurement at 600 nm (OD_600_), cells were washed 3 times with cold deionized water and lysed in 2 mL of 0.5% nitric acid (EMPLURA, Merck) by boiling for 10 min. After centrifugation, the supernatants were analyzed for arsenic concentrations using an atomic absorption spectrometer with a graphite cuvette (contrAA 800 G, Analytik Jena). Arsenic accumulation was normalized to OD_600_.

For plant samples, a similar procedure was followed. Arabidopsis and Marchantia were grown on solid 0.5× MS or 0.5× Gamborg B5 medium, respectively, supplemented with different concentrations of As(III) or As(V) for 14 or 21 d. Pooled tissue fragments were weighed and snap-frozen in liquid nitrogen before homogenization in 1.5 mL of 0.5% nitric acid using glass beads and a Precellys 24 homogenizer (Bertin Technologies). The homogenized samples were boiled, centrifuged, and the arsenic concentrations in the supernatants were measured as described above. Arsenic accumulation was normalized to tissue fresh weight.

### Total protein extracts

Total protein extraction from yeast cells was performed using the trichloroacetic acid method (Wright et al. 1989). For plants, total protein extracts were obtained following the protocol of Martínez-García et al. (1999). Proteins were resolved on 10% SDS-PAGE, transferred to nitrocellulose membranes (Bio-Rad, 1620115), and probed with an anti-GFP antibody (Roche, 11814460001) at a 1:3,000 dilution to detect MpACR3-GFP and MpACR3-mVenus fusion proteins in yeast and plant samples. Ponceau S (Sigma-Aldrich, P-3504) staining was used to assess protein loading and transfer for plant samples. An anti-3-phosphoglycerate kinase (Pgk1) antibody (Abcam, 22C5D8) at a 1:15,000 dilution was used as a loading control for yeast proteins.

### MpACR3 N-terminal domain expression in yeast and trypsin susceptibility assay

Yeast *acr3*Δ cells transformed with pGREG535 plasmids encoding MpACR3 N-terminal domain variants were grown overnight in selective SD medium supplemented with 2% raffinose. The following day, ∼10^8^ cells from each transformant were transferred to selective SD medium containing 4% galactose and incubated for 3 h at 30 °C to induce expression. Cells were then either left untreated or exposed to 0.5 mM As(III) for 1 h before being centrifuged, washed with phosphate-buffered saline (PBS), and resuspended in 600 μL of lysis buffer containing PBS, a protease inhibitor cocktail (Sigma-Aldrich, P8215), and 1 mM PMSF (BioShop). Lysis was performed using 0.5 mm glass beads (Linegal Chemicals) with 3 30-s bead-beating cycles (Bertin Technologies) at 4,000 rpm. The lysates were then centrifuged at 850 × *g* for 5 min at 4 °C, and the supernatants were transferred to a fresh tube.

For the trypsin susceptibility assay, 100 μg of total protein from each yeast lysate was incubated on ice for 10 min with trypsin (Sigma-Aldrich) at concentrations of 0, 1, or 5 μg/mL. Proteolysis was halted by adding 0.5 mg/mL soybean trypsin inhibitor (Sigma-Aldrich) for 15 min. Protein degradation patterns were assessed by immunoblotting using an anti-HA antibody (Sigma-Aldrich, H6908) at a 1:5,000 dilution.

### As-biotin pulldown assay

Lysates of *acr3*Δ yeast cells expressing 7HA-MpACR3-N-tail, 7HA-MpACR3-AAA-N-tail, or 7HA-MpACR3-Cys-null-N-tail from the pGREG535 plasmid were prepared as described above. For the As-biotin pulldown assay, 200 μg of yeast lysate was incubated with 20 µM As-biotin (N-Biotinyl *p*-Aminophenyl Arsenic Acid, Santa Cruz, SC-503570) in 10 mM MES-Tris buffer (pH 6.0) at 25 °C for 1 h. In parallel, lysates were pretreated with 20 µM As(III) in 10 mM MES-Tris buffer (pH 6.0) for 30 min as a competition control before the addition of the As-biotin conjugate. Following incubation, lysates were diluted 10-fold with 10 mM MES-Tris buffer (pH 6.0) and mixed with 30 µL of streptavidin-agarose resin (Thermo Scientific), which had been pre-equilibrated with 0.05% Tween 20 buffer (1 wash) and 10 mM MES-Tris buffer (pH 6.0) (2 washes). Samples were incubated at 21 °C for 1 h with continuous overhead rotation. The resin was then briefly spun down and sequentially washed with NaCl buffer (1 M NaCl, 10 mM MES-Tris buffer, pH 6.0) for 2 min, followed by urea buffer (2 M urea, 10 mM MES-Tris buffer, pH 6.0) for 2 min. Bound proteins were eluted by incubation in 45 μL of Laemmli buffer (250 mM Tris-HCl, pH 6.8, 500 mM DTT, 10% SDS, 0.01% bromophenol blue, 50% glycerol) at 95 °C for 5 min. All fractions, including the input lysate (not incubated with As-biotin) and the eluate, were denatured at 95 °C for 10 min before Western blot analysis. A total of 40 µL of each fraction was analyzed by 12% SDS-PAGE, transferred to a nitrocellulose membrane (Bio-Rad), and immunoblotted using an anti-HA antibody (Sigma-Aldrich, H6908) at a 1:5,000 dilution.

### Purification of MpACR3 N-terminal domain and thermolysin digestion

*Escherichia coli* BL21(DE3) cells carrying the pGST-MpACR3-N-tail, pGST-MpACR3-AAA-N-tail, or pGST-MpACR3-Cys-null-N-tail plasmids were cultured at 37 °C to an OD_600_ of 0.8. Protein expression was then induced by adding 0.5 mM IPTG, followed by incubation at 30 °C for 4 h. Cells were harvested by centrifugation, and the resulting pellet was lysed by sonication (Bandelin Sonopuls) in lysis buffer (PBS, 10 mM 2-mercaptoethanol, 1% Triton X-100, 10% glycerol, protease inhibitor cocktail (Sigma-Aldrich, P8215), 1 mM PMSF). Sonication was performed in 10 cycles of 30 s each. The lysate was clarified by centrifugation at 21,000 × *g* for 30 min at 4 °C. Protein extracts were incubated with Glutathione Sepharose™ 4B resin (GE Healthcare) pre-equilibrated with PBS in a gravity-flow column. After 3 washes with PBS, bound proteins were eluted using an elution buffer containing 10 mM reduced glutathione (L-glutathione, Sigma-Aldrich) and 50 mM Tris-HCl (pH 8.0). The eluted proteins were concentrated using Amicon® Ultra-15 Centrifugal Filter Units (Merck Millipore) and stored at −80 °C until further use.

A stock solution of thermolysin from *Geobacillus stearothermophilus* (Sigma-Aldrich, T7902) was prepared at 2.5 mg/mL in proteolytic buffer (0.1 mM TCEP, 25 mM bis-Tris, pH 6.0). Before the proteolysis assay, the enzyme was further diluted 50-fold in proteolysis buffer. 5 µg of purified proteins were preincubated with or without 20 μM As(III) on ice for 1 h. After the preincubation, the protein solution was mixed with 0.5 μL of the diluted thermolysin solution in a final volume of 5 μL on ice. Reactions were incubated at 21 °C and terminated by the addition of an equal volume of hot 4× SDS sample buffer supplemented with 2 mM PMSF and 100 mM DTT, followed by heat denaturation at 95 °C for 5 min. Samples were immediately loaded onto SDS-PAGE gels, and proteolysis patterns were visualized by Coomassie staining. For MpACR3 variants, the thermolysin stock solution was diluted 100-fold before addition to the reaction mixture.

### Molecular modeling and molecular dynamics simulations

The AlphaFold model of MpACR3 (UniProt/AlphaFold entry A0A176WR38) (Jumper et al. 2021) was used as the starting structure for molecular modeling. The region corresponding to amino acid residues 24–105, which contains the conserved cysteine-rich N-terminal segment and the LRCRF retention motif, was extracted for further analysis. The structure was initially prepared using Maestro BioLuminate, Schrödinger suite v. 2021–3 (Salam et al. 2014). This preparation introduced 2 disulfide bonds, C47-C71 and C29-C96. Because the MpACR3 N-terminal domain is predicted to face the cytosol, these disulfide bonds were considered unlikely under physiological conditions. The structure was therefore manually edited in UCSF Chimera (Pettersen et al. 2004) to remove the disulfide bonds and add hydrogen atoms to the corresponding cysteine SG atoms. A Triple Ala (C29A C47A C71A) model was generated using the rotamer functionality in UCSF Chimera.

All-atom molecular dynamics simulations were performed using GROMACS v. 2021.4 (Abraham et al. 2015). The Amber14SB force field (Maier et al. 2015) was used for the protein, and the OPC water model was used for solvent (Izadi et al. 2014). Each system was placed in a triclinic periodic box with a minimum distance of 15 Å between the protein and the box edge. Systems were neutralized and supplemented with Na^+^ and Cl^−^ ions to a final concentration of 150 mM NaCl, using Joung–Cheatham ion parameters (Joung and Cheatham 2008). Periodic boundary conditions were applied. Each system was energy-minimized using 5,000 steps of steepest descent minimization, followed by 500-ps equilibration with weak positional restraints on solute heavy atoms in the NVT and NPT ensembles. Temperature and pressure were maintained at 300 K and 1 atm, respectively (Parrinello and Rahman 1981; Berendsen et al. 1984). After removal of positional restraints, 2-μs production simulations were performed for the WT and Triple Ala models.

To approximate the structural consequences of As(III)-dependent coordination by C29, C47, and C71, an additional restrained wild-type simulation was performed. Explicit parameterization of As(III)-thiolate coordination was not used, because this is nontrivial in standard biomolecular force fields. Instead, the As(III)-compatible state was modeled by restraining the distances between the side-chain sulfur (SG) atoms of C29-C47, C47-C71, and C29-C71. Distance restraints were applied using PLUMED (Bonomi et al. 2009) through a quadratic wall potential. The lower and upper wall limits were set to 3.5 and 3.8 Å, respectively, corresponding to SG-SG distances compatible with tricoordinate As(III)-cysteine arrangements observed in arsenic-binding proteins. The restraint force constant was initially set to 500 kJ mol^−1^ nm^−2^ and increased by 500 kJ mol^−1^ nm^−2^ every 200 ns to a final value of 2,500 kJ mol^−1^ nm^−2^. The restrained system was then simulated for an additional 2 μs to allow structural equilibration under the imposed cysteine-distance restraints.

Trajectory analyses were performed using GROMACS v. 2021.4 tools (Abraham et al. 2015). Structural stability and flexibility were assessed using root mean square deviation of protein heavy atoms (Figure S14) and per-residue RMSF (Figure S15). Secondary-structure changes were analyzed using the DSSP algorithm. The evolution of cysteine SG-SG distances was monitored throughout the trajectories (Figure S16). Average structures were generated from equilibrated trajectory segments and used for comparison of overall fold (Fig. 11a; Figure S13), secondary-structure organization (Figure S17) and surface properties (Fig. 11b and c). Electrostatic surface potential and hydrophobicity were visualized for the average structures using UCSF Chimera to identify conformational changes around the cysteine-rich region and the LRCRF retention motif. For visualizing the secondary-structure organization, we used the PDBsum web server (Laskowski et al. 2018). Molecular graphics were generated using UCSF Chimera, while data plotting was performed in MATLAB.

### Accession numbers

All accession numbers for genes and proteins mentioned in this study are provided in the Figure S1 legend and Table S1.

## Supplementary Material

koag306_Supplementary_Data

## Data Availability

All data and materials that support the findings of this study are included in the manuscript or in the supplementary materials.
