# Supplementary material for: Arsenic-sensing domain controls ACR3 transporter trafficking in Marchantia polymorpha
Source: Plant Cell. 2026 Oct 6;38(10):koag306. doi: 10.1093/plcell/koag306 (PMC13674352; doi:10.1093/plcell/koag306)
Supplement: Supplementary file 1 [file koag306_supplementary_data.zip › Supplementary tables_Bonter et al.docx]

**Arsenic-sensing domain controls ACR3 transporter trafficking in *Marchantia polymorpha***

Ignacy Bonter^1,2,ǂ^, Katarzyna Mizio^1,ǂ^, Alicja Dolzblasz^3^, Paulina Tomaszewska^1,4^, Kacper Zbieralski^1^, Jacek Staszewski^1^, Donata Wawrzycka^1^, Anna Reymer^5,6^, Wojciech Bialek^7^, Verena Kriechbaumer^8^, Jim Haseloff^2^, Robert Wysocki^1^, Ewa Maciaszczyk-Dziubinska^1,*^

^1^Department of Genetics and Cell Physiology, Faculty of Biological Sciences, University of Wroclaw, 50-328 Wroclaw, Poland

^2^Department of Plant Sciences, University of Cambridge, Downing Street, Cambridge, CB2 3EA, United Kingdom

^3^Department of Plant Developmental Biology, Faculty of Biological Sciences, University of Wroclaw, 50-328 Wroclaw, Poland

^4^Department of Genetics, Genomics and Cancer Sciences, University of Leicester, LE1 7RH, Leicester, United Kingdom

^5^Department of Chemistry and Molecular Biology, University of Gothenburg, 405 30 Göteborg, Sweden

^6^Institute for Advanced Biosciences (Inserm U1209), Site Santé – Domaine de la Merci, 38700 La Tronche, France

^7^Department of Biophysics, Faculty of Biotechnology, University of Wroclaw, 50-383 Wroclaw, Poland

^8^School of Biological and Medical Sciences and Centre for Bioimaging, Oxford Brookes University, Oxford OX3 0BP, United Kingdom

*Corresponding author: Ewa Maciaszczyk-Dziubinska, Department of Genetics and Cell Physiology, Faculty of Biological Sciences, University of Wroclaw, 50-328 Wroclaw, Poland. Email: ewa.maciaszczyk-dziubinska@uwr.edu.pl

^ǂ^These authors contributed equally to this article.

**Supplementary Tables S1 to S3**

**Table S1. Green algal and land plant ACR3 proteins analyzed or discussed in this study.**

| Protein name | Organism | Taxonomic group | Database | Protein identifier |
| --- | --- | --- | --- | --- |
| PsACR3 | *Picea sitchensis* | Gymnosperms | NCBI^1^ | ADE76924.1 |
| TpACR3 | *Thuja plicata* | Gymnosperms | Phytozome^2^ | Thupl.29379155s0008 |
| GbACR3 | *Ginkgo biloba* | Gymnosperms | Gigascience^3^ | Gb_32402 |
| WmACR3 | *Welwitschia mirabilis* | Gymnosperms | Data Dryad^4^ | W.mirabilis.22940 |
| GmACR3 | *Gnetum montanum* | Gymnosperms | Data Dryad^5^ | TnS000269885t05 |
| PvACR3 | *Pteris vittata* | Ferns | NCBI | ADP20955.1 |
| PvACR3;1 | *Pteris vittata* | Ferns | NCBI | ASV64940.1 |
| PvACR3;2 | *Pteris vittata* | Ferns | NCBI | QTG40769.1 |
| PvACR3;3 | *Pteris vittata* | Ferns | NCBI | QTG40770.1 |
| PvACR3;4 | *Pteris vittata* | Ferns | Sun et al. (2023)^6^ | transcript_28534 |
| CrACR3 | *Ceratopteris richardii* | Ferns | NCBI | KAH7279060.1 |
| AnACR3 | *Adiantum nelumboides* | Ferns | NCBI | MCO5594610.1 |
| SaACR3 | *Salvinia cucullata* | Ferns | Fernbase^7^ | Sacu_v1.1_s0024.g008994 |
| AsACR3 | *Alsophila spinulosa* | Ferns | Fernbase | Aspi01Gene69197.t1 |
| MvACR3;1 | *Marsilea vestita* | Ferns | Fernbase | Mvestita_S10g02657-RA |
| MvACR3;2 | *Marsilea vestita* | Ferns | Fernbase | Mvestita_S10g02658-RA |
| DcACR3;1 | *Diphasiastrum complanatum* | Lycophytes | NCBI | KAJ7529772.1 |
| DcACR3;2 | *Diphasiastrum complanatum* | Lycophytes | NCBI | KAJ7529768.1 |
| ItACR3;1 | *Isoetes taiwanensis* | Lycophytes | Fernbase | Itaiw_v1_scaffold_14_t11603-RA |
| ItACR3;2 | *Isoetes taiwanensis* | Lycophytes | Fernbase | Itaiw_v1_scaffold_2_t02514-RA |
| SmACR3 | *Selaginella moellendorffii* | Lycophytes | NCBI | XP_024531516.1 |
| PpACR3 | *Physcomitrium patens* | Mosses | NCBI | XP_024362327.1 |
| CpACR3 | *Ceratodon purpureus* | Mosses | NCBI | KAG0559408.1 |
| SfACR3 | *Sphagnum fallax* | Mosses | NCBI | KAH8961229.1 |
| MpACR3 | *Marchantia polymorpha* | Liverworts | NCBI | OAE35577.1 |
| RfACR3 | *Riccia fluitans* | Liverworts | NCBI | KAL2645082.1 |
| AaACR3 | *Anthoceros agrestis* | Hornworts | Hornwort genomes^8^ | AagrOXF_evm.model.utg000091l.433__434.1 |
| ApACR3 | *Anthoceros punctatus* | Hornworts | Hornwort genomes | Apun_evm.model.utg000254l.51.1 |
| MeACR3 | *Mesotaenium endlicherianum*  SAG 12.97 | Charophytes | Phycocosm^9^ | https://phycocosm.jgi.doe.gov/cgi-bin/dispGeneModel?db=Mesen1_1&id=7825 |
| ClACR3 | *Closterium sp.*  NIES-68 | Charophytes | NCBI | GJP46584.1 |
| KnACR3;1 | *Klebsormidium nitens* NIES-2285 | Charophytes | NCBI | GAQ91548.1 |
| KnACR3;2 | *Klebsormidium nitens* NIES-2285 | Charophytes | NCBI | GAQ89538.1 |
| CsACR3 | *Coccomyxa subellipsoidea*  C-169 | Chlorophytes | Phycocosm | https://phycocosm.jgi.doe.gov/cgi-bin/dispGeneModel?db=Cosub3&id=1208183 |
| CnACR3;1 | *Coccomyxa elongate* SAG216_3B | Chlorophytes | NCBI | CAL8460937.1 |
| CnACR3;2 | *Coccomyxa elongate* SAG216_3B | Chlorophytes | NCBI | CAL8465694.1 |
| CnACR3;3 | *Coccomyxa elongate* SAG216_3B | Chlorophytes | NCBI | CAL8467475.1 |
| CvACR3;1 | *Coccomyxa viridis* | Chlorophytes | NCBI | CAK0787439.1 |
| CvACR3;2 | *Coccomyxa viridis* | Chlorophytes | NCBI | CAK0768907.1 |
| TsACR3 | *Trebouxia sp.* C0004 | Chlorophytes | NCBI | KAL0019817.1 |
| CoACR3 | *Chlorella ohadii* isolate 1 | Chlorophytes | NCBI | KAI7841530.1 |
| BbACR3 | *Botryococcus braunii* Showa | Chlorophytes | Phycocosm | https://phycocosm.jgi.doe.gov/cgi-bin/dispGeneModel?db=Botrbrau1&id=23661 |
| CeACR3 | *Chlamydomonas eustigma* NIES-2499 | Chlorophytes | NCBI | GAX84956.1 |
| ChACR3 | *Chloromonas remiasii* | Chlorophytes | Phycocosm | https://phycocosm.jgi.doe.gov/cgi-bin/dispGeneModel?db=Chlremi1&id=2447160 |
| LsACR3 | *Limnomonas spitsbergensis* CCryo_02-99_CH | Chlorophytes | Phycocosm | https://phycocosm.jgi.doe.gov/cgi-bin/dispGeneModel?db=Limspi1&id=589 |
| SuACR3 | *Sanguina aurantia* | Chlorophytes | NCBI | MEW5305434.1 |
| CzACR3 | *Chromochloris zofingiensis* | Chlorophytes | Phycocosm | https://phycocosm.jgi.doe.gov/cgi-bin/dispGeneModel?db=Chrzof1&id=14366 |
| MmACR3 | *Monoraphidium minutum*  isolate 26B-AM | Chlorophytes | NCBI | KAI8476375.1 |
| RsACR3 | *Raphidocelis subcapitata* NIES-35 | Chlorophytes | NCBI | GBF99582.1 |
| SoACR3 | *Scenedesmus obliquus*  UTEX 3031 | Chlorophytes | NCBI | WIA08162.1 |
| EcACR3 | *Enallax costatus* CCAP 276/31 | Chlorophytes | Phycocosm | https://phycocosm.jgi.doe.gov/cgi-bin/dispGeneModel?db=Enacos1_1&id=6449963 |
| FrACR3 | *Flechtneria rotunda* SEV3VF49 | Chlorophytes | Phycocosm | https://phycocosm.jgi.doe.gov/cgi-bin/dispGeneModel?db=Flerot1_1&id=11394391 |

^1^www.ncbi.nlm.nih.gov

^2^phytozome-next.jgi.doe.gov

^3^gigadb.org/dataset/100613

^4^datadryad.org/stash/dataset/doi:10.5061/dryad.ht76hdrdr

^5^datadryad.org/stash/dataset/doi:10.5061/dryad.0vm37

^6^https://doi.org/10.1016/j.jhazmat.2023.132034.

^7^fernbase.org

^8^www.hornworts.uzh.ch/en/hornwort-genomes.html

^9^phycocosm.jgi.doe.gov

**Table S2. Oligonucleotides used in this work.**

| **Name** | **Sequence 5’ to 3’** |
| --- | --- |
| **Mutagenesis:** | |
| C29A-fw | GGTGAAGTTGTTGTGAAGGCTAAGCCCTTGTGGGACGA |
| C29A-rv | TCGTCCCACAAGGGCTTAGCCTTCACAACAACTTCACC |
| L45A-fw | GAGCGCGAAGGATGAGGCGAGGTGTCGGTTCTCC |
| L45A-rv | GGAGAACCGACACCTCGCCTCATCCTTCGCGCTC |
| L45F-fw | GAGCGCGAAGGATGAGTTCAGGTGTCGGTT |
| L45F-rv | AACCGACACCTGAACTCATCCTTCGCGCTC |
| L45V-fw | GCGCGAAGGATGAGGTGAGGTGTCGGTTC |
| L45V-rv | GAACCGACACCTCACCTCATCCTTCGCGC |
| R46A-fw | CGCGAAGGATGAGTTGGCGTGTCGGTTCTCCAAG |
| R46A-rv | CTTGGAGAACCGACACGCCAACTCATCCTTCGCG |
| R46N-fw | AGGGAGCGCGAAGGATGAGTTGAATTGTCGGTTCTCC |
| R46N-rv | GGAGAACCGACAATTCAACTCATCCTTCGCGCTCCCT |
| R46S-fw | CGAAGGATGAGTTGAGCTGTCGGTTCTCCAAGG |
| R46S-rv | CCTTGGAGAACCGACAGCTCAACTCATCCTTCG |
| R46T-fw | GCGAAGGATGAGTTGACGTGTCGGTTCTCCAAG |
| R46T-rv | CTTGGAGAACCGACACGTCAACTCATCCTTCGC |
| R46V-fw | GCGCGAAGGATGAGTTGGTGTGTCGGTTCTCCAAGG |
| R46V-rv | CCTTGGAGAACCGACACACCAACTCATCCTTCGCGC |
| C47A-fw | GCGAAGGATGAGTTGAGGGCTCGGTTCTCCAAGGAATC |
| C47A-rv | GATTCCTTGGAGAACCGAGCCCTCAACTCATCCTTCGC |
| R48A-fw | AAGGATGAGTTGAGGTGTGCGTTCTCCAAGGAATCGAG |
| R48A-rv | CTCGATTCCTTGGAGAACGCACACCTCAACTCATCCTT |
| R48D-fw | GCGAAGGATGAGTTGAGGTGTGATTTCTCCAAGGAATCGAGGAAT |
| R48D-rv | ATTCCTCGATTCCTTGGAGAAATCACACCTCAACTCATCCTTCGC |
| F49A-fw | GATGAGTTGAGGTGTCGGGCCTCCAAGGAATCGAGGAA |
| F49A-rv | TTCCTCGATTCCTTGGAGGCCCGACACCTCAACTCATC |
| F49I-fw | TGAGTTGAGGTGTCGGATCTCCAAGGAATCGAG |
| F49I-rv | CTCGATTCCTTGGAGATCCGACACCTCAACTCA |
| F49L-fw | ATGAGTTGAGGTGTCGGTTATCCAAGGAATCGAGG |
| F49L-rv | CCTCGATTCCTTGGATAACCGACACCTCAACTCAT |
| F49V-fw | TGAGTTGAGGTGTCGGGTCTCCAAGGAATCGAG |
| F49V-rv | CTCGATTCCTTGGAGACCCGACACCTCAACTCA |
| C71A-fw | CAATGCTGAAGGGTTTCTGGCTAAATGTAGTGCCGAGCTT |
| C71A-rv | AAGCTCGGCACTACATTTAGCCAGAAACCCTTCAGCATTG |
| C73A-fw | ACAATGCTGAAGGGTTTCTGTGTAAAGCTAGTGCCGAGCTTT |
| C73A-rv | AAAGCTCGGCACTAGCTTTACACAGAAACCCTTCAGCATTGT |
| C96A-fw | GCGATGAGCTCTCGGCTGAAGCCCTACCGG |
| C96A-rv | CCGGTAGGGCTTCAGCCGAGAGCTCATCGC |
| C236A-fw | GGTGGGAATCGCTCGCGCCATTGCCATGGTACTT |
| C236A-rv | AAGTACCATGGCAATGGCGCGAGCGATTCCCACC |
| E407A-fw | CAGCCAGTAACAACTTTGCGCTGGCCATAGCTG |
| E407A-rv | CAGCTATGGCCAGCGCAAAGTTGTTACTGGCTG |
| E434A-fw | CGGGCCTCTCATCGCGGTTCCCGTTCTAT |
| E434A-rv | ATAGAACGGGAACCGCGATGAGAGGCCCG |
| L45A-C47A-fw | GGGAGCGCGAAGGATGAGGCGAGGGCTCGGTTCTCCAAGGAATC |
| L45A-C47A-rv | GATTCCTTGGAGAACCGAGCCCTCGCCTCATCCTTCGCGCTCCC |
| R46A-C47A-fw | GAGCGCGAAGGATGAGTTGGCGGCTCGGTTCTCCAAGGAATCG |
| R46A-C47A-rv | CGATTCCTTGGAGAACCGAGCCGCCAACTCATCCTTCGCGCTC |
| C47A-R48A-fw | CGCGAAGGATGAGTTGAGGGCTGCGTTCTCCAAGGAATCGAGG |
| C47A-R48A-rv | CCTCGATTCCTTGGAGAACGCAGCCCTCAACTCATCCTTCGCG |
| L45A-F49A-fw | AGCGCGAAGGATGAGGCGAGGTGTCGGGCCTCCAAGGAATCGAG |
| L45A-F49A-rv | CTCGATTCCTTGGAGGCCCGACACCTCGCCTCATCCTTCGCGCT |
| R46A-F49A-fw | CGCGAAGGATGAGTTGGCGTGTCGGGCCTCCAAGGAATCGAGG |
| R46A-F49A-rv | CCTCGATTCCTTGGAGGCCCGACACGCCAACTCATCCTTCGCG |
| R48A-F49A-fw | CGAAGGATGAGTTGAGGTGTGCGGCCTCCAAGGAATCGAGGAATG |
| R48A-F49A-rv | CATTCCTCGATTCCTTGGAGGCCGCACACCTCAACTCATCCTTCG |
| **Gene cloning:** | |
| BamHI-MpACR3-fw | TAGAACTAGTGGATCCATGAGGGGCTCGGAGATG |
| SalI-MpACR3-rv | ACATGTCGAGGTCGACAGCTTGTTCTTTTGAGAGCC |
| BamHI-PpACR3-fw | TAGAACTAGTGGATCCATGGCAACGCGTCACGAGA |
| SalI-PpACR3-rv | ACATGTCGAGGTCGACGAAGAATTTCTTCCTAAAAAAGAGA |
| BamHI-PvACR3-fw | TAGAACTAGTGGATCCATGGAAAATTCATCCGCTGAAAG |
| SalI-PvACR3-rv | ACATGTCGAGGTCGACAACAGATGGACCCTTTCTTTGG |
| Δ27-98-MpACR3-fw | TTGTTGTGGCCCTACCGGACTCGGAC |
| Δ27-98-MpACR3-rv | GTAGGGCCACAACAACTTCACCAGCTTC |
| N-tail-MpACR3-fw | GTCGACCTCGACATGTCT |
| N-tail-MpACR3-rv | ATCGGTGCGATTCACCAT |
| N-tail-Δ-ScACR3-fw | AAGGGCGTGGTTACAATACTGACTACGATCAAGTC |
| N-tail-Δ-ScACR3-rv | CATGTCGAGGTCGACATTGTTCCATATATAATATGGTT |
| N-tail-ScACR3-fw | GTCGACCTCGACATGTCTAAAGG |
| N-tail-ScACR3-rv | TGTAACCACGCCCTTGACCT |
| N-tail-Δ-MpACR3-fw | GTGAATCGCACCGATGCTGGTCAAATCTACAAACAGC |
| N-tail-Δ-MpACR3-rv | CATGTCGAGGTCGACAGCTTGTTCTTTTGAGAGCC |
| N-tail-MpACR3-pGREG-fw | GAATTCGATAATCAAGCTTATCGATACCGTCGACAATGAGAGGTTCTGAAATGACTGAAATGG |
| N-tail-MpACR3-pGREG-rv | GCGTGACATAACTAATTACATGACTCGAGGTCGACTTAGTCGACTCTGTCCAGCAAAGACAATTGT |
| MpACR3-GUSGFP-fw | GAGAACACGGGGGACTCTAGATGAGGGGCTCGGAGATGACTGAG |
| MpACR3-GUSGFP-rv | GATCGAATTGATCCTCTAGAGCTTGTTCTTTTGAGAGCCATTTCC |
| **qPCR:** | |
| MpAPT1q-fw | CGAAAGCCCAAGAAGCTACC |
| MpAPT1q-rv | GTACCCCCGGTTGCAATAAG |
| mVENUSq-fw | GATGAAGCAGCACGACTTC |
| mVENUSq-rv | CGTCCTTGAAGAAGATGGT |
| MpACR3-fw | GTGCCCTTGCTGCTTTACTT |
| MpACR3-rv | GTTACTGGCTGCAGTGAAGG |

**Table S3. Expression plasmids used in this work.**

| **Name** | **Description** | **Source** |
| --- | --- | --- |
| **Yeast expression:** | | |
| pUG35 | MET17 promoter, yeGFP, CEN, URA3, AmpR | Lab collection |
| pScACR3 | *_pro_MET17:ScACR3-yeGFP* in pUG35 | Lab collection |
| pMpACR3 | *_pro_MET17:*Mp*ACR3-yeGFP* in pUG35 | This study |
| pMpACR3-C29A | *_pro_MET17:*Mp*ACR3-C29A-yeGFP* in pUG35 | This study |
| pMpACR3-L45A | *_pro_MET17:*Mp*ACR3-L45A-yeGFP* in pUG35 | This study |
| pMpACR3-L45F | *_pro_MET17:*Mp*ACR3-L45F-yeGFP* in pUG35 | This study |
| pMpACR3-L45V | *_pro_MET17:*Mp*ACR3-L45V-yeGFP* in pUG35 | This study |
| pMpACR3-R46A | *_pro_MET17:*Mp*ACR3-R46A-yeGFP* in pUG35 | This study |
| pMpACR3-R46N | *_pro_MET17:*Mp*ACR3-R46N-yeGFP* in pUG35 | This study |
| pMpACR3-R46S | *_pro_MET17:*Mp*ACR3-R46S-yeGFP* in pUG35 | This study |
| pMpACR3-R46T | *_pro_MET17:*Mp*ACR3-R46T-yeGFP* in pUG35 | This study |
| pMpACR3-R46V | *_pro_MET17:*Mp*ACR3-R46V-yeGFP* in pUG35 | This study |
| pMpACR3-C47A | *_pro_MET17:*Mp*ACR3-C47A-yeGFP* in pUG35 | This study |
| pMpACR3-R48A | *_pro_MET17:*Mp*ACR3-R48A-yeGFP* in pUG35 | This study |
| pMpACR3-R48D | *_pro_MET17:*Mp*ACR3-R48D-yeGFP* in pUG35 | This study |
| pMpACR3-F49A | *_pro_MET17:*Mp*ACR3-F49A-yeGFP* in pUG35 | This study |
| pMpACR3-F49I | *_pro_MET17:*Mp*ACR3-F49I-yeGFP* in pUG35 | This study |
| pMpACR3-F49L | *_pro_MET17:*Mp*ACR3-F49L-yeGFP* in pUG35 | This study |
| pMpACR3-F49V | *_pro_MET17:*Mp*ACR3-F49V-yeGFP* in pUG35 | This study |
| pMpACR3-C71A | *_pro_MET17:*Mp*ACR3-C71A-yeGFP* in pUG35 | This study |
| pMpACR3-C73A | *_pro_MET17:*Mp*ACR3-C73A-yeGFP* in pUG35 | This study |
| pMpACR3-C96A | *_pro_MET17:*Mp*ACR3-C96A-yeGFP* in pUG35 | This study |
| pMpACR3-L45A,C47A | *_pro_MET17:*Mp*ACR3-L45A,C47A-yeGFP* in pUG35 | This study |
| pMpACR3-L45A,F49A | *_pro_MET17:*Mp*ACR3-L45A,F49A-yeGFP* in pUG35 | This study |
| pMpACR3-R46A,C47A | *_pro_MET17:*Mp*ACR3-R46A,C47A-yeGFP* in pUG35 | This study |
| pMpACR3-R46A,F49A | *_pro_MET17:*Mp*ACR3-R46A,F49A-yeGFP* in pUG35 | This study |
| pMpACR3-C47A,R48A | *_pro_MET17:*Mp*ACR3-C47A,R48A-yeGFP* in pUG35 | This study |
| pMpACR3-R48A,F49A | *_pro_MET17:*Mp*ACR3-R48A,F49A-yeGFP* in pUG35 | This study |
| pMpACR3-AAA | *_pro_MET17:*Mp*ACR3-C29A,C47A,C71A-yeGFP* in pUG35 | This study |
| pN_Sc_-MpACR3 | *_pro_MET17:ScACR3_1-22aa_-MpACR3*-*_111-457aa_-yeGFP* in pUG35 | This study |
| pN_Mp_-ScACR3 | *_pro_MET17:*Mp*ACR3_1-110aa_-ScACR3_23-406aa_-yeGFP* in pUG35 | This study |
| pMpACR3-Δ27-98 | *_pro_MET17:*Mp*ACR3*-Δ*27-98aa-yeGFP* in pUG35 | This study |
| pGREG535 | *GAL1* promoter, 7×HA, *CEN*, *LEU2*, AmpR | EUROSCARF |
| p7HA-MpACR3-N-tail | *_pro_GAL1:7xHA-*Mp*ACR3_1-123aa_* in pGREG535 | This study |
| p7HA-MpACR3-AAA-N-tail | *_pro_GAL1:7xHA -*Mp*ACR3_1-123aa_-C29A,C47A,C71A*  in pGREG535 | This study |
| p7HA-MpACR3-Cys-null-N-tail | *_pro_GAL1:7xHA-*Mp*ACR3_1-123aa_-C29A,C47A,C71A,C73A,C96A* in pGREG535 | This study |
| ***E. coli* expression:** | | |
| pGEX4T-1 | tac promoter, GST, lacI, AmpR | BioCat GmbH |
| pGST-MpACR3-N-tail | *_pro_tac:GST-*Mp*ACR3_1-123aa_* in pGEX4T-1 | This study |
| pGST-MpACR3-AAA-N-tail | *_pro_tac:GST*-Mp*ACR3_1-123aa_* -*C29A,C47A,C71A* in pGEX4T-1 | This study |
| pGST-MpACR3-Cys-null-N-tail | *_pro_tac:GST*-Mp*ACR3_1-123aa_* -*C29A,C47A,C71A,C73A,C96A* in pGEX4T-1 | This study |
| **Plant expression:** | | |
| pBy10-MpACR3 | *_pro_2×35S:*Mp*ACR3-mVenus,* HygR, KanR | This study |
| pBy10-MpACR3-C47A | *_pro_2×35S:*Mp*ACR3-C47A-mVenus,* HygR, KanR | This study |
| pBy10-MpACR3-R46A | *_pro_2×35S:*Mp*ACR3-R46A-mVenus,* HygR, KanR | This study |
| pBy10-MpACR3-R46A,C47A | *_pro_2×35S:*Mp*ACR3-R46A,C47A-mVenus*, HygR, KanR | This study |
| pBy10-MpACR3-AAA | *_pro_2×35S:*Mp*ACR3-C29A,C47A,C71A-mVenus,* HygR, KanR | This study |
| pBy10-MpACR3-R46A,AAA | *_pro_2×35S:*Mp*ACR3-C29A,R46A,C47A,C71A-mVenus,* HygR, KanR | This study |
| pCsA-MpACR3-ST-mScarlet | *_pro_2×35S:*Mp*ACR3-mVenus, _pro_UbE2:52aaST-mScarlet*,  HygR, SpecR | This study |
| pBy10-MpACR3-ER-mTurq2 | *_pro_2×35S:*Mp*ACR3-mVenus,*  *_pro_*Mp*UbE2:ER-mTurq2(HDEL)*, HygR, SpecR | This study |
| pBy10-proMpACR3-MpACR3 | *_pro_*Mp*ACR3:*Mp*ACR3-mVenus,* HygR, SpecR | This study |
| pGFPGUSPlus | CaMV 35S promoter, EGFP, GUSplus^TM^, HygR, KanR | Addgene |
| pGFPGUSPlus-MpACR3 | *_pro_35S:*Mp*ACR3-EGFP* in pGFPGUSPlus | This study |
