# Supplementary material for: Arsenic-sensing domain controls ACR3 transporter trafficking in Marchantia polymorpha
Source: Plant Cell. 2026 Oct 6;38(10):koag306. doi: 10.1093/plcell/koag306 (PMC13674352; doi:10.1093/plcell/koag306)
Supplement: Supplementary file 1 [file koag306_supplementary_data.zip › Supplementary_figures_Bonter_et_al.docx]

**Arsenic-sensing domain controls ACR3 transporter trafficking in *Marchantia polymorpha***

Ignacy Bonter^1,2,ǂ^, Katarzyna Mizio^1,ǂ^, Alicja Dolzblasz^3^, Paulina Tomaszewska^1,4^, Kacper Zbieralski^1^, Jacek Staszewski^1^, Donata Wawrzycka^1^, Anna Reymer^5,6^, Wojciech Bialek^7^, Verena Kriechbaumer^8^, Jim Haseloff^2^, Robert Wysocki^1^, Ewa Maciaszczyk-Dziubinska^1,*^

^1^Department of Genetics and Cell Physiology, Faculty of Biological Sciences, University of Wroclaw, 50-328 Wroclaw, Poland

^2^Department of Plant Sciences, University of Cambridge, Downing Street, Cambridge, CB2 3EA, United Kingdom

^3^Department of Plant Developmental Biology, Faculty of Biological Sciences, University of Wroclaw, 50-328 Wroclaw, Poland

^4^Department of Genetics, Genomics and Cancer Sciences, University of Leicester, LE1 7RH, Leicester, United Kingdom

^5^Department of Chemistry and Molecular Biology, University of Gothenburg, 405 30 Göteborg, Sweden

^6^Institute for Advanced Biosciences (Inserm U1209), Site Santé – Domaine de la Merci, 38700 La Tronche, France

^7^Department of Biophysics, Faculty of Biotechnology, University of Wroclaw, 50-383 Wroclaw, Poland

^8^School of Biological and Medical Sciences and Centre for Bioimaging, Oxford Brookes University, Oxford OX3 0BP, United Kingdom

*Corresponding author: Ewa Maciaszczyk-Dziubinska, Department of Genetics and Cell Physiology, Faculty of Biological Sciences, University of Wroclaw, 50-328 Wroclaw, Poland. Email: ewa.maciaszczyk-dziubinska@uwr.edu.pl

^ǂ^These authors contributed equally to this article.

**Supplementary Figures S1 to S19**

**Supplementary Tables S1 to S3**

**Figure S1.** **Multiple sequence alignment of ACR3 proteins.** CgAcr3, *Corynebacterium glutamicum* (NCBI accession WP_011265757.1); ScAcr3, *Saccharomyces cerevisiae* (NCBI accession DAA11615.1); PvACR3, *Pteris vittata* (NCBI accession ADP20955.1); MpACR3, *Marchantia polymorpha* (NCBI accession OAE35577.1). Predicted transmembrane regions (TM) are marked with black bars. Conserved residues that are crucial for As(III) transport activity of ScAcr3 are highlighted in black. Multiple sequence alignment was generated using the Clustal Omega server (https://www.ebi.ac.uk/Tools/msa/clustalo/). Transmembrane regions were determined using the Consensus Constrained TOPology prediction (CCTOP; <http://cctop.ttk.hu>). This figure supports Fig. 1.

Transport domain

(TM3-5, TM8-10)

Panel domain

(TM1-2, TM6-7)

*****

N-terminus

C-terminus

Cytosolic domain

**Figure S2.** **AlphaFold-predicted structure of MpACR3 (Entry ID A0A176WR38).** The structure was colored based on the AlphaFold per-residue confidence score (pLDDT), where the intervals of confidence are defined as follows: very high (pLDDT > 90, dark blue color), high (90 > pLDDT > 70; blue color), low (70 > pLDDT > 50, yellow color), and very low (pLDDT < 50; peach color). The asterisk indicates the TM4-TM9 crossover region. This figure supports Figs. 1 and 7.

**Figure S3.** **Multiple sequence alignment of long (>100 residues), cysteine-rich N-terminal tails of plant ACR3 proteins.** Columns with at least 25% sequence identity are highlighted. Cysteine residues are highlighted in black. Conserved residues of MpACR3 analyzed in this work are marked with asterisks. Multiple sequence alignment was generated using the Clustal Omega server ([https://www.ebi.ac.uk/Tools/msa/clustalo](https://www.ebi.ac.uk/jdispatcher/msa/clustalo)/) and manually adjusted. Sequence details are provided in Table S1. This figure supports Figs. 7, 9, and 10.

**Figure S3 continued. Multiple sequence alignment of long (>100 residues), cysteine-rich N-terminal tails of plant ACR3 proteins.** Columns with at least 25% sequence identity are highlighted. Cysteine residues are highlighted in black. Conserved residues of MpACR3 analyzed in this work are marked with asterisks. Multiple sequence alignment was generated using the Clustal Omega server ([https://www.ebi.ac.uk/Tools/msa/clustalo](https://www.ebi.ac.uk/jdispatcher/msa/clustalo)/) and manually adjusted. Sequence details are provided in Table S1. This figure supports Figs. 7, 9, and 10.

**Figure S4. Multiple sequence alignment of short (<100 residues) N-terminal tails of plant ACR3 proteins.** Columns with at least 25% sequence identity are highlighted. Cysteine residues are highlighted in black. Multiple sequence alignment was generated using the Clustal Omega server (<https://www.ebi.ac.uk/jdispatcher/msa/clustalo>) and manually adjusted. Sequence details are provided in Table S1. This figure supports Fig. 7.

**Figure S5.** **Stability of MpACR3-GFP in yeast cells.** At time zero, cycloheximide (CHX) was added to a final concentration of 0.1 mg/mL to inhibit protein synthesis in cultures supplemented with 0.1 mM As(III) or left untreated. Total protein extracts were analyzed by Western blotting using anti-GFP antibodies to detect MpACR3-GFP. Anti-Pgk1 immunoblotting served as a loading control. This figure supports Fig. 1.

**Figure S6. No phenotypic differences are observed between *_pro_35S:*Mp*ACR3-GFP* transgenic lines and wild-type *A. thaliana*.** Phenotypic comparison of *_pro_35S:*Mp*ACR3-GFP* transgenic lines T21 and T27, and wild-type control plants (WT, Col-0). Plants were grown in soil under long-day conditions (LD, 22°C). The indicated growth parameters were measured or counted after plants had completed growth. This figure supports Fig. 2.

**Figure S7. Imaging of transgenic Arabidopsis roots expressing *_pro_35S:*Mp*ACR3-GFP* using ZEISS Lattice Lightsheet 7 microscopy.** Root images from the T21 and T27 lines are shown as 3D reconstructions or representative 2D single optical sections (bottom panel). 4-day-old seedlings were transferred to control MS medium or MS medium supplemented with 25 µM As(III) or 1000 µM As(V) and incubated for an additional 24 h before imaging. Scale bars = 50 µm for 3D reconstructions and 15 µm for 2D optical sections. This figure supports Fig. 2.

**Figure S8. Overexpression of Mp*ACR3-mVenus* confers increased arsenic tolerance in Marchantia.** **(a)** Western blot analysis of MpACR3-mVenus protein levels in Marchantia transgenic lines expressing the *_pro_2×35S:*Mp*ACR3-mVenus* transgene, MpACR3-2 and MpACR3-3, grown for 3 weeks under control conditions or in the presence of the indicated concentrations of As(III) or As(V). Protein extract from wild-type Marchantia (Cam-1) was used as a negative control. Ponceau S staining served as a protein loading control. **(b)** Comparative growth of wild-type Cam-1 and transgenic MpACR3-1, MpACR3-2, and MpACR3-3 lines grown in the absence or presence of the indicated concentration of As(III) for 3 weeks. Numbers correspond to transgenic line names. Scale bars = 1 cm. **(c, d)** Arsenic accumulation in Cam-1, MpACR3-2, and MpACR3-3 gametophytes grown in the presence of indicated concentrations of As(III) **(c)** or As(V) **(d)** for 3 weeks. Error bars represent mean ± SD (n = 3 biological replicates; each replicate consisted of pooled thallus tissue from an independent experiment). Different letters indicate statistically significant differences determined by Welch’s one-way ANOVA followed by Games-Howell post hoc test (*P*-value < 0.01) for **(c)** and one-way ANOVA followed by Tukey’s HSD test (*P*-value < 0.001) for **(d).** This figure supports Fig. 4.

**Figure S9.** **Time-lapse imaging of MpACR3-mVenus relocalization from Golgi bodies to the plasma membrane in Marchantia.** Selected frames from a time-lapse series showing MpACR3-mVenus relocalization after As(III) addition are shown; the full sequence is provided in Video S1. A Marchantia gemma expressing *_pro_2×35S:*Mp*ACR3-mVenus* was exposed to 100 μM As(III) at time point 0 and imaged for 15 min. MpACR3-mVenus, green; chloroplasts, magenta. Scale bar = 25 µm. This figure supports Fig. 5.

**Figure S10. Mp*ACR3* expression is increased in the presence of arsenic in Marchantia.** Total RNA was isolated from *M*. *polymorpha* Cam-1 gametophytes grown on solid 0.5*×* Gamborg B5 medium in the absence (control) or presence of indicated concentrations of As(III) and As(V) for two weeks. Mp*APT* (adenine phosphoribosyltransferase) was used as a reference gene to normalize expression data of Mp*ACR3*. Error bars represent mean ± SD (n = 3 biological replicates; each replicate consisted of pooled thallus tissue from an independent experiment). Statistical significance was assessed using one-way ANOVA followed by Dunnett’s multiple comparison test against the untreated control (*, *P*-value < 0.05; **, *P*-value < 0.01; ***, *P*-value < 0.001). This figure supports Figs. 4 and 5.

**Figure S11. Trypsin susceptibility of the MpACR3 N-terminal domain. (a)** Protein lysates were prepared from *acr3*Δ cells expressing the indicated HA-tagged MpACR3 N-terminal domain variants: wild-type, WT; C29A C47A C71A, AAA; or C29A C47A C71A C73A C96A, Cys-null. Cells were treated with 0.5 mM As(III) or left untreated prior to protein extraction. Lysates were then exposed to increasing concentrations of trypsin, followed by Western blot analysis using anti-HA antibodies. Numbers below the Western blot lanes indicate relative protein levels determined from band intensities using ImageJ and normalized to the corresponding mock-treated samples.**(b, c)** Quantification of 7HA-MpACR3-N-terminal domain protein levels after treatment with 1 μg/mL **(b)** or 5 μg/mL **(c)** trypsin for 1 h. Data are presented as mean ± SD from three or four biological replicates from independent experiments, as indicated. Statistical significance was assessed by two-way ANOVA followed by planned post hoc comparisons with Holm correction. Different letters indicate statistically significant differences (*P*-value < 0.05). This figure supports Fig. 8.

**Figure S12.** **As(III) tolerance of Marchantia gemmalings.** Thallus total area was determined for indicated Marchantia lines grown for 10 d on 0.5× Gamborg’s B5 medium supplemented with 50 μM As(III). The projected area of the thallus tissue, excluding rhizoids, was measured using Fiji. Three independent lines per genotype, each with 9 thalli, were analyzed. Each dot represents one measurement from a single gemmaling. Error bars represent mean ± SD (n = 9 gemmalings from three independent lines per genotype). For the analysis shown in Fig. 10c, the mean thallus area calculated for each independent line was used as a biological replicate for each genotype. This figure supports Fig. 10c.

**Figure S13.** **Comparison of molecular dynamics trajectory-averaged structures of the MpACR3 N-terminal domain.** The Triple Ala variant is shown in tan, wild-type in pink, and wild-type with Cys-Cys restraints in dark red. This figure supports Fig. 11.

**Figure S14. Root mean square deviation (RMSD) of heavy atoms during all-atom molecular dynamics simulations of the MpACR3 N-terminal domain.** RMSD was calculated over 2000 ns trajectories for the Triple Ala variant, wild type, and wild type with Cys-Cys restraints. This figure supports Fig. 11.

**Figure S15. Root mean square fluctuation (RMSF) per residue of the MpACR3 N-terminal domain**. **(supports Fig. 11).** RMSF was calculated from molecular dynamics trajectories of the Triple Ala variant, wild-type, and wild-type with Cys-Cys restraints.

**Figure S16. Evolution of SG-SG distances for the Cys29–Cys47, Cys29–Cys71, and Cys47–Cys71 pairs (supports Fig. 11).** The first 2000 ns correspond to the molecular dynamics simulation of the wild-type MpACR3 system without restraints. SG-SG distance restraints were switched on at 2000 ns, gradually increased between 2000 and 3000 ns, and then kept constant during the final 2000 ns of the simulation.

**Figure S17. Comparison of secondary structures of the MpACR3 N-terminal domain.** Secondary structures of wild-type, wild-type with Cys-Cys restraints, and the Triple Ala variant were analyzed using the PDBsum web server. This figure supports Fig. 11.

**Figure S18. Conformational differences observed among MpACR3 N-terminal domain models during 2-µs molecular dynamics trajectories.** The Triple Ala variant is shown in tan, wild type in plum, and wild type with Cys-Cys restraints in red. This figure supports Fig. 11.

|  | **Figure S19. Evolution of the secondary structure of the MpACR3 N-terminal domain during molecular dynamics trajectories.** Secondary structure evolution is shown for the Triple Ala variant **(a)**, wild type **(b)**, and wild type with Cys-Cys restraints **(c).** For the restrained wild-type trajectory, Cys-Cys restraints were gradually increased during the first 1000 ns, indicated by the blue vertical line, as described in the Materials and Methods, and then kept constant for the remaining 2000 ns. The color map on the right indicates the assigned secondary-structure elements. The main differences between the Triple Ala variant and wild-type systems were observed in residues 32-45 and 75-85. In the 32-45 region, a short α-helical segment appeared between residues 35 and 40; upon application of the restraints, this helical region extended across residues 32-45. In the 75-85 region, the 3₁₀-helical structure observed in the Triple Ala variant was replaced by a turn in the restrained wild-type system. This figure supports Fig. 11. |
| --- | --- |

**Table S1. Green algal and land plant ACR3 proteins analyzed or discussed in this study.**

| Protein name | Organism | Taxonomic group | Database | Protein identifier |
| --- | --- | --- | --- | --- |
| PsACR3 | *Picea sitchensis* | Gymnosperms | NCBI^1^ | ADE76924.1 |
| TpACR3 | *Thuja plicata* | Gymnosperms | Phytozome^2^ | Thupl.29379155s0008 |
| GbACR3 | *Ginkgo biloba* | Gymnosperms | Gigascience^3^ | Gb_32402 |
| WmACR3 | *Welwitschia mirabilis* | Gymnosperms | Data Dryad^4^ | W.mirabilis.22940 |
| GmACR3 | *Gnetum montanum* | Gymnosperms | Data Dryad^5^ | TnS000269885t05 |
| PvACR3 | *Pteris vittata* | Ferns | NCBI | ADP20955.1 |
| PvACR3;1 | *Pteris vittata* | Ferns | NCBI | ASV64940.1 |
| PvACR3;2 | *Pteris vittata* | Ferns | NCBI | QTG40769.1 |
| PvACR3;3 | *Pteris vittata* | Ferns | NCBI | QTG40770.1 |
| PvACR3;4 | *Pteris vittata* | Ferns | Sun et al. (2023)^6^ | transcript_28534 |
| CrACR3 | *Ceratopteris richardii* | Ferns | NCBI | KAH7279060.1 |
| AnACR3 | *Adiantum nelumboides* | Ferns | NCBI | MCO5594610.1 |
| SaACR3 | *Salvinia cucullata* | Ferns | Fernbase^7^ | Sacu_v1.1_s0024.g008994 |
| AsACR3 | *Alsophila spinulosa* | Ferns | Fernbase | Aspi01Gene69197.t1 |
| MvACR3;1 | *Marsilea vestita* | Ferns | Fernbase | Mvestita_S10g02657-RA |
| MvACR3;2 | *Marsilea vestita* | Ferns | Fernbase | Mvestita_S10g02658-RA |
| DcACR3;1 | *Diphasiastrum complanatum* | Lycophytes | NCBI | KAJ7529772.1 |
| DcACR3;2 | *Diphasiastrum complanatum* | Lycophytes | NCBI | KAJ7529768.1 |
| ItACR3;1 | *Isoetes taiwanensis* | Lycophytes | Fernbase | Itaiw_v1_scaffold_14_t11603-RA |
| ItACR3;2 | *Isoetes taiwanensis* | Lycophytes | Fernbase | Itaiw_v1_scaffold_2_t02514-RA |
| SmACR3 | *Selaginella moellendorffii* | Lycophytes | NCBI | XP_024531516.1 |
| PpACR3 | *Physcomitrium patens* | Mosses | NCBI | XP_024362327.1 |
| CpACR3 | *Ceratodon purpureus* | Mosses | NCBI | KAG0559408.1 |
| SfACR3 | *Sphagnum fallax* | Mosses | NCBI | KAH8961229.1 |
| MpACR3 | *Marchantia polymorpha* | Liverworts | NCBI | OAE35577.1 |
| RfACR3 | *Riccia fluitans* | Liverworts | NCBI | KAL2645082.1 |
| AaACR3 | *Anthoceros agrestis* | Hornworts | Hornwort genomes^8^ | AagrOXF_evm.model.utg000091l.433__434.1 |
| ApACR3 | *Anthoceros punctatus* | Hornworts | Hornwort genomes | Apun_evm.model.utg000254l.51.1 |
| MeACR3 | *Mesotaenium endlicherianum*  SAG 12.97 | Charophytes | Phycocosm^9^ | https://phycocosm.jgi.doe.gov/cgi-bin/dispGeneModel?db=Mesen1_1&id=7825 |
| ClACR3 | *Closterium sp.*  NIES-68 | Charophytes | NCBI | GJP46584.1 |
| KnACR3;1 | *Klebsormidium nitens* NIES-2285 | Charophytes | NCBI | GAQ91548.1 |
| KnACR3;2 | *Klebsormidium nitens* NIES-2285 | Charophytes | NCBI | GAQ89538.1 |
| CsACR3 | *Coccomyxa subellipsoidea*  C-169 | Chlorophytes | Phycocosm | https://phycocosm.jgi.doe.gov/cgi-bin/dispGeneModel?db=Cosub3&id=1208183 |
| CnACR3;1 | *Coccomyxa elongate* SAG216_3B | Chlorophytes | NCBI | CAL8460937.1 |
| CnACR3;2 | *Coccomyxa elongate* SAG216_3B | Chlorophytes | NCBI | CAL8465694.1 |
| CnACR3;3 | *Coccomyxa elongate* SAG216_3B | Chlorophytes | NCBI | CAL8467475.1 |
| CvACR3;1 | *Coccomyxa viridis* | Chlorophytes | NCBI | CAK0787439.1 |
| CvACR3;2 | *Coccomyxa viridis* | Chlorophytes | NCBI | CAK0768907.1 |
| TsACR3 | *Trebouxia sp.* C0004 | Chlorophytes | NCBI | KAL0019817.1 |
| CoACR3 | *Chlorella ohadii* isolate 1 | Chlorophytes | NCBI | KAI7841530.1 |
| BbACR3 | *Botryococcus braunii* Showa | Chlorophytes | Phycocosm | https://phycocosm.jgi.doe.gov/cgi-bin/dispGeneModel?db=Botrbrau1&id=23661 |
| CeACR3 | *Chlamydomonas eustigma* NIES-2499 | Chlorophytes | NCBI | GAX84956.1 |
| ChACR3 | *Chloromonas remiasii* | Chlorophytes | Phycocosm | https://phycocosm.jgi.doe.gov/cgi-bin/dispGeneModel?db=Chlremi1&id=2447160 |
| LsACR3 | *Limnomonas spitsbergensis* CCryo_02-99_CH | Chlorophytes | Phycocosm | https://phycocosm.jgi.doe.gov/cgi-bin/dispGeneModel?db=Limspi1&id=589 |
| SuACR3 | *Sanguina aurantia* | Chlorophytes | NCBI | MEW5305434.1 |
| CzACR3 | *Chromochloris zofingiensis* | Chlorophytes | Phycocosm | https://phycocosm.jgi.doe.gov/cgi-bin/dispGeneModel?db=Chrzof1&id=14366 |
| MmACR3 | *Monoraphidium minutum*  isolate 26B-AM | Chlorophytes | NCBI | KAI8476375.1 |
| RsACR3 | *Raphidocelis subcapitata* NIES-35 | Chlorophytes | NCBI | GBF99582.1 |
| SoACR3 | *Scenedesmus obliquus*  UTEX 3031 | Chlorophytes | NCBI | WIA08162.1 |
| EcACR3 | *Enallax costatus* CCAP 276/31 | Chlorophytes | Phycocosm | https://phycocosm.jgi.doe.gov/cgi-bin/dispGeneModel?db=Enacos1_1&id=6449963 |
| FrACR3 | *Flechtneria rotunda* SEV3VF49 | Chlorophytes | Phycocosm | https://phycocosm.jgi.doe.gov/cgi-bin/dispGeneModel?db=Flerot1_1&id=11394391 |

^1^www.ncbi.nlm.nih.gov

^2^phytozome-next.jgi.doe.gov

^3^gigadb.org/dataset/100613

^4^datadryad.org/stash/dataset/doi:10.5061/dryad.ht76hdrdr

^5^datadryad.org/stash/dataset/doi:10.5061/dryad.0vm37

^6^https://doi.org/10.1016/j.jhazmat.2023.132034.

^7^fernbase.org

^8^www.hornworts.uzh.ch/en/hornwort-genomes.html

^9^phycocosm.jgi.doe.gov

**Table S2. Oligonucleotides used in this work.**

| **Name** | **Sequence 5’ to 3’** |
| --- | --- |
| **Mutagenesis:** | |
| C29A-fw | GGTGAAGTTGTTGTGAAGGCTAAGCCCTTGTGGGACGA |
| C29A-rv | TCGTCCCACAAGGGCTTAGCCTTCACAACAACTTCACC |
| L45A-fw | GAGCGCGAAGGATGAGGCGAGGTGTCGGTTCTCC |
| L45A-rv | GGAGAACCGACACCTCGCCTCATCCTTCGCGCTC |
| L45F-fw | GAGCGCGAAGGATGAGTTCAGGTGTCGGTT |
| L45F-rv | AACCGACACCTGAACTCATCCTTCGCGCTC |
| L45V-fw | GCGCGAAGGATGAGGTGAGGTGTCGGTTC |
| L45V-rv | GAACCGACACCTCACCTCATCCTTCGCGC |
| R46A-fw | CGCGAAGGATGAGTTGGCGTGTCGGTTCTCCAAG |
| R46A-rv | CTTGGAGAACCGACACGCCAACTCATCCTTCGCG |
| R46N-fw | AGGGAGCGCGAAGGATGAGTTGAATTGTCGGTTCTCC |
| R46N-rv | GGAGAACCGACAATTCAACTCATCCTTCGCGCTCCCT |
| R46S-fw | CGAAGGATGAGTTGAGCTGTCGGTTCTCCAAGG |
| R46S-rv | CCTTGGAGAACCGACAGCTCAACTCATCCTTCG |
| R46T-fw | GCGAAGGATGAGTTGACGTGTCGGTTCTCCAAG |
| R46T-rv | CTTGGAGAACCGACACGTCAACTCATCCTTCGC |
| R46V-fw | GCGCGAAGGATGAGTTGGTGTGTCGGTTCTCCAAGG |
| R46V-rv | CCTTGGAGAACCGACACACCAACTCATCCTTCGCGC |
| C47A-fw | GCGAAGGATGAGTTGAGGGCTCGGTTCTCCAAGGAATC |
| C47A-rv | GATTCCTTGGAGAACCGAGCCCTCAACTCATCCTTCGC |
| R48A-fw | AAGGATGAGTTGAGGTGTGCGTTCTCCAAGGAATCGAG |
| R48A-rv | CTCGATTCCTTGGAGAACGCACACCTCAACTCATCCTT |
| R48D-fw | GCGAAGGATGAGTTGAGGTGTGATTTCTCCAAGGAATCGAGGAAT |
| R48D-rv | ATTCCTCGATTCCTTGGAGAAATCACACCTCAACTCATCCTTCGC |
| F49A-fw | GATGAGTTGAGGTGTCGGGCCTCCAAGGAATCGAGGAA |
| F49A-rv | TTCCTCGATTCCTTGGAGGCCCGACACCTCAACTCATC |
| F49I-fw | TGAGTTGAGGTGTCGGATCTCCAAGGAATCGAG |
| F49I-rv | CTCGATTCCTTGGAGATCCGACACCTCAACTCA |
| F49L-fw | ATGAGTTGAGGTGTCGGTTATCCAAGGAATCGAGG |
| F49L-rv | CCTCGATTCCTTGGATAACCGACACCTCAACTCAT |
| F49V-fw | TGAGTTGAGGTGTCGGGTCTCCAAGGAATCGAG |
| F49V-rv | CTCGATTCCTTGGAGACCCGACACCTCAACTCA |
| C71A-fw | CAATGCTGAAGGGTTTCTGGCTAAATGTAGTGCCGAGCTT |
| C71A-rv | AAGCTCGGCACTACATTTAGCCAGAAACCCTTCAGCATTG |
| C73A-fw | ACAATGCTGAAGGGTTTCTGTGTAAAGCTAGTGCCGAGCTTT |
| C73A-rv | AAAGCTCGGCACTAGCTTTACACAGAAACCCTTCAGCATTGT |
| C96A-fw | GCGATGAGCTCTCGGCTGAAGCCCTACCGG |
| C96A-rv | CCGGTAGGGCTTCAGCCGAGAGCTCATCGC |
| C236A-fw | GGTGGGAATCGCTCGCGCCATTGCCATGGTACTT |
| C236A-rv | AAGTACCATGGCAATGGCGCGAGCGATTCCCACC |
| E407A-fw | CAGCCAGTAACAACTTTGCGCTGGCCATAGCTG |
| E407A-rv | CAGCTATGGCCAGCGCAAAGTTGTTACTGGCTG |
| E434A-fw | CGGGCCTCTCATCGCGGTTCCCGTTCTAT |
| E434A-rv | ATAGAACGGGAACCGCGATGAGAGGCCCG |
| L45A-C47A-fw | GGGAGCGCGAAGGATGAGGCGAGGGCTCGGTTCTCCAAGGAATC |
| L45A-C47A-rv | GATTCCTTGGAGAACCGAGCCCTCGCCTCATCCTTCGCGCTCCC |
| R46A-C47A-fw | GAGCGCGAAGGATGAGTTGGCGGCTCGGTTCTCCAAGGAATCG |
| R46A-C47A-rv | CGATTCCTTGGAGAACCGAGCCGCCAACTCATCCTTCGCGCTC |
| C47A-R48A-fw | CGCGAAGGATGAGTTGAGGGCTGCGTTCTCCAAGGAATCGAGG |
| C47A-R48A-rv | CCTCGATTCCTTGGAGAACGCAGCCCTCAACTCATCCTTCGCG |
| L45A-F49A-fw | AGCGCGAAGGATGAGGCGAGGTGTCGGGCCTCCAAGGAATCGAG |
| L45A-F49A-rv | CTCGATTCCTTGGAGGCCCGACACCTCGCCTCATCCTTCGCGCT |
| R46A-F49A-fw | CGCGAAGGATGAGTTGGCGTGTCGGGCCTCCAAGGAATCGAGG |
| R46A-F49A-rv | CCTCGATTCCTTGGAGGCCCGACACGCCAACTCATCCTTCGCG |
| R48A-F49A-fw | CGAAGGATGAGTTGAGGTGTGCGGCCTCCAAGGAATCGAGGAATG |
| R48A-F49A-rv | CATTCCTCGATTCCTTGGAGGCCGCACACCTCAACTCATCCTTCG |
| **Gene cloning:** | |
| BamHI-MpACR3-fw | TAGAACTAGTGGATCCATGAGGGGCTCGGAGATG |
| SalI-MpACR3-rv | ACATGTCGAGGTCGACAGCTTGTTCTTTTGAGAGCC |
| BamHI-PpACR3-fw | TAGAACTAGTGGATCCATGGCAACGCGTCACGAGA |
| SalI-PpACR3-rv | ACATGTCGAGGTCGACGAAGAATTTCTTCCTAAAAAAGAGA |
| BamHI-PvACR3-fw | TAGAACTAGTGGATCCATGGAAAATTCATCCGCTGAAAG |
| SalI-PvACR3-rv | ACATGTCGAGGTCGACAACAGATGGACCCTTTCTTTGG |
| Δ27-98-MpACR3-fw | TTGTTGTGGCCCTACCGGACTCGGAC |
| Δ27-98-MpACR3-rv | GTAGGGCCACAACAACTTCACCAGCTTC |
| N-tail-MpACR3-fw | GTCGACCTCGACATGTCT |
| N-tail-MpACR3-rv | ATCGGTGCGATTCACCAT |
| N-tail-Δ-ScACR3-fw | AAGGGCGTGGTTACAATACTGACTACGATCAAGTC |
| N-tail-Δ-ScACR3-rv | CATGTCGAGGTCGACATTGTTCCATATATAATATGGTT |
| N-tail-ScACR3-fw | GTCGACCTCGACATGTCTAAAGG |
| N-tail-ScACR3-rv | TGTAACCACGCCCTTGACCT |
| N-tail-Δ-MpACR3-fw | GTGAATCGCACCGATGCTGGTCAAATCTACAAACAGC |
| N-tail-Δ-MpACR3-rv | CATGTCGAGGTCGACAGCTTGTTCTTTTGAGAGCC |
| N-tail-MpACR3-pGREG-fw | GAATTCGATAATCAAGCTTATCGATACCGTCGACAATGAGAGGTTCTGAAATGACTGAAATGG |
| N-tail-MpACR3-pGREG-rv | GCGTGACATAACTAATTACATGACTCGAGGTCGACTTAGTCGACTCTGTCCAGCAAAGACAATTGT |
| MpACR3-GUSGFP-fw | GAGAACACGGGGGACTCTAGATGAGGGGCTCGGAGATGACTGAG |
| MpACR3-GUSGFP-rv | GATCGAATTGATCCTCTAGAGCTTGTTCTTTTGAGAGCCATTTCC |
| **qPCR:** | |
| MpAPT1q-fw | CGAAAGCCCAAGAAGCTACC |
| MpAPT1q-rv | GTACCCCCGGTTGCAATAAG |
| mVENUSq-fw | GATGAAGCAGCACGACTTC |
| mVENUSq-rv | CGTCCTTGAAGAAGATGGT |
| MpACR3-fw | GTGCCCTTGCTGCTTTACTT |
| MpACR3-rv | GTTACTGGCTGCAGTGAAGG |

**Table S3. Expression plasmids used in this work.**

| **Name** | **Description** | **Source** |
| --- | --- | --- |
| **Yeast expression:** | | |
| pUG35 | MET17 promoter, yeGFP, CEN, URA3, AmpR | Lab collection |
| pScACR3 | *_pro_MET17:ScACR3-yeGFP* in pUG35 | Lab collection |
| pMpACR3 | *_pro_MET17:*Mp*ACR3-yeGFP* in pUG35 | This study |
| pMpACR3-C29A | *_pro_MET17:*Mp*ACR3-C29A-yeGFP* in pUG35 | This study |
| pMpACR3-L45A | *_pro_MET17:*Mp*ACR3-L45A-yeGFP* in pUG35 | This study |
| pMpACR3-L45F | *_pro_MET17:*Mp*ACR3-L45F-yeGFP* in pUG35 | This study |
| pMpACR3-L45V | *_pro_MET17:*Mp*ACR3-L45V-yeGFP* in pUG35 | This study |
| pMpACR3-R46A | *_pro_MET17:*Mp*ACR3-R46A-yeGFP* in pUG35 | This study |
| pMpACR3-R46N | *_pro_MET17:*Mp*ACR3-R46N-yeGFP* in pUG35 | This study |
| pMpACR3-R46S | *_pro_MET17:*Mp*ACR3-R46S-yeGFP* in pUG35 | This study |
| pMpACR3-R46T | *_pro_MET17:*Mp*ACR3-R46T-yeGFP* in pUG35 | This study |
| pMpACR3-R46V | *_pro_MET17:*Mp*ACR3-R46V-yeGFP* in pUG35 | This study |
| pMpACR3-C47A | *_pro_MET17:*Mp*ACR3-C47A-yeGFP* in pUG35 | This study |
| pMpACR3-R48A | *_pro_MET17:*Mp*ACR3-R48A-yeGFP* in pUG35 | This study |
| pMpACR3-R48D | *_pro_MET17:*Mp*ACR3-R48D-yeGFP* in pUG35 | This study |
| pMpACR3-F49A | *_pro_MET17:*Mp*ACR3-F49A-yeGFP* in pUG35 | This study |
| pMpACR3-F49I | *_pro_MET17:*Mp*ACR3-F49I-yeGFP* in pUG35 | This study |
| pMpACR3-F49L | *_pro_MET17:*Mp*ACR3-F49L-yeGFP* in pUG35 | This study |
| pMpACR3-F49V | *_pro_MET17:*Mp*ACR3-F49V-yeGFP* in pUG35 | This study |
| pMpACR3-C71A | *_pro_MET17:*Mp*ACR3-C71A-yeGFP* in pUG35 | This study |
| pMpACR3-C73A | *_pro_MET17:*Mp*ACR3-C73A-yeGFP* in pUG35 | This study |
| pMpACR3-C96A | *_pro_MET17:*Mp*ACR3-C96A-yeGFP* in pUG35 | This study |
| pMpACR3-L45A,C47A | *_pro_MET17:*Mp*ACR3-L45A,C47A-yeGFP* in pUG35 | This study |
| pMpACR3-L45A,F49A | *_pro_MET17:*Mp*ACR3-L45A,F49A-yeGFP* in pUG35 | This study |
| pMpACR3-R46A,C47A | *_pro_MET17:*Mp*ACR3-R46A,C47A-yeGFP* in pUG35 | This study |
| pMpACR3-R46A,F49A | *_pro_MET17:*Mp*ACR3-R46A,F49A-yeGFP* in pUG35 | This study |
| pMpACR3-C47A,R48A | *_pro_MET17:*Mp*ACR3-C47A,R48A-yeGFP* in pUG35 | This study |
| pMpACR3-R48A,F49A | *_pro_MET17:*Mp*ACR3-R48A,F49A-yeGFP* in pUG35 | This study |
| pMpACR3-AAA | *_pro_MET17:*Mp*ACR3-C29A,C47A,C71A-yeGFP* in pUG35 | This study |
| pN_Sc_-MpACR3 | *_pro_MET17:ScACR3_1-22aa_-MpACR3*-*_111-457aa_-yeGFP* in pUG35 | This study |
| pN_Mp_-ScACR3 | *_pro_MET17:*Mp*ACR3_1-110aa_-ScACR3_23-406aa_-yeGFP* in pUG35 | This study |
| pMpACR3-Δ27-98 | *_pro_MET17:*Mp*ACR3*-Δ*27-98aa-yeGFP* in pUG35 | This study |
| pGREG535 | *GAL1* promoter, 7×HA, *CEN*, *LEU2*, AmpR | EUROSCARF |
| p7HA-MpACR3-N-tail | *_pro_GAL1:7xHA-*Mp*ACR3_1-123aa_* in pGREG535 | This study |
| p7HA-MpACR3-AAA-N-tail | *_pro_GAL1:7xHA -*Mp*ACR3_1-123aa_-C29A,C47A,C71A*  in pGREG535 | This study |
| p7HA-MpACR3-Cys-null-N-tail | *_pro_GAL1:7xHA-*Mp*ACR3_1-123aa_-C29A,C47A,C71A,C73A,C96A* in pGREG535 | This study |
| ***E. coli* expression:** | | |
| pGEX4T-1 | tac promoter, GST, lacI, AmpR | BioCat GmbH |
| pGST-MpACR3-N-tail | *_pro_tac:GST-*Mp*ACR3_1-123aa_* in pGEX4T-1 | This study |
| pGST-MpACR3-AAA-N-tail | *_pro_tac:GST*-Mp*ACR3_1-123aa_* -*C29A,C47A,C71A* in pGEX4T-1 | This study |
| pGST-MpACR3-Cys-null-N-tail | *_pro_tac:GST*-Mp*ACR3_1-123aa_* -*C29A,C47A,C71A,C73A,C96A* in pGEX4T-1 | This study |
| **Plant expression:** | | |
| pBy10-MpACR3 | *_pro_2×35S:*Mp*ACR3-mVenus,* HygR, KanR | This study |
| pBy10-MpACR3-C47A | *_pro_2×35S:*Mp*ACR3-C47A-mVenus,* HygR, KanR | This study |
| pBy10-MpACR3-R46A | *_pro_2×35S:*Mp*ACR3-R46A-mVenus,* HygR, KanR | This study |
| pBy10-MpACR3-R46A,C47A | *_pro_2×35S:*Mp*ACR3-R46A,C47A-mVenus*, HygR, KanR | This study |
| pBy10-MpACR3-AAA | *_pro_2×35S:*Mp*ACR3-C29A,C47A,C71A-mVenus,* HygR, KanR | This study |
| pBy10-MpACR3-R46A,AAA | *_pro_2×35S:*Mp*ACR3-C29A,R46A,C47A,C71A-mVenus,* HygR, KanR | This study |
| pCsA-MpACR3-ST-mScarlet | *_pro_2×35S:*Mp*ACR3-mVenus, _pro_UbE2:52aaST-mScarlet*,  HygR, SpecR | This study |
| pBy10-MpACR3-ER-mTurq2 | *_pro_2×35S:*Mp*ACR3-mVenus,*  *_pro_*Mp*UbE2:ER-mTurq2(HDEL)*, HygR, SpecR | This study |
| pBy10-proMpACR3-MpACR3 | *_pro_*Mp*ACR3:*Mp*ACR3-mVenus,* HygR, SpecR | This study |
| pGFPGUSPlus | CaMV 35S promoter, EGFP, GUSplus^TM^, HygR, KanR | Addgene |
| pGFPGUSPlus-MpACR3 | *_pro_35S:*Mp*ACR3-EGFP* in pGFPGUSPlus | This study |
