# Supplementary material for: Arsenic-sensing domain controls ACR3 transporter trafficking in Marchantia polymorpha
Source: Plant Cell. 2026 Oct 6;38(10):koag306. doi: 10.1093/plcell/koag306 (PMC13674352; doi:10.1093/plcell/koag306)
Supplement: Supplementary file 1 [file koag306_supplementary_data.zip › Video S1 legend.docx]

**Video S1.** **Time-lapse imaging of MpACR3-mVenus relocalization from Golgi bodies to the plasma membrane in Marchantia.** A Marchantia gemma expressing *_pro_2×35S:*Mp*ACR3-mVenus* was exposed to 100 μM As(III) at time point 0 and imaged for 15 min. MpACR3-mVenus, green; chloroplasts, magenta. This video supports Fig. 5.
